# Age-Dependent Decline in Mouse Lung Regeneration with Loss of Lung Fibroblast Clonogenicity and Increased Myofibroblastic Differentiation

**DOI:** 10.1371/journal.pone.0023232

**Published:** 2011-08-30

**Authors:** Julia A. Paxson, Alisha Gruntman, Christopher D. Parkin, Melissa R. Mazan, Airiel Davis, Edward P. Ingenito, Andrew M. Hoffman

**Affiliations:** 1 Tufts University Cummings School of Veterinary Medicine, North Grafton, Massachusetts, United States of America; 2 Center for Neuroscience Research, Tufts University School of Medicine, Boston, Massachusetts, United States of America; 3 Brigham and Women's Hospital, Harvard Medical School, Harvard University, Boston, Massachusetts, United States of America; Comprehensive Pneumology Center, Germany

## Abstract

While aging leads to a reduction in the capacity for regeneration after pneumonectomy (PNX) in most mammals, this biological phenomenon has not been characterized over the lifetime of mice. We measured the age-specific (3, 9, 24 month) effects of PNX on physiology, morphometry, cell proliferation and apoptosis, global gene expression, and lung fibroblast phenotype and clonogenicity in female C57BL6 mice. The data show that only 3 month old mice were fully capable of restoring lung volumes by day 7 and total alveolar surface area by 21 days. By 9 months, the rate of regeneration was slower (with incomplete regeneration by 21 days), and by 24 months there was no regrowth 21 days post-PNX. The early decline in regeneration rate was not associated with changes in alveolar epithelial cell type II (AECII) proliferation or apoptosis rate. However, significant apoptosis and lack of cell proliferation was evident after PNX in both total cells and AECII cells in 24 mo mice. Analysis of gene expression at several time points (1, 3 and 7 days) post-PNX in 9 versus 3 month mice was consistent with a myofibroblast signature (increased Tnc, Lox1, Col3A1, Eln and Tnfrsf12a) and more alpha smooth muscle actin (αSMA) positive myofibroblasts were present after PNX in 9 month than 3 month mice. Isolated lung fibroblasts showed a significant age-dependent loss of clonogenicity. Moreover, lung fibroblasts isolated from 9 and 17 month mice exhibited higher αSMA, Col3A1, Fn1 and S100A expression, and lower expression of the survival gene Mdk consistent with terminal differentiation. These data show that concomitant loss of clonogenicity and progressive myofibroblastic differentiation contributes to the age-dependent decline in the rate of lung regeneration.

## Introduction

The human adult lung has an intrinsically low capacity for regeneration [1]. As with most mammals, the ability of the human lung to respond to pneumonectomy (PNX) by vigorous compensatory lung growth (i.e. ‘lung regeneration’) is greatly diminished after sexual maturity and closure of the boney epiphyses [1]. In contrast to humans, rodents maintain substantial capacity for lung regeneration and somatic growth well beyond the onset of sexual maturity [2], [3]. Whether age, somatic growth, and regenerative mechanisms persist throughout the life of rodents is presently unclear, even though mice are arguably one of the most important model species employed in studies of molecular mechanisms of both aging and tissue regeneration.

In young adult (2–3 month old) mice, regeneration after PNX occurs through growth of new and existing alveoli in the remaining lung lobes, ultimately leading to restoration of volume, surface area, alveolar numbers, and DNA and protein content within 14 days [2], [3]. Previous studies in our lab suggest that modulation of fibroblast functions (paracrine signaling, extracellular matrix synthesis) and proliferation are critical for lung regeneration in young adult mice [4]. A critical initiating factor for both somatic growth and regeneration of the lung is mechanical strain and mechanotransduction through elements of the extracellular matrix produced by fibroblasts such as elastin [5]. Aging is also associated with significant modifications in the mechanical properties of the lung, such as diminution of lung elastic recoil through apparent senile-related loss of elastin fibers [6]. Age-related changes in elastin function and elastic recoil in the mouse lung are accompanied by reduced levels of expression in genes encoding collagen, elastin, and matrix metalloproteinases [7], implying that matrix synthesis and the capacity for remodeling are progressively lost with age. Given the importance of the extracellular matrix to cell homeostasis [8], lineage fate [9], and regenerative capacity [10], the interactive role of fibroblasts and the extracellular matrix in age-dependent regenerative capacity is a compelling issue that is presently unclear.

We hypothesized that the regenerative capacity of the murine lung is progressively lost with age, and that this decline is related to altered homeostatic functions of lung fibroblasts, manifested as significant changes in clonogenicity and phenotype. To examine this, the time course of post-PNX regeneration was characterized in young (3 month), middle aged (9 month) and old (24 month) female mice. It was determined that the earliest decline in the rate of lung regeneration was measurable by 9 months of age, so global gene expression was evaluated in this early transition phase (3 vs. 9 months). The transcriptomic analyses revealed a distinct pattern of fibroblast to myofibroblast differentiation as a function of age and PNX. This finding was accompanied by the observation of a sharp decline in the abundance of clonogenic fibroblasts and a shift toward a myofibroblastic phenotype with increasing donor age. These data have important implications for lung homeostasis and the capacity for regeneration with age.

## Results

### Age-dependent response to pneumonectomy: pulmonary function and total surface area

**Figure 1:**
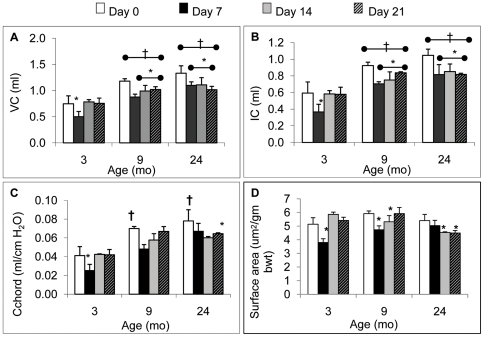
The effect of age on post-PNX lung regeneration vs control. Time course of lung volumes before (day 0 group) and after (days 7, 14, 21) PNX (day 0) in 3, 9 and 24 month mice including **A.** vital capacity (VC), **B.** inspiratory capacity (IC), **C.** dynamic compliance (Cchord) and **D.** total surface area, computed using TLC and MLI (n = 5 mice/group/time point). * P<0.05 when compared to the day 0 (no PNX) group within age category; † P<0.05 vs. 3-mo control group day 0. Data are expressed as means ± SEM.

Aged related changes in pulmonary function in response to PNX were measured in two separate experiments. First, 3, 9 and 24 month old mice were subjected to PNX and lung function parameters were measured and compared before surgery (day 0), and at day 7, 14 and 21 post-PNX. In all groups, *in vivo* lung volumes remained smaller on day 7 (relative to the pre-PNX control group). Inspiratory capacity (IC) and vital capacity (VC) returned to baseline by day 14 only in the 3 month group (**Fig. 1A–B**). Partial return of lung volumes was observed in the 9 month group, with negligible changes observed in the 24 month old group by day 21 post-PNX. Dynamic compliance (Cchord, **Fig. 1C**) rebounded in the 3 month group but remained depressed in the 24 month group after surgery (7–21d). In this first study, a combination of *in vivo* lung volume measurements (TLC) and morphometrics (MLI, without agar inflation to avoid tissue shrinkage) were used to calculate total lung surface area. PNX lowered total surface area, which rebounded by day 14 in the 3 month mice, and later on day 21 in the 9 month mice. Values in 24 month were more variable and remained below baseline on day 14, i.e. only 3 month mice restored the surface area index by day 14 post-PNX. These data clearly point to a physiological reduction in regenerative capacity as early as 9 months, so further analysis of this age period (3–9 months) was examined in sham-controlled experiments, with more accurate measurements of lung surface area.

**Figure 2:**
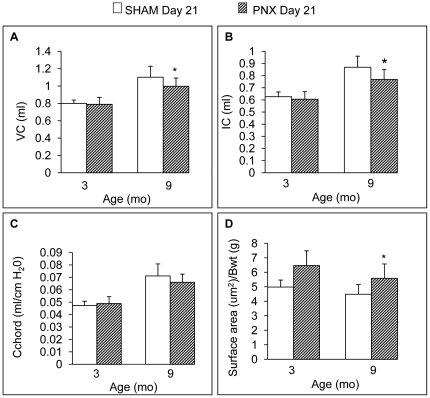
The effect of age on post-PNX lung regeneration vs SHAM. Comparison of lung volumes in SHAM and PNX mice 21 days after surgery in 3 month and 9 month old animals including **A.** vital capacity (VC), **B.** inspiratory capacity (IC), **C.** dynamic compliance (Cchord) and **D.** total surface area, computed using water volume displacement and MLI (agar-inflated) (n = 5 mice/group). Fig. 2A–B: * P<0.05 when compared to the SHAM group within age category. Fig. 2D: * P<0.05 vs. 3 mo PNX (no significant difference between the SHAM lung surface area in 9 mo vs 3 mo mice). Data are expressed as means ± SEM.

In this second series of experiments, 3 and 9 month old mice were subjected to either PNX or sham (SHAM) surgery, and both lung function and morphometry were measured and compared 21 days post-surgery (**Fig. 2**). As in the first experiment, *in vivo* lung volumes (VC, IC) in 3 month old PNX and SHAM mice were indistinguishable, but in the 9 month group PNX caused a significant reduction in lung volumes compared to SHAM-operated mice 21 days after surgery (**Fig. 2A–C**). The total surface area of the right lung was computed using an alternative method in this study (fixation with agar to avoid tissue shrinkage, and water displacement volume measurements), and was significantly larger after PNX (21d) in both 3 and 9 month mice compared to the right lung of SHAM mice in the same age group, consistent with compensatory growth of the remaining lung after PNX. However, total surface area was significantly (P<0.05) smaller in 9 month compared to 3 month mice, demonstrating a partial loss of regenerative capacity in the middle age (9 month) group, consistent with the deficit in lung volumes measured physiologically at 9 months (**Fig. 2D**). These data corroborate the trends observed in the initial experiments comparing PNX to non-operated controls, i.e. partial loss of regenerative capacity at 9 months and complete cessation at 24 months.

### Age-related effects of PNX on AECII and non-AECII cell proliferation and apoptosis

**Figure 3:**
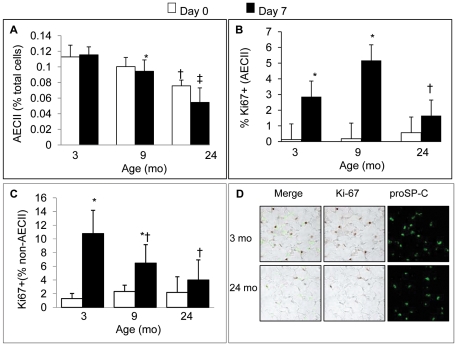
The effect of age on post-PNX cell abundance and proliferation. **A.** The effect of PNX on abundance of alveolar type-2 epithelial cells expressed as a percentage of nucleated cells which stained for proSP-C in each age-group, including pre (day 0) and post (day 7) PNX (n = 5 mice/group). * P<0.05 when compared to the 3 mo (day 7) group. † P<0.05 when compared to the 3 or 9 month control (day 0) group. ‡P<0.05 when compared to the 24 mo control group. Data are expressed as means ± SEM. **B.** Percentage of alveolar type-2 cells (AECII) that were Ki67^pos^ in each age-group pre (day 0) and post (day 7) PNX (n = 5 mice/group). * = P<0.05 when compared to the control within age group; † P<0.05 vs. 9 mo post-PNX data. Data are expressed as means ± SEM; **C.** Percentage of cells in the alveolar region (*excluding* AECII) that were Ki67^pos^ in each age-group control (day 0) and post (day 7) PNX (n = 5 mice/group). * = P<0.05 when compared to the control (day 0) values. † = P<0.05 when compared to the day 7 post-PNX data for the 3 mo group. There was no significant difference between the 9 and 24 mo groups. Data are expressed as means ± SEM; **D.** Representative examples of Ki-67 and pro-SP-C staining in the lung parenchyma after (day 7) PNX in 3 and 24 mo mice.

Peak responses in cell proliferation after PNX were previously shown to occur around day 7 after PNX [11], [12], so the proliferation and apoptosis responses to PNX were evaluated at each age group at this time point and at baseline. At baseline before surgery, the fraction of nucleated cells that were alveolar epithelial type II cells (proSP-C^pos^, AECII) was significantly lower in 24-month versus 3 month mice (**Fig. 3A**), with 9 month animals showing intermediate values. Since AECII are important precursor cells for alveolar epithelial type I cells, this loss of abundance may have important implications for regeneration. However, after PNX there was no difference in AECII densities between 3 and 9 month mice, whereas PNX caused a reduction in AECII in the 24 month mice, consistent with a failure to replenish AECII in this age group. Similarly, PNX caused a sharp increase in the percentage of proliferating (Ki67-positive) AECII cells in 3 and 9 month old mice, a response that was not statistically different between these age groups. However, the percentage of Ki67 positive AECII was significantly lower in 24 month old mice than 3 or 9 month old mice after PNX, and did not increase significantly after PNX (Fig. 3B). Interestingly, cell proliferation in cells other than AECII was more age-dependent (**Fig. 3C**), with a lower percentage of Ki67-positive non-AECII after PNX in 9 month than 3 month old mice. Examples of Ki67 and proSP-C staining in 3 and 24 month old mice (since 3 and 9 month appear the same) are shown in **Fig. 3D**.

**Figure 4:**
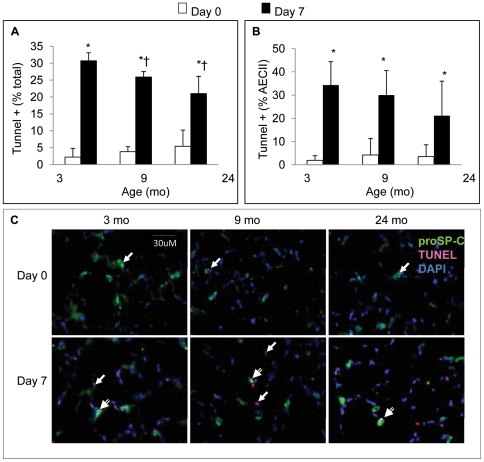
The effect of age on post-PNX cellular apoptosis. Apoptosis as a function of age (3, 9, and 24 mo) and PNX (day 0 vs. 7). **A.** Percentage of nucleated cells that were TUNEL positive; **B.** Percentage of proSP-C positive (AECII) cells that were TUNEL positive. For **A.** and **B.**, * *P*<0.05 compared to day 0 within age group; † P<0.05 compared to d7 in 3 mo old mice; n = 5/group. Data are expressed as means ± SEM; **C.** Examples of TUNEL and proSP-C immunostaining used for enumeration of single (single line arrows) and double (double line arrows) staining (400×mag).

The pattern of apoptosis on day 7 after PNX resembled that of cell proliferation in 3 and 9 month old mice. There was a significant increase in TUNEL positive cells (both total cells and AECII positive) (**Fig. 4A–B**). Unlike the cell proliferation data, TUNEL positive cells increased markedly in 24 month mice after PNX implying that PNX induced more apoptosis than cell proliferation in the aged (24 mo) mice. Representative examples of TUNEL (nuclear), proSP-C (cytoplasmic), and dual (TUNEL/proSP-C) staining are shown in **Fig. 4C**.

These data show that the early decline (between 3 and 9 months of age) in regenerative capacity is not due to a reduction in AECII cell proliferation or an excess in apoptosis; rather, non-AECII progenitor cells show age dependent responses suggesting that among these cells (e.g. endothelial cells, fibroblasts) there was a more age-sensitive population. In contrast, cell proliferation (AECII and non-AECII) and regenerative capacity are both dramatically diminished in aged (24 mo) mice.

### Microarray analysis of mRNA expression patterns in middle aged versus young animals

**Figure 5:**
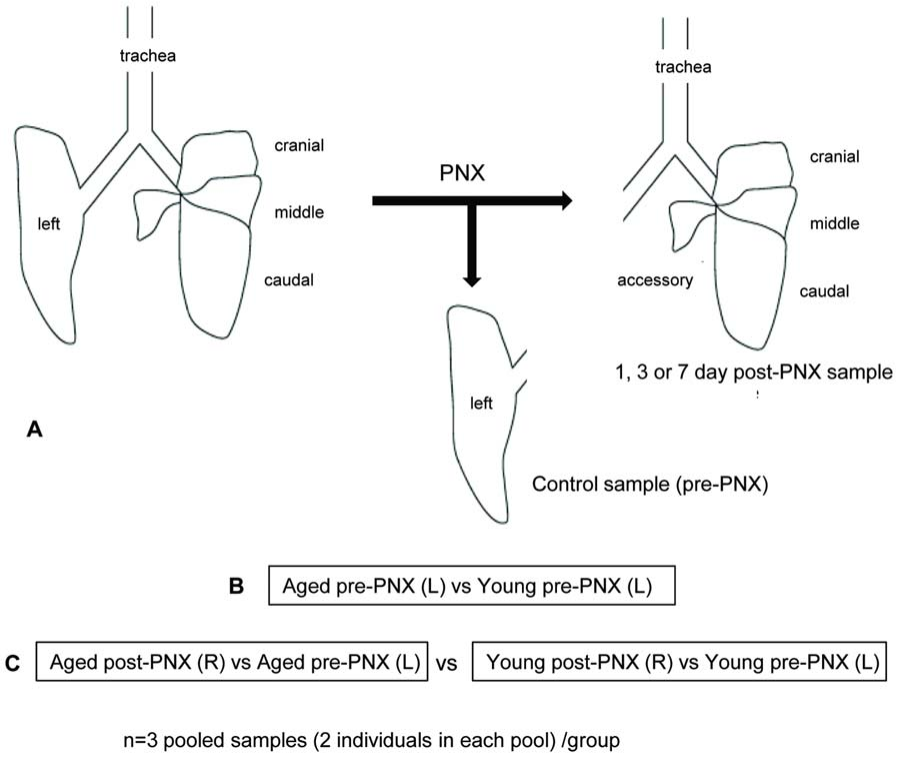
Study design comparing transcriptomic patterns after PNX in 9 vs 3 month mice. **A**: Surgeries. Left lung lobe pneumonectomy (PNX) was performed on 18 young (12 week old) and 18 middle-aged (9 month old) female mice. The left lobe of every animal was preserved in RNAlater at the time of surgery. The mice were sacrificed and the remaining right lung lobes were removed at 1, 3 or 7 days post-surgery (n = 6/group). RNA was prepared from the R and L lung lobes of every animal. For each animal, RNA from the left lung lobe was used as the normalizing RNA for microarray analysis. **B**: Microarray experimental design. At each time point, RNA from two animals was pooled to create one pre-PNX (left lung) sample and one post-PNX (right lung) sample. Therefore, at each time point, 3 pooled samples were created for each group (young pre-PNX; young post-PNX; aged pre-PNX and aged post-PNX). **C.** Microarray data analysis. We generated two groups of data from the microarray analysis. First, to uncover transcripts that are differentially regulated between young and aged animals, we directly compared the aged pre-PNX samples to the young pre-PNX samples (n = 9 pooled samples/group). Second, to compare the effect of aging on lung regeneration post-PNX, we performed a double comparison by first comparing young post-PNX and aged post-PNX to their own control (pre-PNX) for each pooled sample. (n = 6 data sets/time point), and then using a two-class SAM method to compare these aged vs young data sets to identify preferentially expressed genes in aged vs young post-PNX animals.

Microarray analyses of mRNA expression patterns were performed on lung tissue from 9 month versus 3 month mice, before and after PNX. First, gene expression was compared in whole lungs tissues of young (3 month) vs. middle age (9 month) animals obtained at surgery (left, control lung). Second, the global gene expression responses of 3 vs. 9 mo mice were compared after PNX (1, 3 and 7 days) in the right lung. For each animal in the surgical study, the left (excised) lung lobe was used as a control for the corresponding right lung at the end of the study. A schematic of the study design is shown in **Fig. 5**. This design was intended to increase the statistical power for detecting differences in gene expression by comparing pre- and post-PNX lung tissue from the same biological pools (2 animals/pool, 3 pools per group) [13].

Compared to young (3 month) mice control lungs, 192 transcripts were differentially expressed (P<0.05) in the middle-aged (9 month) control lungs prior to any surgery (Table S1 supplemental). After PNX, analysis of differentially regulated transcripts at three different time points (1, 3 and 7 days post-PNX) revealed that 637, 286 and 226 transcripts, respectively, were differentially regulated in 9 month vs. 3 month mice (Tables S2, 3 and S4 - supplemental). Validation of gene expression by microarray was performed using quantitative real time PCR (qPCR) with primers selected for their relevance to the overall model, including genes involved in fibroblastic, myofibroblastic and extracellular matrix signaling (see Table 1).

**Table 1:** Validation of the microarray data (day 1 PNX vs SHAM) using quantitative PCR.

		Quantitative PCR				Microarray			
Gene	BiosciencesCat #	3 mo	P	9 mo	P	3 mo	P	9 mo	P
**Birc5**	PPM03431B	1.8	0.006	1.0	0.921	1.6	0.001	−1.1	0.530
**Cdk1a (p21)**	PPM02901A	1.2	0.510	5.1	0.000	0.6	0.001	4.4	0.000
**Col3A1**	PPM04784B	2.3	0.002	5.3	0.000	2.0	0.000	3.2	0.000
**Eln**	PPM36834A	2.1	0.034	6.1	0.000	1.8	0.000	3.7	0.000
**Il1B**	PPM03109E	2.8	0.023	−1.1	0.857	2.5	0.004	−1.4	0.045
**Lox**	PPM04652A	1.9	0.013	2.5	0.004	1.9	0.000	4.5	0.000
**Retnla**	PPM03005E	5.5	0.000	6.6	0.000	4.3	0.000	7.8	0.000
**Tgfb3**	PPM02993A	1.0	0.990	1.4	0.090	1.1	0.400	1.7	0.000
**Tnc**	PPM03804E	3.4	0.001	11.6	0.000	2.5	0.000	6.7	0.000
**TweakR**	PPM27298A	1.3	0.202	2.7	0.002	1.5	0.014	4.8	0.000

### Age-dependent changes in transcriptomic patterns at baseline prior to PNX

**Figure 6:**
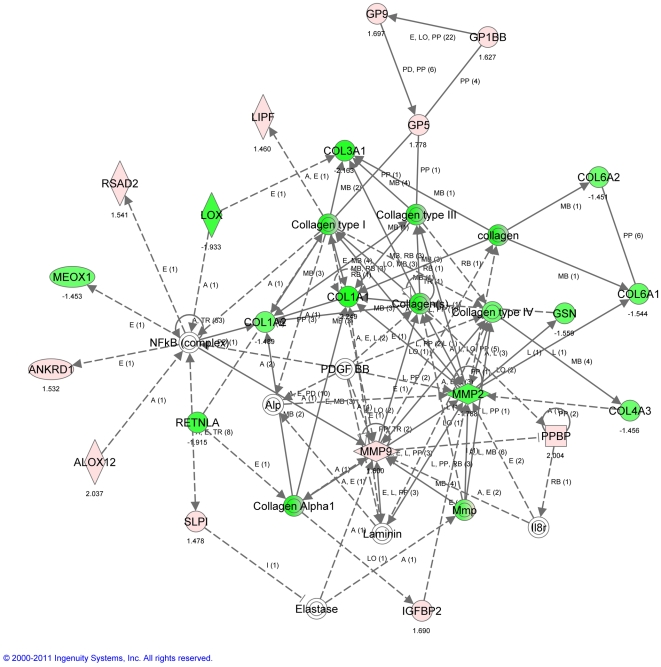
Ingenuity Pathway Analysis (IPA) of transcriptomic patterns without PNX. Top network illustrates transcripts that are significantly modulated in a whole lung analysis of 9 vs. 3 month mice (no surgery). Green indicates down-regulated transcripts, red indicates up-regulated transcripts. Key: The node shapes denote - enzymes 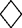 phosphatases 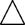 kinases 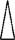 peptidases 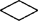 G-protein coupled receptors 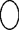 transmembrane receptors 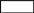 cytokines 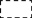 growth factors 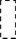 ion channels 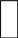 transporter 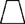 transcription factor 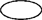 other 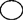.

Ingenuity pathway analysis of transcripts that were differentially regulated between the 9 month and 3 month mice at baseline are consistent with a past study that compared much older (20 or 26 month) to younger (2 month) mice [7], in that matricellular genes were down-regulated including Col1A1, Col3A1, Lox, and Adamts9, (**Fig. 6**). These data are consistent with the hypothesis that as somatic growth declines with age, there is less dependence on extracellular matrix formation and remodeling.

### Age-dependent transcriptomic responses to PNX

**Figure 7:**
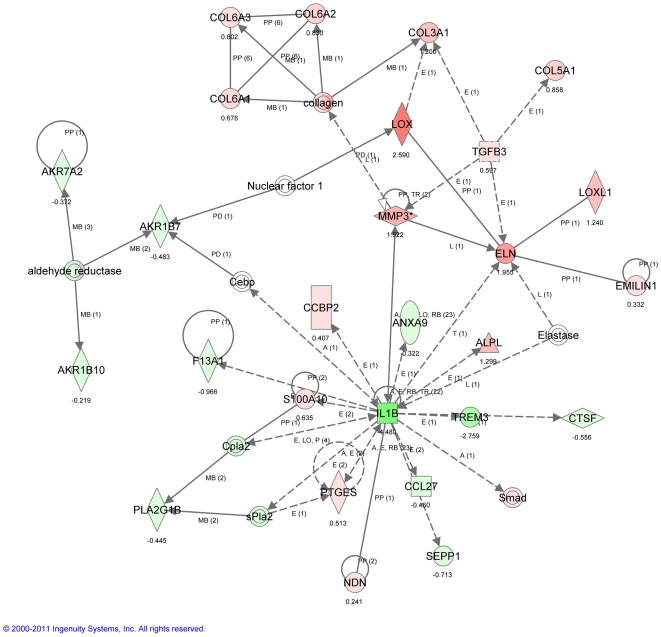
Ingenuity Pathway Analysis (IPA) of transcriptomic patterns 1 day after PNX. Top network illustrate transcripts that are significantly modulated in a whole lung analysis 1 day after PNX in 9 vs. 3 month mice. Green indicates down-regulated transcripts, red indicates up-regulated transcripts. Key: The node shapes denote - enzymes 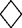 phosphatases 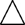 kinases 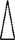 peptidases 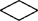 G-protein coupled receptors 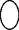 transmembrane receptors 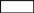 cytokines 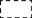 growth factors 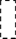 ion channels 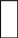 transporter 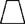 transcription factor 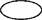 other 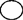.

Pathway analysis of the transcripts that were differentially regulated in 9 month compared to 3 month mice following PNX at three different time points (1, 3 and 7 days) was strongly supportive of phenotypic changes in fibroblasts leading to myofibroblastic differentiation and associated changes in extracellular matrix (ECM) synthesis or remodeling, including over-expression of Tnc, Tnfrs12a, Lox, Tgfβ3, Col3A1, and Retnla (**Figs. 7** **,** **8** **,** **9**). These microarray findings were confirmed using qPCR (**Table 1**). Consistent with the notion that lung regenerative capacity diminished as early as 9 months in mice, Birc5, which is critical to cell proliferation, was up-regulated only in young (3 month old mice) after PNX (**Table 1**). These data suggest that loss of regenerative capacity in aging mice may be associated with a trend towards myofibroblast differentiation of fibroblasts.

**Figure 8:**
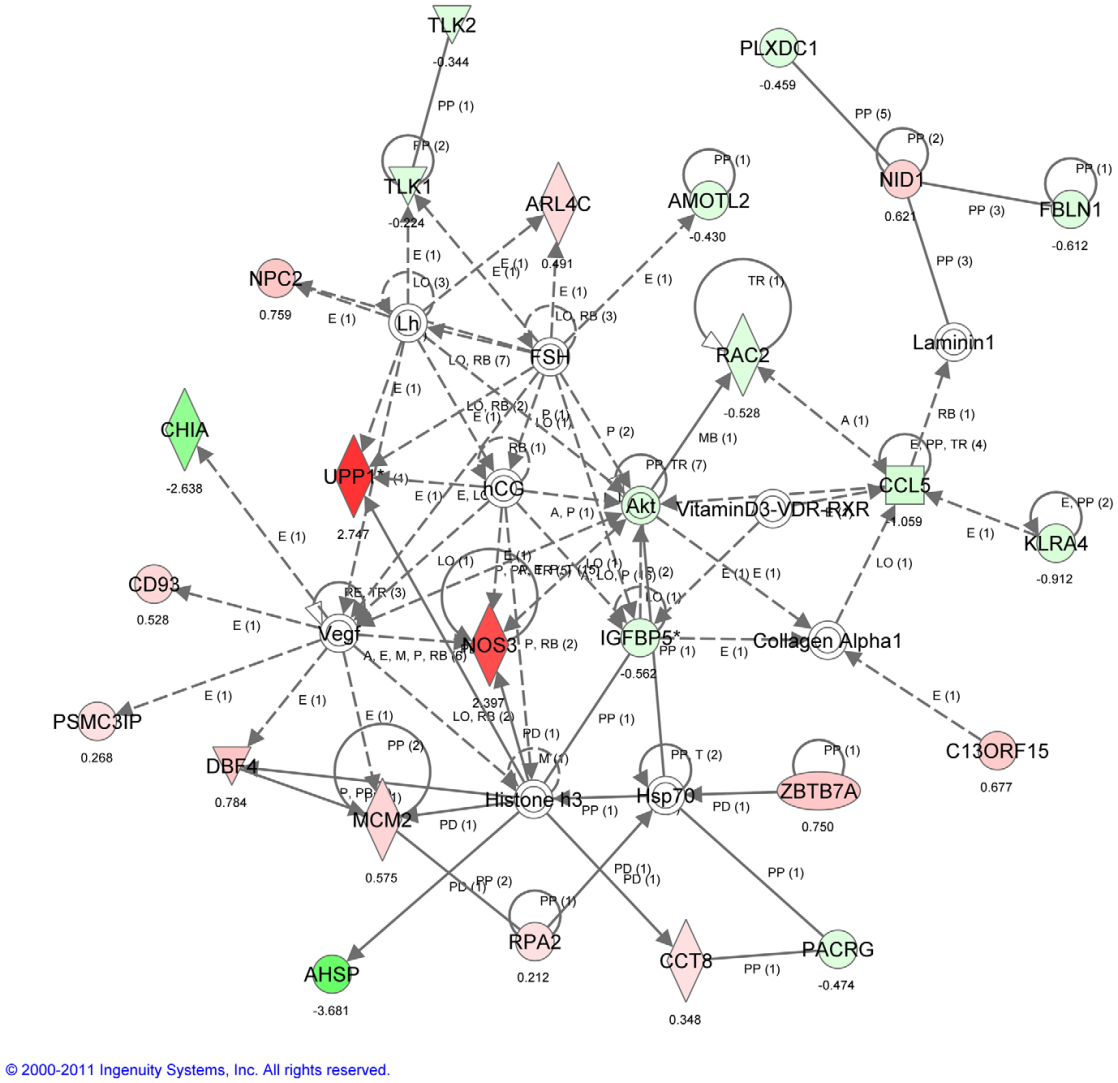
Ingenuity Pathway Analysis (IPA) of transcriptomic patterns 3 days after PNX. Top network illustrates transcripts that are significantly modulated in a whole lung analysis 3 days after PNX in 9 vs. 3 month mice. Green indicates down-regulated transcripts, red indicates up-regulated transcripts. Key: The node shapes denote - enzymes 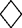 phosphatases 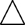 kinases 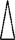 peptidases 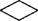 G-protein coupled receptors 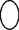 transmembrane receptors 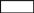 cytokines 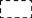 growth factors 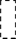 ion channels 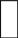 transporter 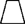 transcription factor 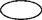 other 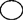.

**Figure 9:**
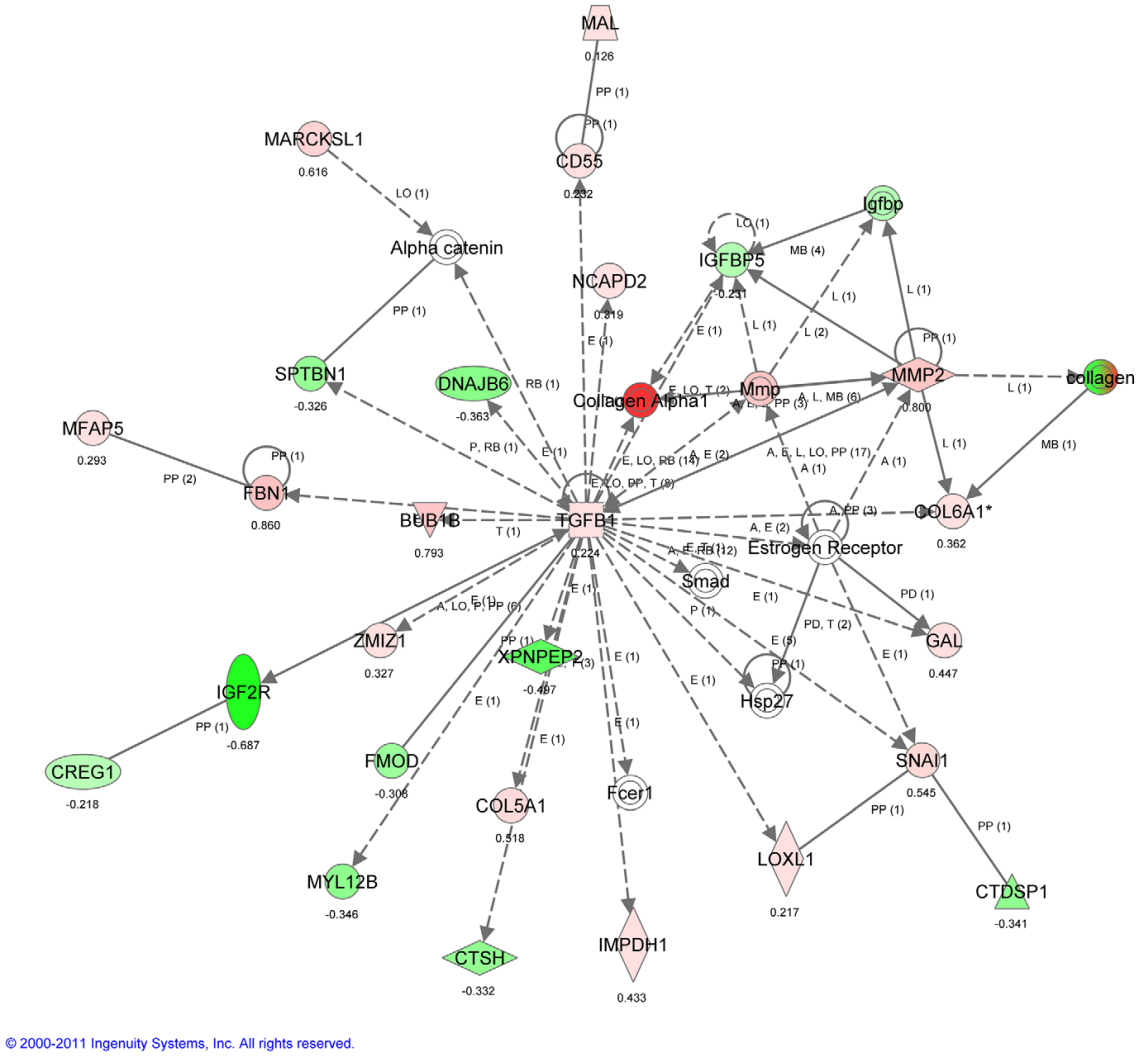
Ingenuity Pathway Analysis (IPA) of transcriptomic patterns 7 days after PNX. Top network illustrates transcripts that are significantly modulated in a whole lung analysis 7 days after PNX in 9 vs. 3 month mice. Green indicates down-regulated transcripts, red indicates up-regulated transcripts. Key: The node shapes denote - enzymes 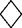 phosphatases 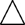 kinases 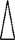 peptidases 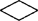 G-protein coupled receptors 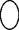 transmembrane receptors 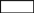 cytokines 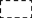 growth factors 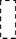 ion channels 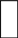 transporter 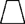 transcription factor 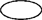 other 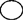.

### Increased αSMA-positive myofibroblasts after PNX in 9 month mice

**Figure 10:**
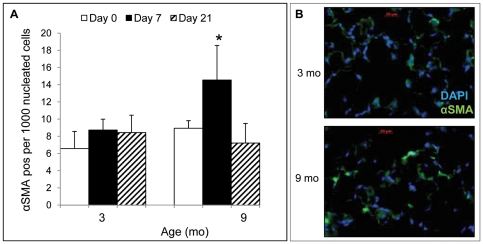
Enumeration of aSMA-positive cells after PNX in 9 vs 3 month mice. **A.** Number of αSMA-positive cells (per 1000 nucleated cells) 0, 7 and 21 days post-PNX in 3 and 9 month mice. Data are mean values ± SEM. *P<0.05 between Day 7 and Day 0; **B.** Micrograph illustrating the difference in αSMA-positive cell abundance in the lung parenchyma at day 7 post-PNX in 3 and 9 month mice. Photomicrograph (400× magnification) with αSMA (green) and DAPI nuclei (blue).

To further investigate the possible role of myofibroblasts in post-PNX lung regeneration in aging mice, the abundance of alpha smooth muscle actin (αSMA)-positive myofibroblasts was measured at 3 time points (day 0 – control pre-PNX, and days 7 and 21 after PNX) in both 3 and 9 month mice using immunohistochemical staining (**Fig. 10**). The data showed that there was no difference in the prevalence of myofibroblasts at baseline, but twice as many αSMA-positive myofibroblasts were present 7 days after PNX in 9 month old mice compared to 3 month old mice (P<0.05), in accordance with the myofibroblastic signature of gene expression by microarray. Interestingly, the myofibroblast abundance within the lung parenchyma returned to baseline by 21 days post-PNX in the 9 month old mice, suggesting that these cells were diminished by apoptosis or other means.

### Extracellular matrix collagen content in 3, 9 and 24 month old mice before and after PNX

**Figure 11:**
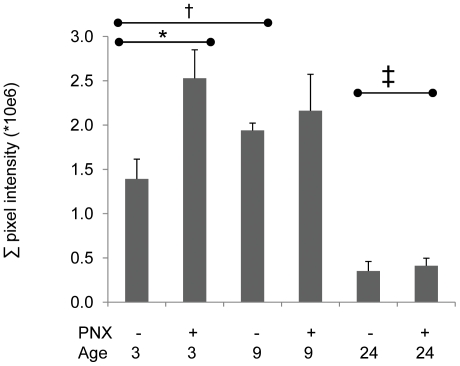
Collagen content after PNX in 3, 9 and 24 month mice. Picrosirius staining as a measure of collagen content in alveolar parenchyma before and after PNX in 3, 9 and 24 month old mice; *P<0.05 3 month pre vs. post, † P<0.05 9 mos vs. 3 mo pre, ‡ P<0.05 24 mo pre or post vs. 3 or 9 mo pre or post; n = 8 mice/group. Data are expressed as mean ± SEM.

Extracellular collagen content in 3, 9 and 24 month old mice before and 21 days after PNX was measured using analysis of digital images of lung parenchyma, color (red) intensity thresholding, and quantification of total pixel intensities in tissue sections (20 fields/section) stained with picrosirius. The data show that more collagen was present at baseline in middle age (9 month) mice than 3 month mice. Furthermore, PNX did not increase collagen content in 9 month mice, whereas collagen content increased substantially after PNX in 3 month mice. In contrast to 3 or 9 month mice, markedly less collagen was present at baseline and in response to PNX in the 24 month old mice (**Fig. 11**). This suggests that significant collagen deposition was associated with complete and ‘normal’ regeneration (3 month), and that older mice were less capable of upscaling deposition of collagen in response to PNX. Whether this increase in collage deposition in young mice was later remodeled, was not investigated in this study.

### Delineation of lung fibroblasts by explant out-growth

**Figure 12:**
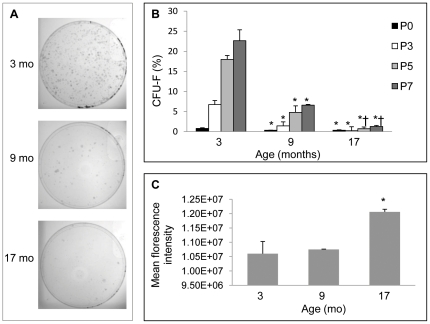
Comparison of the prevalence of colony forming fibroblasts (CFU-F) in the lung of 3, 9 and 17 month old mice. **A.** Examples of representative CFU-F plates derived from 3, 9 and 17 month old mice at passage 5. **B.** Comparison of passages 0 (primary digest), 3, 5, and 7 CFU-F derived from 3, 9 and 17 month old mice. *P<0.05 compared to same passage fibroblasts from 3 month old donor mice; † P<0.05 17 months vs. 9 months same passage fibroblasts; n = 3 experiments/time point. **C.** Comparison of mean florescence intensity obtained by flow cytometry as a measure of cell size between 3, 9 and 17 month cultured lung fibroblasts (passage 4). Cells were gated on forward and side-scatter and 7AAD (negative), and then analyzed for forward scatter mean (30,000 independent events). *P<0.05 compared to fibroblasts from 3 month old mice.

The data presented above indicate that proliferation of non-AECII cells but not AECII cells were diminished in response to PNX in 9 and 24 month old mice. The microarray analysis and quantification of αSMA-positive myofibroblasts in 9 month vs 3 month mice before and after PNX suggested that myofibroblast signaling and cell proliferation were associated with reduced post-PNX regeneration in middle-aged mice. Taken together, these data led to the hypothesis that resident lung fibroblasts may play an important role in the age-dependent decline in lung regenerative capacity. To further investigate the mechanisms by which regeneration is more limited with age, lung fibroblasts were cultured on plastic in basal media (alpha MEM, 15% FBS, 2 mM L-glutamine) either from a single cell suspension (P0, see methods) or from explants (P3, 5, and 7) by the out-growth method [14] in 3, 9 and 17 month mice. In 3 month old mice, explant-derived lung fibroblasts had a significantly higher (P<0.05) CFU-F after initial seeding (P0) and after several different passages (P3, 5, and 7) when compared to 9 or 17 mo old mice (**Fig. 12A–B**). There was a further decline in CFU-F between 9 and 17 mo (P5, P7). The most striking differences were observed after serial passage suggesting that 3 mo old mice fibroblasts have significantly greater capacity for self-renewal. These data suggest that the reduced post-PNX regeneration exhibited byolder mice may be the result of lower prevalence of CFU-F *in vivo*, and lower capacity for self-renewal.

**Figure 13:**
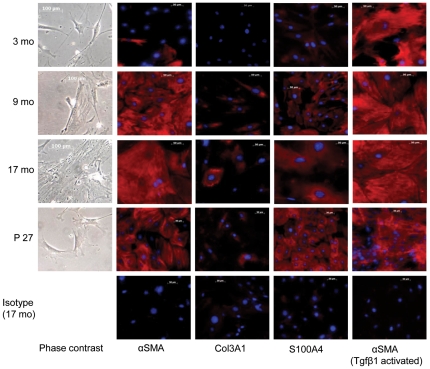
Morphology and phenotype of aging lung fibroblasts. Comparison of explant-grown lung fibroblasts derived from 3, 9 and 17 month old mice, as well as passage 29 (XP27) cells derived from 3 mo mice, grown in 0% serum (200× phase contrast micrographs), as well as lung fibroblasts positive for αSMA, Col3A1 and S100A4 (red), compared to all nucleated cells (blue) (200× micrographs). Representative isotype controls (using fibroblasts from 17 month mice) are included for reference.

In addition to diminished self-renewal potential in older mice, the morphology and molecular phenotype of primary cells from older (9 or 17 month) was consistent with greater fibroblast to myofibroblast differentiation. Older primary cells were larger (**Fig. 12C**), and more frequently expressed αSMA, S100A4, and Col3A1 (**Fig. 13**). Quantitative PCR analysis of these cells supported the observed phenotypic shift toward myofibroblastic cells, including up-regulation of αSMA, Col1A1, Col3A1, Fapα, Fn1 and S100A4 (**Table 2**). That the older fibroblasts were more terminally differentiated was supported by dramatic (200 fold) down-regulation of the survival gene Midkine (Mdk). We also observed a marked down-regulation of the collagen-degrading metalloproteinase Mmp9, demonstrating that Mmp9 expression may be an important feature of highly clonogenic fibroblast populations found in young mice. Furthermore, tumor-associated activation factors Dpp4 and Fapα [15] are generally up-regulated in older fibroblasts compared to the 3 month controls. The ability of lung fibroblasts from these 3 age groups to respond to activation by Tgfβ1 and Tgfβ3 was assessed by αSMA staining and by qPCR gene expression profiling of genes encoding myofibroblast, survival, senescence, and proliferation genes. These cytokines were chosen because microarray analysis demonstrated Tgfβ3 over-expression and Tgfβ1 is commonly implicated in fibroblast differentiation to myofibroblasts [16]. It was found that fibroblasts from 9 and 17 month mice were less capable of responding to activation than fibroblasts from 3 month mice (**Table 3** **,** **4**), including less expression of αSMA/Acta2, Col1A1, Col3A1 or Tnc over baseline, compared to 3 month mice. Activation with Tgfβ3 appeared to have a more pronounced effect than Tgfβ1 at all ages, including dramatic down-regulation of Birc5 compared to non-stimulated control cells. The failure of older fibroblasts to response to Tgfβ1 including up-regulation of collagen mRNA is consistent with the failure to increase collagen deposition after PNX as early as 9 months. Interestingly, late passage (i.e., P27) lung fibroblasts derived from 3 month mice showed modifications in gene expression profile, with higher Birc5 and Col3a1 expression at baseline (compared to passage 6 lung fibroblasts), and significant down-regulation of the fibroblast activation gene Dpp4 after activation with both Tgfβ1 and 3. This suggests that the response to cytokines differs in donor aged vs. replication (*in vitro*) aged fibroblasts from the same mice.

**Table 2:** Relative expression (fold change) of select genes in 9 month, 17 month, and late passage (p27) 3 month lung fibroblasts compared to 3 month control lung fibroblasts.

Gene	Biosciences Cat #	9 mo	P	17 mo	P	P 27	P
**Acta2**	PPM04483A	3.54	0.004	3.92	0.005	2.67	0.008
**Birc5**	PPM03431B	1.29	0.077	1.68	0.006	13.15	0.000
**Cdkn1a**	PPM02901A	1.10	0.516	1.21	0.238	−1.34	0.141
**Col1A1**	PMM03845F	6.32	0.000	6.42	0.002	10.40	0.000
**Col3A1**	PPM04784B	7.93	0.019	5.41	0.020	37.58	0.005
**Dpp4**	PPM05502A	2.30	0.018	−2.44	0.026	2.93	0.000
**Fap α**	PPM28904A	2.26	0.010	2.60	0.012	9.79	0.000
**Fn1**	PPM03786A	4.75	0.000	2.94	0.006	5.35	0.001
**Lox**	PPM04652A	1.35	0.014	2.11	0.000	1.28	0.018
**Midkine**	PPM03800B	−223.56	0.000	−236.18	0.000	−321.24	0.000
**Mmp9**	PPM03661B	−52.57	0.000	−214.79	0.003	−27.04	0.013
**p53**	PPM02931B	−1.34	0.161	−1.18	0.063	1.42	0.030
**S100A4**	PPM03811A	3.15	0.005	3.64	0.000	9.11	0.000
**Tnc**	PPM03804E	−2.26	0.004	1.10	0.236	−3.85	0.000
**TweakR**	PPM27298A	1.07	0.344	1.04	0.443	−1.14	0.105

**Table 3:** Relative expression (fold change) of select genes in 3, 9, 17 month and passage 27 (derived from 3 month mice) lung fibroblasts after activation by Tgfβ1 compared to un-activated age-matched controls.

Gene	3 mo	P	9 mo	P	17 mo	P	P 27	P
**Acta2**	1.79	0.045	1.16	0.263	1.59	0.106	1.14	0.367
**Birc5**	−2.01	0.002	−8.44	0.000	−2.70	0.001	−1.04	0.888
**Cdkn1a**	1.50	0.068	1.87	0.002	1.39	0.128	1.30	0.134
**Col1A1**	2.83	0.003	1.42	0.033	1.73	0.075	1.13	0.380
**Col3A1**	−1.09	0.826	1.13	0.362	1.41	0.326	−1.10	0.667
**Dpp4**	−3.02	0.037	−3.48	0.013	1.61	0.104	−15.06	0.005
**Fap α**	1.03	0.879	−2.60	0.003	−2.24	0.024	−2.85	0.004
**Fn1**	2.18	0.001	1.79	0.002	2.72	0.014	1.14	0.383
**Lox**	2.55	0.001	1.27	0.103	1.59	0.001	1.59	0.069
**Midkine**	−1.34	0.054	−1.24	0.407	−1.21	0.343	1.08	0.822
**Mmp9**	50.41	0.000	8.23	0.001	32.49	0.010	26.58	0.008
**p53**	1.36	0.000	1.48	0.113	1.66	0.047	1.46	0.063
**S100A4**	−2.13	0.006	−2.99	0.009	−1.72	0.049	−1.95	0.061
**Tnc**	2.01	0.000	1.41	0.054	1.04	0.818	1.47	0.019
**TweakR**	1.94	0.000	1.89	0.008	1.75	0.016	2.83	0.008

**Table 4:** Relative expression (fold change) of select genes in 3, 9 17 month and passage 27 (derived from 3 month mice) lung fibroblasts after activation by Tgfβ3 compared to un-activated age-matched controls.

Gene	3 mo	P	9 mo	P	17 mo	P	P27	P
**Acta2**	1.64	0.082	−1.27	0.113	1.574	0.150	−1.41	0.068
**Birc5**	−4.06	0.000	−19.63	0.000	1.017	0.899	−4.37	0.004
**Cdkn1a**	−1.44	0.079	−1.44	0.030	−1.330	0.201	−2.30	0.008
**Col1A1**	1.05	0.704	−2.29	0.005	1.698	0.081	−3.80	0.003
**Col3A1**	1.52	0.342	1.34	0.067	1.031	0.911	−1.46	0.045
**Dpp4**	−4.83	0.001	−5.12	0.002	−7.717	0.098	−28.01	0.001
**Fap α**	−2.36	0.006	−5.33	0.001	152.885	0.000	−7.39	0.000
**Fn1**	−1.85	0.010	−2.27	0.000	−1.002	0.991	−4.46	0.001
**Lox**	1.35	0.047	−1.48	0.008	−1.192	0.594	−1.38	0.085
**Midkine**	−1.68	0.010	−1.66	0.007	−1.362	0.263	−1.25	0.143
**Mmp9**	44.95	0.000	14.73	0.003	64.433	0.001	26.45	0.015
**p53**	−1.28	0.001	−1.37	0.134	1.055	0.693	−1.40	0.027
**S100A4**	−2.25	0.001	−3.35	0.002	−2.193	0.019	−2.59	0.001
**Tnc**	−1.87	0.001	−2.73	0.002	−1.736	0.087	−2.07	0.000
**TweakR**	−1.08	0.331	−1.13	0.144	−348.150	0.002	1.22	0.042

## Discussion

Our data show for the first time that the capacity for lung regeneration in mice is strongly age-dependent, with partial loss of regenerative capacity observed as early as middle age (9 month) mice, and total lack of physiologic or cellular growth observed in old (24 month) mice, similar to the regenerative decline reported in other organs such as the liver [17]. This study focused on event surrounding the transition to diminished regenerative capacity in order to better understand the earliest (upstream) transcriptional and cellular events which might initiate the decline in regenerative capacity. Transcriptomic analysis of young (3 month) vs. middle age (9 month) lung tissue demonstrated that genes encoding ECM are decreased in older (9 month) mice, as has been previously reported in a comparison of young and old (18–24 month) mice [7]. Furthermore, the response to PNX was associated with an increase in the expression of myofibroblast signature genes in 9 month old mice compared to 3 month old mice. The myofibroblastic gene expression associated with the aging lung was typified by an increased prevalence of α-smooth muscle actin (aSMA)-positive lung parenchymal cells following PNX. Moreover, our data suggest that the clonogenicity of lung fibroblasts declined with age, with a phenotypic shift towards myofibroblastic differentiation and less response to activation by Tgfβ1 and 3, consistent with a greater commitment to terminally differentiated myofibroblasts in the stromal cell population of the lung. In summary, these data imply that the aged lung has progressively limited clonogenic cell reserves, which presumably impacts the regenerative capacity of the organ. This assertion is based on the observation that complete regrowth of the lung after resection requires a massive up-scaling of matrix synthesis [11], [12]. A similar age-dependent decline in progenitor cell function has been identified in other organs [18]–[20], and has been linked to declining regenerative capacity [21]. Several studies also suggest that aging is associated with changes in the extracellular matrix [19], [22], and that the proliferative potential and differentiation fate of progenitor cells can be affected by the surrounding matrix [18], [20].

### Age related alterations in lung physiology and morphometry after pneumonectomy

Pneumonectomy resulted in expected volume losses (∼30–35%) which were reversed over 14 days in the 3 month old mice, but remained significantly below pre-PNX (day 0 group) values on days 14 and 21 in the 9 and 24 month mice. On day 21, the 3, 9, and 24 month group mean VC were 103, 89, and 86% of baseline (day 0) values, respectively. Past studies show that removal of the left lung of mice causes a 31–36% loss of total lung volume [2], [3], hence our data suggest that middle aged (9 month) and aged mice (24 month) mice both restored lung volume by growth or adaptation to some extent. In support of the age-dependent changes in regeneration observed in this study, aging between 12–26 wks in both rats and rabbits was characterized by progressive airspace enlargement post-PNX, and thus apparent failure to develop new alveolar septae [23], [24].

In a second *in vivo* study, PNX was compared to SHAM instead of left (control) lungs, and animals were compared between 3 and 9 month mice. Consistent with the first study, only the 3 month animals completely restored lung volumes (VC, IC) and lung compliance by 21 days post-PNX, demonstrating a slower rate of regeneration in the 9 month mice. In study, lungs were formalin fixed at 25 cm pressure using low melt agar to avoid tissue shrinkage, and lung volume was measured using water displacement. Total surface area of the remaining right lung 21 days after PNX was significantly smaller (85% SHAM group) in the 9 month vs. 3 month mice, which is consistent with the changes seen in lung volumes at the same ages.

Accompanying the physiological changes in regenerative capacity in the aging mice was an early (9 month mice) loss of non-AECII cell proliferation and a later (24 month mice) loss in AECII cell proliferation as well. This suggests that either (1) non-AECIIs regulate lung regeneration, (2) non-AECII progenitor cells (endothelial cells, fibroblasts) failed to response to AECII signals, or (3) despite evidence of brisk AECII proliferation at a time when regeneration is slowing, the paracrine signals from AECII such as Fgf7 or Sdf1 [25] were waning. The cause was not determined in this study. A number of past studies show that AECII respond strongly to changes in matrix composition and stretch [5], [26]–[28], which point to a primary mechanism involving the stroma in this model. Since AECII proliferation and apoptosis were both increased in 3 and 9 month animals equivalently, it is further possible that the process of differentiation from AECII to AECI was either diminished or modified qualitatively (e.g. leading to epithelial-mesenchymal transition).

### Age related alterations in lung fibroblast phenotype and function before and after pneumonectomy

Analysis of age-related alterations in global gene expression patterns at baseline (before PNX) supports the concept that aging is associated with a decrease in the production of ECM commensurate with reduced somatic growth, as has been observed in other studies [4], [7]. Due to these significant changes with age alone, we subtracted this effect from the analysis of gene expression after PNX by using mRNA from each biological sample (left lung) as individual controls. This provided biologically and aged matched controls, a technique that has been used to investigate differences in gene expression after hepatic resection [13]. The effect of age on the response of PNX was then examined using pathway analyses. The results show an increase in myofibroblastic-like signaling patterns in 9 month mice relative to the younger 3 month mice including up-regulation of cell signaling (Tgfβ3), matricellular (Lox), and matrix (Tnc, Tnfrsf12A, and Col3A1) genes, at the exclusion of other mechanisms (e.g. differences in growth factor, cytokine or chemokine expression) that might explain differences in growth or regenerative capacity. This pattern in middle age mice is in sharp contrast to the findings in young mice in earlier studies, where mRNA expression during post-PNX regeneration was exemplified by up-regulation of mesenchymal stromal cell signaling and proliferation genes [4], [29] but down-regulation of pro-fibrotic or pro-myofibroblastic genes [4].

Consistent with the post-PNX transcriptomic patterns in 9 vs. 3 month mice, we demonstrated that αSMA-positive parenchymal myofibroblasts were almost twice as abundant 7 days after PNX in 9 month versus 3 month mice. Thus the fibroblast phenotype was shifting to myofibroblasts under the influence of PNX, explicitly in older mice. As myofibroblasts are less replicative and less efficient in ECM synthesis, their function to restore tissue mass and surface area would be expected to be lower. Furthermore, one might expect myofibroblast expansion in the lung to promote fibrosis [30]. However, collagen content of the lung was measured showing that 9 month mice have greater collagen at baseline but do not increase collagen after PNX in contrast to 3 month mice, which have a burst of collagen expression associated with PNX (akin to their greater baseline pre-PNX collagen mRNA synthesis, a feature of somatic growth). Taken together, these data show that aging (i.e., up to 9 months) causes a shift in fibroblast phenotype *in vivo*, toward a more differentiated, less collagen synthetic cell that would be expected to hamper regeneration

Similar to the *in vivo* data, CFU-F grown from lungs of 9 or 17 month old mice were dramatically lower than CFU-F from 3 month old mice. The CFU-F were lower at initial seeding as well as following serial passage, suggesting that both the prevalence of CFU-F *in vivo* and their subsequent capacity for self-renewal are affected by age. Similar age-related declines in clonogenicity have been demonstrated in bone marrow mesenchymal cells [31], [32], with reversal of these changes when aging cells are exposed to young extracellular matrix [33], but has not been previously documented in the lung of normal animals or humans to our knowledge. Furthermore, the morphology and molecular phenotype of fibroblasts derived from older animals was more typical of terminally differentiated myofibroblastic cells, including increased expression of aSMA, Col1A1, Col3A1, and Fn1. That Tgfβ1 and Tgfβ3 had minimal effects to activate these cells further, suggests that these cells are more differentiated, less clonogenic, less pro-regenerative (synthetic), and less able to survive as indicated by dramatically lower Mdk expression. It is also noteworthy that lung fibroblasts from older donors were generally more activated, similar to cancer associated fibroblasts, with increased Fapα and/or Dpp4 expression [15]. Fibroblasts from older donors were different from lung fibroblasts that were cultivated over 27 passages (>70 doublings, ∼6 months). These late passage (P27) fibroblasts displayed higher levels of Birc5 and Fap alpha expression suggesting that highly clonogenic fibroblasts replicated in culture maintain their survival program over many generations [34], but showed greater activation after many passages. Previous studies examining aging versus replicative senescence in mesenchymal stromal cells have also identified changes in transcriptomic profiles of aging stromal cells suggesting that age affects the function of these progenitor populations [35], [36].

According to this study, the natural progression in mice with age is to lose CFU populations of lung fibroblasts, resulting in a significantly smaller pool of replicative progenitor cells capable of differentiation or matrix synthesis. This might explain the lower amount of collagen synthesis in old (24 month) mice at baseline, and the smaller increase in collagen synthesis after PNX *in vivo* as early as 9 months as well as the failure of TGFβ1 to stimulate collagen synthesis in lung fibroblasts from older donors (9 or 17 months).

In conclusion, these data demonstrate the rate of regeneration after PNX is diminished by 9 months in mice and continues to decline further in older animals. Clonogenic, undifferentiated fibroblasts are significantly more abundant in young animals and may be critical to sustain the capacity for post-PNX regeneration, including cell proliferation, matrix synthesis, and paracrine signals. Importantly, the loss of CFU potential with age is associated with greater fibroblast commitment *in vitro*, and evidence of myofibroblast participation *in vivo* in the response to PNX. Therefore, the aging lung differs from other endodermal organs (kidney, liver) that demonstrate a slower decline in regenerative capacity [18], in that the lung CFU reserves appears more limited and more differentiated. Thus attempts to rejuvenate lung regeneration in aging adults may require different strategies such as dedifferentiation or resupply of young lung fibroblasts, or exposure to a young stromal environment.

## Methods

### Study design

All protocols were approved by the Institutional Animal Care and Use Committee at Tufts University (IACUC protocol # G974-08 and G2011-31). The objective of the first part of this study was to compare the physiologic and morphometric, and alveolar epithelial cell responses to unilateral pneumonectomy (PNX) of 3, 9, and 24 mo-old C57BL6 female mice. Time points examined were 0 (control, unoperated), 7, 14, and 21 days post-pneumonectomy (n = 5–6 mice per group). *In vivo* physiologic measurements included vital capacity (VC), inspiratory capacity (IC), functional residual capacity (FRC), and dynamic compliance (Cchord). Mean linear intercept (MLI) was used to characterize volume/surface area of the airspaces. Cell proliferation was estimated by enumerating the density of Ki-67^pos^ type II alveolar epithelial cells (AECII, proSP-C positive) or non-AECII cells. The objective of the second part of this study was to investigate the molecular mechanisms involved in the age-related decline in regenerative capacity in adult (3 month) female C57BL/6 (20–25 g) and middle-aged (9 month) female C57BL/6 (25–35 g) mice (Jackson Laboratories). Two samples were obtained from each mouse; the left lung was removed at day 0 during the pneumonectomy surgery and processed as a control sample; then, the remaining lung tissue was removed at one of three time points post-pneumonectomy (1 day, 3 days, or 7 days). For each of the three time points, two groups of mice were used: (1) aged and (2) young, with six animals in each group. For the microarray experiment, equal amounts of RNA from 2 animals (control and PNX samples) were pooled, resulting in a total of 3 control and 3 PNX microarray samples at each time point (**Fig. 5**).

### Physiology

The extent of compensatory lung regrowth was evaluated by measurement of lung volume subdivisions, respiratory system static compliance, and dynamic airway and tissue mechanics *in vivo*. Mice were anesthetized via intraperitoneal injection with ketamine (50–75 mg/kg) and xylazine (3.8–5 mg/kg). Orotracheal intubation was performed using a 20 g intravenous catheter (1.1×25 mm, BD Insyte). Mice were placed on a digitally controlled mechanical ventilator (AUT6110, Buxco Electronics, Wilmington, NC) in supine position within a flow-type whole body plethysmograph (PLY3111, Buxco Electronics) that controlled forced and passive inflation/deflation maneuvers. The plethysmograph-transducer-amplifier unit was calibrated using a precision volume syringe (by integration of flow) and airway pressure using a water-filled U-manometer (Scireq Corp, Montreal, Canada). Ventilation (room air) was set at 200 breaths/minute, tidal volume equaled 10 mL/kg bwt, and positive end-expiratory pressure (PEEP) 3.0 cm H_2_O except during measurements. For measurement of lung volume subdivisions, the lungs of mice were inflated (25 cm H_2_O) three times prior to measurements for volume history and to promote apnea. The volume of inflation from passive end-expiratory lung volume to 25 cm H_2_O airway pressure was recorded as inspiratory capacity (IC) (XA Biosystem v. 2.9, Buxco Electronics). The volume from a plateau pressure of +25 cm H_2_O to −25 H_2_O was recorded as vital capacity (VC).

### Pneumonectomy

Mice acclimated to their housing for at least 72 hours, were anesthetized and intubated as described above. Prophylactic antibiotics (a single dose of ampicillin sodium, (100 mg/kg SQ) and isotonic electrolyte solution (2 mL of 0.9% sodium chloride SQ) were administered immediately prior to surgery. The left side of the chest was clipped and a sterile aseptic field created using a chlorhexidine/alcohol antiseptic solution. Mice were ventilated with a mechanical ventilator (AUT6110, Buxco Electronics, Wilmington, NC) using the settings described for the physiology measurements with the exceptions of PEEP which was increased to 5 cm H_2_O during the procedure. Sterile scissors were used to incise 7 mm of skin, exposing the left 5^th^ intercostal space, the thorax was then opened using iris scissors and the lung exteriorized carefully using blunt ended forceps. Once lifted such that the left lung hilum was visualized, the mainstem bronchus was ligated using 4-0 silk and the lung distal to this ligature excised. A single interrupted suture of 4-0 polytrimethylene carbonate (Maxon, Davis & Geck, Gosport, UK) was used to close the thorax, and tightened at the end of a sustained lung inflation (25 cm H_2_O) to minimize post-operative pneumothorax. The skin was then also closed using 4-0 polytrimethylene carbonate. Mice remained on the ventilator for ∼5 min after surgery, and were extubated at the initiation of spontaneous movement. Mice were recovered on a warm surface until ambulatory. Buprenorphine was administered as an analgesic twice daily (0.1 mg/kg SQ) for 48 hrs after PNX. Sham pneumonectomy animals underwent an identical procedure, except that after the thoracotomy, the chest was left open for 5 minutes to simulate the conditions of the pneumonectomy group without removal of the left lung, then closed as described.

### Histomorphometry and collagen content

Immediately following pulmonary function tests and while still under anesthesia, mice were euthanized by cervical dislocation, a tracheostomy performed, the chest opened, and the pulmonary circulation flushed via right ventricular puncture with PBS (10 mL). In the first study (**Fig. 1**), the degassed lung was filled with neutral buffered formalin (10%) to 25 cm H2O for 48–72 hrs. Once fixed, the lung lobes were oriented randomly in a cassette for embedding. The blocks were sectioned (5-µm-thick) such that all portions of the lung were represented on the slides. Slides were stained with hematoxylin and eosin and 15 randomly oriented non-overlapping fields (200× magnification) from two randomly selected sections (>100 microns apart) were photographed (Zeiss M1 Axioimager, Axiocam 1.0× digital camera, Carl Zeiss, Thornwood, NY). For measurement of MLI, a spaced non-continuous grid (of total grid length 1176 microns, 42 segments) was placed over each field oriented randomly. Images were taken of alveoli by excluding pleura, vessels, alveolar ducts (where obvious) and airways. Processing was performed using an automated macro written using commercial imaging software (SigmaScanPro, v.5.0, San Jose, CA) which permitted standardization of image thresholding, identification and recording of points of grid-image overlap. Mean linear intercept was defined as the total grid length (1176 microns) divided by the number of alveolar intersections.

Mean linear intercept (MLI) was defined as the total grid length (1176 microns) divided by the number of intersections. Total surface area was estimated on the basis of MLI and *in vivo* lung volume referred to as total lung capacity (TLC = IC+FRC) as follows: surface area = 4(TLC)/MLI.

In the second study (PNX vs SHAM, **Fig. 2**), the degassed lung was filled with neutral buffered formalin (10%) with low melt agar (1%) to 25 cm pressure for 72 hrs. Once fixed, lung volume was measure by water displacement according to Scherle [37]. Lung tissues were oriented randomly in cassettes and embedded in paraffin, followed by sectioning (5 uM) at two levels of random depth 100 uM apart. Slides (2 per section) were stained with hematoxylin and eosin and 30 randomly oriented non-overlapping fields (200× magnification) from each section were photographed, and MLI calculated as described above. Lung total surface area was computed on the basis of MLI and lung volume by water displacement as follows: surface area = 4VD/MLI, where VD was volume displacement of right lung and MLI mean linear intercept on same tissues. Additionally, adjacent sections were also stained with picrosirius and 20 fields (400×mag) per animal photographed. Digital images were converted to binary (black and white) images and intensity thresholded (SigmaScan Pro 5) for collagen staining for quantification of total pixel intensity for each image recorded. Values for 20 images per section (1 section/animal) were used statistical for comparison between groups.

### Cell proliferation and apoptosis

Formalin-fixed lungs were prepared for paraffin sectioning through standard methods. Antigen retrieval was performed by heating sections to 100°C in a citric acid buffer (Antigen Retrieval Solution; Vector Laboratories, Burlingame, CA) for 20 min and slowly cooling to room temperature. Sections were pretreated with hydrogen peroxide in methanol (3%, 15 min, 22°C) to quench endogenous peroxidases. Sections were blocked (20 min) with Dako Protein Block (X0909, Dako, Glostrup, Denmark) and incubated overnight (4°C) with the appropriate antibody diluted in Dako Antibody Diluent with Background Reducing Component (S3022, Dako, Glostrup, Denmark): monoclonal rat anti-mouse Ki-67 antigen (Clone Tec-3 M7249, Dako, Glostrup, Denmark), rat biotinylated IgG1 diluted 1∶100, rabbit polyclonal anti-prosurfactant protein C (proSP-C) (AB3786, Chemicon-Millipore, Billerica, MA) diluted 1∶1000. Anti-Ki-67 antibody was detected using a 1∶200 dilution of goat anti-rat IgG biotinylated secondary antibody (A10517, Invitrogen, Carlsbad, CA) and the Elite Vectastain ABC kit (PK-6100, Vector Laboratories, Burlingame, CA) for signal amplification. Visualization was achieved with Vector DAB (SK4100, Vector Laboratories, Burlingame, CA). After development of the DAB signal, for fluorescent immunostaining, donkey anti-rabbit secondary antibody (A21206, Invitrogen, Carlsbad, CA) diluted 1∶200 in PBS was applied and incubated at 37°C for 30 minutes.

To ensure specificity of immunostaining, adjacent tissue sections were stained with isotype antibodies (polyclonal rabbit serum for proSP-C and biotinylated rat IgG1 for Ki-67) plus secondary antibodies as described above. Tissues were examined to determine the percentage of nucleated cells comprising AECII (proSP-C+) cells per high-powered field (400×), as well as the percentage of AECII cells that were proliferating per high-powered field (400×), that is, positive for Ki-67 antigen on immunohistochemistry. Examination of the lung parenchyma was done by selecting non-overlapping fields initiated by random start and subsequent stage movement across parenchymal tissues in a random zig-zag pattern. Photomicrographs of 20 parenchymal regions were taken at a 400× magnification. All imaging and photography were performed using bright-field, FITC, and DAPI filters (Zeiss M1 Axioimager, Axiocam 1.0× digital camera, Carl Zeiss, Thornwood, NY).

Immunohistochemical detection of apoptosis was carried out using an In Situ Cell Death Detection Kit-TMR (Roche Molecular, Indianapolis, Indiana) following the procedures provided by the manufacturer. Briefly, rehydrated sections were permeabilized (Dako Target Retrieval Solution) for 5 minutes (99°C). Sections were blocked with 50–80 ul of 3% BSA/20% donkey serum in 0.1 M Tris-HCl (pH 7.5) for one hour (20C). A 1∶10 dilution of TdT enzyme in label solution was prepared, and then diluted 1∶5 in 1× PBS. Each tissue section was treated with 50–80 ul of this solution for 30 min in a humidified chamber at 20C. The negative control slide was treated with a 1∶5 solution of label solution in 1×PBS. Slides were then washed and sections incubated with rabbit anti-proSP-C antibody (AB3786, Chemicon, 1∶500) for 18 (4°C) and then with donkey anti-rabbit Alexafluor 488 (Invitrogen) for 30 (37°C). A control slide was made using a non-specific rabbit serum with the same secondary antibody. DAPI was applied for visualization of nuclei. The slides were analyzed under a fluorescent microscope. At least ten randomly chosen high-power fields (400*mag) were examined in each animal (n = 5/group). For each image, all DAPI-positive nuclei, all TUNEL-positive nuclei, and all pro-SPC positive cells were counted. The percentage of TUNEL-positive nuclei and the percentage of TUNEL-positive, pro-SPC positive cells were computed.

### Tissue preparation and RNA isolation and microarray analysis

The mice were anesthetized as above at 1 day, 3 days and 7 days after surgery and euthanized by cervical dislocation. The trachea cannulated, and the lungs removed *en bloc*. RNA preservation was achieved by flooding the lung intratracheally with RNAlater solution (Qiagen #76104), followed by storage of lung tissue samples in RNAlater at −80°C. The control samples used in this microarray experiment were the left lungs obtained from the study animals at the time of PNX and stored in RNAlater at −80°C. Total RNA was prepared from individual control and pnx samples using a combination of TRIzol reagent (Invitrogen #15596-018), and the Purelink RNA mini kit (Invitrogen #12183-018A) according to the manufacturer's directions. Total RNA concentrations, A260/A280 ratios and RIN values were determined using an Agilent 2100 Bioanalyzer (Aglient Technologies, Santa Clara CA). At each time point, equal amounts of total RNA from two individual samples were pooled (pairing the same samples for control and PNX), creating 3 pooled PNX samples and three pooled control samples (**Fig. 5**).

### Microarray data analysis on aged vs young pre- and post-PNX animals

Gene expression profiling experiments were carried out according to the Illumina BeadChip technology using the MouseWG-6v2 Expression BeadChip, permitting analysis of over 45,000 transcripts. Probe sets were background corrected and summarized using the RMA algorithm along with quantile normalization, all of which are available in BioConductor's “lumi” package [38]. Quality control on the arrays was performed by again using the lumi package to visualize intensity distributions, percentage present calls, and correlations between arrays; none of which turned up any reasons for concern and resulted in the inclusion of all arrays in the analysis. Following quality inspection, probe sets were filtered to include only those detected on all arrays. This step eliminated all probes with signal intensities reported at background level and resulted in the inclusion of roughly 17,500 probe sets for further analysis.

Data analysis of the filtered probe set can be described in two steps. First, differential gene expression was calculated between pneumonectomy and control samples for the animals within each age group and at each time point following surgery. This was accomplished using the “limma” (Linear Modeling for Microarray Data) package in BioConductor [39], with pneumonectomy and control sample pairings built into the model. Fold-change and p-values for each probe set were calculated using a moderated t-statistic, with the variance estimate being adjusted by incorporating global variation measures for the complete set of probes on the array. The P-value data were then corrected for multiple hypothesis testing using the Benjamini and Hochberg method [40]. This analysis resulted in unique sets of differentially expressed genes for each age group and time point. An absolute fold-change value ≥1.5 and adjusted P-value <0.05 were used as criteria for defining a set of genes for further exploration. Following completion of this analysis, second analysis addressed the question of how differential gene expression differed between the aged and young age groups.

To determine differences in differential gene expression between the aged and young animals, individual pair-wise fold-changes were calculated for each of the groups analyzed in step 1, providing threefold-change values per group (one for each paired replicate sample). This allowed for groups of fold-change values for a given time point in the aged group to be directly compared to those in the young group, again using the t-statistic. An absolute fold-change value ≥1.5 in at least one of the groups tested (i.e. either 3 month or 9 month groups) and adjusted P-value <0.05 were used as criteria for defining a set of genes for further exploration. Results of this analysis provided genes showing significant differential expression specific to the aged group compared to the young group with baseline differences subtracted (**Fig. 5**). Functional classification and pathway analysis for genes reported in both analyses was performed using Ingenuity Pathway Analysis. The complete microarray dataset is MIAME compliant and is available at Gene Expression Omnibus, a public functional genomics data repository supporting MIAME-compliant data submissions (accession number GSE27964 at: http://www.ncbi.nlm.nih.gov/geo/query/acc.cgi?acc=GSE27964).

### Ingenuity Pathways Analysis

Ingenuity Pathway Analysis (IPA) version 2.0 (Ingenuity® Systems Inc, Redwood City, CA; www.ingenuity.com) was used to search for biological functions and interrelationships between significantly modulated genes in PNX versus control mice. IPA provides a large manually curated database containing over 200,000 full text articles and information about thousands of human, mouse and rat genes [41] with which experimental data sets can be statistically compared. Genes from the dataset were overlaid onto a global molecular network developed from information contained within the IPA database, and networks of genes in the dataset were then algorithmically generated based on their connectivity (both direct and indirect relationships). Each network displays the type of relationship between two gene products, including genes that are not significantly altered in the user's microarray data set. The networks are ranked depending on the number of significantly expressed genes they contain, based on a *P*-value that indicates the likelihood of the genes in a network being found together due to chance. A score of 2 indicates a 1 in 100 chance that the focus genes of interest were linked in the network by chance rather than a direct biological relationship. Therefore, scores of 2 or higher have at least a 99% confidence level of not being generated by random chance alone [41].

### Quantitative reverse transcription PCR validation

Total RNA from the same pooled samples as used for the microarray analysis at the 1 day time point were used for qPCR analysis. First, the samples were subjected to genomic DNA elimination and first strand cDNA synthesis using a commercial kit (RT^2^ First Strand Kit, SA Biosciences) to generate the cDNA templates for PCR amplification. Quality control was performed using the SA Biosciences QC qRT-PCR array (SA Biosciences/Qiagen, Frederick, MD) to test for any inhibition of cDNA synthesis, or presence of genomic DNA contamination. Gene expression assays were performed using sets of premade mouse primer pairs (SA Biosciences) for Birc5, Cdk1a (p21), Col3A1, Eln, IL1beta, Lox, Retnla, Tgfbeta3, Tnc, Tnfsfr12a, and GusB (see Table 1). Quantitative PCR was performed using a ABI 7500 Detection system, and RT^2^ qPCR SYBR green PCR Master Mix (SA Biosciences), according to the manufacturer's recommended protocol. Each sample was analyzed in triplicate, and relative gene expression was calculated using the comparative Ct method [42] after normalization to the housekeeping gene GusB, which did not show differences in expression between aged and young mice (see online microarray dataset - accession number GSE27964 at: http://www.ncbi.nlm.nih.gov/geo/query/acc.cgi?acc=GSE27964).

### Immunohistochemistry

The mice were anesthetized as above at 0, 7 and 21 days after surgery, then euthanized by cervical dislocation. Following median sternotomy, the pulmonary vasculature was perfused with ice cold Hanks balanced salt solution, the trachea cannulated, and the lungs removed *en bloc*. Tissue fixation was achieved with intratracheal 10% buffered formalin at 25 cmH_2_0 overnight. The trachea was then ligated, and the lung was embedded in paraffin. Immunofluorescent staining (IF) was performed on 5 µm paraffin sections. Primary antibodies included the monoclonal mouse antibody anti-αSMA (Santa Cruz, dilution 1∶100). Tissue sections were deparaffinized and hydrated using standard methods, and antigen retrieval was performed using a citrate buffer (pH 6.0) and microwave heating (5 mins at high, 15 mins at 40% power). Tissues were washed (TBS with 0.1% Tween) three times before a 20 minute protein block (Dako, Carpinteria, CA), and then exposed to the primary antibodies (15–18 hours at 4 degrees Celsius). Detection of the primary antibodies was achieved using donkey anti-mouse Alexa Fluor 488 (green) at 1∶200 (30 mins at 37 degrees Celsius). The appropriate isotype control assays were also performed; non-specific staining was not observed. To examine the presence of αSMA positive parenchymal cells during lung regeneration, 20 randomly selected high power fields (400×) were photographed digitally for each time point (day 0, 7, 21). Cells were counted (averaging 50–100 nucleated cells/HPF) and the mean percentage of αSMA cells/nucleated cells was obtained. A two-way ANOVA and independent t-tests were performed to test for significance (*P*<0.05) between the three time points.

### Age effect on clonogenicity in isolated fibroblasts

The left lung removed at pneumonectomy in 3, 9, and 17 mo old mice was used for isolation of fibroblasts from outgrowth from explants. Briefly, lung tissues were minced and small (1 mm^2^) fragments were placed in shallow media (alpha-MEM, 15% FBS, 2 mM L-glutamine, penicillin, streptomycin, and amphotericin) in 6 well polystyrene plates. Media was added every other day for 10–12 days, at which time the cells were passaged on 150 cm^2^ dishes using trypsin free reagents (TrypLE Express, Invitrogen). Passages 3, 5, and 7 cells were plated onto 100 mm dishes (2000 cells/plate, n = 3 plates per passage) for enumeration of colonies of ≥50 cells (>5 doublings), and the colony forming efficiency (colonies/cells seeded)) compared between age groups at equivalent passages. For primary cell (passage 0) CFU-F the minced tissue was first digested with 2 mg/mL collagenase - dispase (Roche) for 60 min, serially filtered (100 uM and 40 uM), and red blood cells lysed (with subsequent cell washes in media) prior to plating (20,000 cells/plate).

### Tgfb1 and Tgfβ3 activation assays

Fibroblasts (passage 4) derived from explants from donors of different ages (3, 9, or 17 mo) were grown to 80–90% confluence and serum starved 6 hrs. Human recombinant TGFβ1 or Tgfβ3 (R&D Systems, 0 vs. 10 ng/mL) was added; cell (4 wells/condition) were harvested after 48 hrs and mRNA preserved in RNA Protect (−80°C) for qPCR (see Quantitative PCR). RNA isolation and qPCR analysis was performed as described above using commercial primers (SABiosciences) listed in Table 2.

### Immunocytochemistry

Passage 4, 6 and 7 cells were cultured on polystyrene plates in basal media (alpha MEM, 15% FBS) to 80% confluence. After fixation with methanol (−20°C, 10 .minutes), the plates were stored at 4°C until immunostaining. Primary antibodies included the monoclonal mouse antibody anti-αSMA (Santa Cruz, dilution 1∶100), and the polyclonal rabbit antibodies anti-S100A4 (Santa Cruz, dilution 1∶100) and Col3A1 (Santa Cruz, dilution 1∶100). The plates were washed three times in TBS before a 20 minute protein block (Dako, Carpinteria, CA), and then exposed to the primary antibodies (1–2 hours at room temperature). Detection of the primary antibodies was achieved using donkey anti-mouse Alexa Fluor 594 (red) at 1∶200 (30 mins at 37°C). The appropriate isotype control assays were also performed; non-specific staining was not observed.

### Flow cytometry

Passage 4 lung fibroblasts derived from 3, 9 and 17 month mice were removed from culture plates (500,000 cells) and analyzed in triplicate using flow cytometry gated on forward and side scatter and analyzed for viability using 7AAD (30,000 independent events). Relative cell size was determined using mean forward scatter.

### Statistical analysis

Pulmonary function, mean linear intercept data, cell counts, cellular proliferation, apoptosis, and densitometric assays were analyzed using a two way ANOVA (group, time, group*time) and *post-hoc* independent T tests, with the level of significance set at *P*<0.05. All statistical analysis was performed using commercial software (SPSS v. 13.0, SPSS Inc, Chicago, IL). Data is presented as mean + SD. Pairwise comparisons between different groups were made using Student's T test assuming unequal variance. Quantitative PCR was analyzed using webtools (SABioscience) by published methods [42]. Otherwise, data were analyzed using commercial software (Excel SP3, Microsoft, Redmond, WA). Data is presented as mean+SEM unless otherwise indicated. A *P* value <0.05 was considered statistically significant.

## Supporting Information

Table S1 Microarray analysis of genes that are differentially regulated (fold change) in 9 month vs 3 month mice without PNX (control) (P<0.05).
(DOC)

Table S2 Microarray analysis of genes with significant differences (P<0.05) in differential regulation (fold change, compared to pre-PNX samples) between 9 and 3 month mice 1 day after PNX.
(DOC)

Table S3 Microarray analysis of genes with significant differences (P<0.05) in differential regulation (fold change, compared to pre-PNX samples) between 9 and 3 month mice 3 days after PNX.
(DOC)

Table S4 Microarray analysis of genes with significant differences (P<0.05) in differential regulation (fold change, compared to pre-PNX samples) between 9 and 3 month mice 7 days after PNX.
(DOC)
