# Supplementary material for: Age-Dependent Decline in Mouse Lung Regeneration with Loss of Lung Fibroblast Clonogenicity and Increased Myofibroblastic Differentiation
Source: PLoS One. 2011 Aug 30;6(8):e23232. doi: 10.1371/journal.pone.0023232 (PMC3166052; doi:10.1371/journal.pone.0023232)
Supplement: Table S1 — Microarray analysis of genes that are differentially regulated (fold change) in 9 month vs 3 month mice without PNX (control) (P<0.05). (DOC) [file pone.0023232.s001.doc]

**Table S1** – Microarray analysis of genes that are differentially regulated (fold change) in 9 month vs 3 month mice without PNX (control) (P<0.05)

| SYMBOL | FC | P | PROBE_ID |
| --- | --- | --- | --- |
| 0610007N19Rik | -1.2 | 0.03 | ILMN_2543688 |
| 0610007N19Rik | -1.3 | 0.01 | ILMN_1233188 |
| 1110017D15Rik | 1.2 | 0.00 | ILMN_3043178 |
| 1110017D15Rik | 1.3 | 0.00 | ILMN_3118081 |
| 1110017D15Rik | 1.3 | 0.00 | ILMN_2617920 |
| 1110049B09Rik | 1.6 | 0.00 | ILMN_3159695 |
| 1110049B09Rik | 1.3 | 0.00 | ILMN_2616045 |
| 1190002N15Rik | -1.3 | 0.02 | ILMN_3162133 |
| 1190002N15Rik | -1.2 | 0.00 | ILMN_2524691 |
| 1300007L22Rik | 1.3 | 0.00 | ILMN_2632839 |
| 1300013J15Rik | 1.3 | 0.00 | ILMN_2849016 |
| 1600029I14Rik | 1.3 | 0.00 | ILMN_2691743 |
| 1700001C02Rik | 1.4 | 0.00 | ILMN_3003501 |
| 1700003E16Rik | 1.2 | 0.01 | ILMN_1231608 |
| 1700003M02Rik | 1.2 | 0.00 | ILMN_2594224 |
| 1700007G11Rik | 1.3 | 0.00 | ILMN_3003018 |
| 1700010A17Rik | 1.2 | 0.04 | ILMN_2712454 |
| 1700016K19Rik | 1.3 | 0.01 | ILMN_1250938 |
| 1700019L03Rik | 1.3 | 0.00 | ILMN_1256787 |
| 1700021K14Rik | 1.2 | 0.04 | ILMN_1224436 |
| 1700024G13Rik | 1.4 | 0.01 | ILMN_2711761 |
| 1700026D08Rik | 1.4 | 0.00 | ILMN_2670708 |
| 1700026L06Rik | 1.2 | 0.01 | ILMN_2720572 |
| 1700027A23Rik | 1.3 | 0.00 | ILMN_2736521 |
| 1700027N10Rik | 1.3 | 0.01 | ILMN_2775220 |
| 1700045I19Rik | 1.2 | 0.01 | ILMN_2967255 |
| 1700088E04Rik | 1.4 | 0.00 | ILMN_1239196 |
| 1700120B06Rik | 1.3 | 0.01 | ILMN_3163424 |
| 1700129I04Rik | 1.2 | 0.02 | ILMN_1224390 |
| 1810011O10Rik | -1.2 | 0.02 | ILMN_1254307 |
| 1810013B01Rik | 1.2 | 0.03 | ILMN_2774244 |
| 2010001M09Rik | 1.6 | 0.00 | ILMN_2613601 |
| 2010111I01Rik | -1.3 | 0.01 | ILMN_2838372 |
| 2210018M11Rik | -1.1 | 0.04 | ILMN_2521686 |
| 2310003M01Rik | 1.2 | 0.04 | ILMN_1214024 |
| 2310007L24Rik | 1.2 | 0.03 | ILMN_2596403 |
| 2310014D11Rik | -1.2 | 0.04 | ILMN_2423372 |
| 2310016C16Rik | -1.3 | 0.03 | ILMN_2653166 |
| 2310040A07Rik | -1.2 | 0.02 | ILMN_2479977 |
| 2410076I21Rik | 1.3 | 0.00 | ILMN_2694782 |
| 2410116G06Rik | 1.2 | 0.03 | ILMN_2980015 |
| 2610203C22Rik | -1.4 | 0.00 | ILMN_2461487 |
| 2700063P19Rik | -1.5 | 0.00 | ILMN_2546861 |
| 2810022L02Rik | -1.2 | 0.02 | ILMN_2645865 |
| 2810484G07Rik | 2.3 | 0.00 | ILMN_2467007 |
| 2900062L11Rik | -1.3 | 0.00 | ILMN_2609182 |
| 3100002J23Rik | 1.3 | 0.00 | ILMN_2635708 |
| 3110032G18Rik | -1.4 | 0.00 | ILMN_1244695 |
| 3300002A11Rik | 1.3 | 0.00 | ILMN_2440486 |
| 3930401E15Rik | -1.2 | 0.03 | ILMN_2579611 |
| 4732429D16Rik | 1.3 | 0.02 | ILMN_1230287 |
| 4922501L14Rik | 1.4 | 0.00 | ILMN_2606152 |
| 4930455F23Rik | 1.2 | 0.03 | ILMN_2769153 |
| 4930455F23Rik | 1.3 | 0.00 | ILMN_2908953 |
| 4930463G05Rik | 1.3 | 0.02 | ILMN_2691041 |
| 4930502E18Rik | 1.2 | 0.01 | ILMN_1222772 |
| 4930535E21Rik | 1.5 | 0.00 | ILMN_2881312 |
| 4930579J09Rik | 1.2 | 0.04 | ILMN_2771429 |
| 4932408C11Rik | 1.2 | 0.00 | ILMN_1214466 |
| 4932425I24Rik | 1.3 | 0.00 | ILMN_2679908 |
| 4932443I19Rik | 1.3 | 0.01 | ILMN_2627818 |
| 4933428D01Rik | 1.3 | 0.00 | ILMN_2707902 |
| 4933439C20Rik | 1.5 | 0.00 | ILMN_2944601 |
| 5133400G04Rik | 1.3 | 0.02 | ILMN_3030392 |
| 5730469M10Rik | 1.4 | 0.00 | ILMN_1221501 |
| 5830467P10Rik | 1.5 | 0.00 | ILMN_1234099 |
| 6030429G01Rik | 1.2 | 0.02 | ILMN_2959253 |
| 6130401L20Rik | -1.2 | 0.01 | ILMN_2728202 |
| 6330405H19 | 1.3 | 0.00 | ILMN_2731908 |
| 6430514L14Rik | 1.2 | 0.00 | ILMN_2733204 |
| 6430537H07Rik | 1.3 | 0.01 | ILMN_3007065 |
| 6430537H07Rik | 1.3 | 0.00 | ILMN_2720911 |
| 6820408C15Rik | 1.3 | 0.02 | ILMN_1245721 |
| 9030205A07Rik | 1.7 | 0.00 | ILMN_1222844 |
| 9130218O11Rik | 1.4 | 0.00 | ILMN_2756223 |
| 9130230L23Rik | 1.2 | 0.03 | ILMN_1218715 |
| 9130230L23Rik | 1.2 | 0.01 | ILMN_2625233 |
| 9430052C07Rik | -2.2 | 0.00 | ILMN_2506727 |
| 9430073N08Rik | -1.4 | 0.00 | ILMN_1226398 |
| 9530051K01Rik | 1.3 | 0.01 | ILMN_2673332 |
| A130082N24Rik | -1.2 | 0.03 | ILMN_1254869 |
| A330102K04Rik | 2.2 | 0.00 | ILMN_1252157 |
| Abhd5 | -1.2 | 0.00 | ILMN_2739295 |
| Abi3bp | -1.4 | 0.00 | ILMN_3132588 |
| Abp1 | 1.5 | 0.00 | ILMN_2601519 |
| Acacb | 1.2 | 0.02 | ILMN_2421890 |
| Acot7 | -1.2 | 0.03 | ILMN_2632264 |
| Acot7 | -1.2 | 0.02 | ILMN_2807084 |
| Acot7 | -1.3 | 0.01 | ILMN_2606180 |
| Acpl2 | 1.2 | 0.02 | ILMN_1254634 |
| Adamts12 | -1.4 | 0.00 | ILMN_1234413 |
| Adamts2 | -1.5 | 0.03 | ILMN_1221851 |
| Adamts2 | -1.6 | 0.00 | ILMN_1226259 |
| Adamts2 | -1.7 | 0.00 | ILMN_2729103 |
| Adamts9 | -1.7 | 0.00 | ILMN_1257724 |
| Adh1 | -1.3 | 0.01 | ILMN_1258501 |
| Adora1 | 1.3 | 0.01 | ILMN_1254016 |
| Aebp1 | 1.2 | 0.04 | ILMN_2873822 |
| Aggf1 | -1.1 | 0.04 | ILMN_2858324 |
| Agrp | 1.8 | 0.00 | ILMN_2703427 |
| Agtrl1 | -1.9 | 0.00 | ILMN_2748850 |
| Ahnak2 | 1.3 | 0.00 | ILMN_3161105 |
| AI324046 | 3.1 | 0.00 | ILMN_2743883 |
| AI324046 | 3.2 | 0.00 | ILMN_2743884 |
| AI324046 | 9.7 | 0.00 | ILMN_2703329 |
| AI324046 | 14.1 | 0.00 | ILMN_2523841 |
| Aim1l | 1.2 | 0.01 | ILMN_1214944 |
| Akap14 | 1.3 | 0.00 | ILMN_2780362 |
| Akr1b7 | 1.3 | 0.00 | ILMN_1238042 |
| Akr1b8 | 1.3 | 0.02 | ILMN_1219188 |
| Aldh3b1 | 1.2 | 0.00 | ILMN_2645793 |
| Alg14 | -1.2 | 0.03 | ILMN_2605575 |
| Alox12 | 2.0 | 0.00 | ILMN_2613908 |
| Ankrd1 | 1.5 | 0.02 | ILMN_2950286 |
| Anln | -1.2 | 0.03 | ILMN_2595597 |
| Antxr1 | -1.2 | 0.03 | ILMN_1226183 |
| Antxr2 | -1.2 | 0.04 | ILMN_1214412 |
| Ap3m2 | 1.2 | 0.03 | ILMN_2912598 |
| Apol9b | 1.3 | 0.00 | ILMN_2622856 |
| Aqp9 | 1.3 | 0.04 | ILMN_1214634 |
| Arhgdig | 1.5 | 0.00 | ILMN_1240164 |
| Arhgdig | 1.3 | 0.00 | ILMN_2950622 |
| Arl13b | -1.2 | 0.04 | ILMN_2426195 |
| Aspa | 1.2 | 0.02 | ILMN_1214573 |
| Atf5 | 1.3 | 0.04 | ILMN_2646985 |
| AU019823 | -1.3 | 0.00 | ILMN_2875915 |
| AU021034 | 1.3 | 0.00 | ILMN_2914305 |
| AU023871 | 1.7 | 0.00 | ILMN_3161372 |
| AV249152 | 1.2 | 0.04 | ILMN_2673790 |
| AV249152 | 1.3 | 0.00 | ILMN_2970672 |
| Azgp1 | 1.4 | 0.00 | ILMN_2725198 |
| Azgp1 | 1.9 | 0.00 | ILMN_2837080 |
| B230365C01Rik | -1.4 | 0.00 | ILMN_1252723 |
| B230373P09Rik | 1.4 | 0.00 | ILMN_2671128 |
| B230386D16Rik | -1.2 | 0.02 | ILMN_2565729 |
| B430216N15Rik | -1.3 | 0.00 | ILMN_1255967 |
| B930041F14Rik | 1.3 | 0.00 | ILMN_1250201 |
| B930045J24Rik | 1.2 | 0.04 | ILMN_2567349 |
| Bbox1 | 1.7 | 0.00 | ILMN_2686029 |
| BC022687 | 1.2 | 0.00 | ILMN_2631663 |
| BC024997 | 1.2 | 0.00 | ILMN_1247389 |
| BC038167 | 1.3 | 0.00 | ILMN_2450117 |
| BC038167 | 1.3 | 0.00 | ILMN_1223757 |
| BC044804 | 1.3 | 0.00 | ILMN_2475376 |
| BC048546 | 1.5 | 0.00 | ILMN_2984219 |
| BC048546 | 1.5 | 0.00 | ILMN_2531773 |
| BC050196 | 1.3 | 0.00 | ILMN_1227083 |
| Bcas1 | 1.2 | 0.00 | ILMN_2662264 |
| Bdh2 | -1.2 | 0.02 | ILMN_2641678 |
| Best1 | 1.2 | 0.00 | ILMN_1212602 |
| Bex2 | -1.4 | 0.01 | ILMN_3065164 |
| Bex4 | -1.3 | 0.03 | ILMN_3043587 |
| Bex4 | -1.4 | 0.00 | ILMN_3118584 |
| Bzrap1 | 1.4 | 0.00 | ILMN_1229256 |
| Bzw2 | -1.1 | 0.04 | ILMN_2876482 |
| C130026I21Rik | 1.3 | 0.01 | ILMN_3162925 |
| C130026I21Rik | 1.3 | 0.00 | ILMN_1234720 |
| C130092E12 | -1.2 | 0.03 | ILMN_2448997 |
| C1ql2 | 1.4 | 0.00 | ILMN_2671473 |
| C3 | 1.2 | 0.04 | ILMN_2759484 |
| C330001K17Rik | 1.3 | 0.00 | ILMN_2987394 |
| C330001K17Rik | 1.2 | 0.00 | ILMN_1214455 |
| C4a | 1.2 | 0.01 | ILMN_1215092 |
| C7 | -1.3 | 0.01 | ILMN_2536590 |
| C76566 | -1.2 | 0.03 | ILMN_2608622 |
| C85492 | 1.2 | 0.01 | ILMN_2721748 |
| C920004C08Rik | 1.3 | 0.01 | ILMN_1222071 |
| Cacna1h | 1.4 | 0.00 | ILMN_1246201 |
| Calml4 | 1.3 | 0.00 | ILMN_1246573 |
| Calu | -1.2 | 0.01 | ILMN_2771956 |
| Capn6 | -1.4 | 0.00 | ILMN_2695143 |
| Car14 | 1.2 | 0.04 | ILMN_1220686 |
| Car14 | 1.4 | 0.00 | ILMN_2973824 |
| Casc1 | 1.2 | 0.01 | ILMN_2906552 |
| Cask | -1.2 | 0.03 | ILMN_1212894 |
| Casp1 | 1.4 | 0.00 | ILMN_1247592 |
| Casp4 | 1.2 | 0.04 | ILMN_2811737 |
| Ccdc108 | 1.4 | 0.00 | ILMN_3162084 |
| Ccdc113 | 1.4 | 0.00 | ILMN_2610277 |
| Ccdc114 | 1.2 | 0.02 | ILMN_1231387 |
| Ccdc19 | 1.3 | 0.01 | ILMN_2648180 |
| Ccdc65 | 1.2 | 0.01 | ILMN_2595469 |
| Ccdc65 | 1.3 | 0.01 | ILMN_1253268 |
| Ccdc80 | -1.5 | 0.00 | ILMN_1234824 |
| Ccl25 | 1.2 | 0.03 | ILMN_2942353 |
| Ccl9 | 1.4 | 0.04 | ILMN_2776603 |
| Ccno | -1.4 | 0.00 | ILMN_2736471 |
| Ccr6 | 1.4 | 0.00 | ILMN_2888448 |
| Ccr6 | 1.2 | 0.00 | ILMN_2639012 |
| Cd177 | 1.2 | 0.02 | ILMN_1225233 |
| Cd177 | 2.1 | 0.00 | ILMN_2789900 |
| Cd207 | -1.2 | 0.00 | ILMN_2785648 |
| Cd84 | 1.2 | 0.00 | ILMN_2754698 |
| Cdc2l6 | 1.2 | 0.02 | ILMN_2644664 |
| Cdh16 | -1.5 | 0.00 | ILMN_2670038 |
| Cdh26 | 1.2 | 0.01 | ILMN_2707976 |
| Chek2 | 1.2 | 0.01 | ILMN_2633492 |
| Chia | 1.4 | 0.01 | ILMN_2955937 |
| Chia | 1.4 | 0.01 | ILMN_2955940 |
| Chrna10 | 1.2 | 0.02 | ILMN_3073524 |
| Chst2 | -1.3 | 0.00 | ILMN_2687165 |
| Cidea | 1.3 | 0.01 | ILMN_1215446 |
| Ckmt1 | 1.3 | 0.02 | ILMN_2773537 |
| Cldn10 | -1.3 | 0.03 | ILMN_2723576 |
| Clec4a1 | 1.3 | 0.04 | ILMN_1230708 |
| Clec4d | 1.5 | 0.01 | ILMN_2705628 |
| Clec4g | 1.3 | 0.01 | ILMN_1234165 |
| Clic6 | 1.2 | 0.00 | ILMN_2667635 |
| Clstn3 | 1.2 | 0.02 | ILMN_2827217 |
| Col17a1 | -1.3 | 0.01 | ILMN_1240481 |
| Col1a1 | -2.2 | 0.00 | ILMN_2687872 |
| Col1a2 | -1.5 | 0.03 | ILMN_1253806 |
| Col3a1 | -2.2 | 0.01 | ILMN_1258629 |
| Col4a1 | -1.4 | 0.00 | ILMN_2621643 |
| Col4a2 | -1.4 | 0.00 | ILMN_2822579 |
| Col4a3 | -1.5 | 0.00 | ILMN_1213850 |
| Col4a4 | -1.3 | 0.03 | ILMN_2983387 |
| Col5a1 | -1.5 | 0.00 | ILMN_2748402 |
| Col5a2 | -1.3 | 0.02 | ILMN_2470069 |
| Col6a1 | -1.4 | 0.00 | ILMN_1259388 |
| Col6a1 | -1.5 | 0.00 | ILMN_2768087 |
| Col6a2 | -1.2 | 0.01 | ILMN_1258759 |
| Col6a2 | -1.5 | 0.00 | ILMN_1216661 |
| Col6a3 | -1.4 | 0.01 | ILMN_1249220 |
| Copg2as2 | -1.4 | 0.00 | ILMN_2549929 |
| Crispld2 | -1.2 | 0.02 | ILMN_1257551 |
| Csf3r | 1.4 | 0.03 | ILMN_1255648 |
| Csf3r | 1.4 | 0.01 | ILMN_2806180 |
| Csprs | 1.5 | 0.00 | ILMN_2661289 |
| Csrp3 | 1.3 | 0.01 | ILMN_2742068 |
| Ctla4 | 1.2 | 0.03 | ILMN_2631752 |
| Ctse | 1.4 | 0.00 | ILMN_2960325 |
| Cxcl13 | 1.7 | 0.00 | ILMN_2760019 |
| Cxcl14 | -1.2 | 0.03 | ILMN_2659426 |
| Cxcl14 | -1.4 | 0.00 | ILMN_2760800 |
| Cxcl4 | 1.4 | 0.02 | ILMN_2757966 |
| Cxcr3 | 1.4 | 0.00 | ILMN_2658786 |
| Cxcr4 | -1.3 | 0.00 | ILMN_2630459 |
| Cyp2s1 | 1.2 | 0.00 | ILMN_2758264 |
| Cyp4f39 | 1.3 | 0.00 | ILMN_1221376 |
| D0H4S114 | -1.7 | 0.00 | ILMN_2680054 |
| D130043K22Rik | 1.2 | 0.01 | ILMN_1218920 |
| D130084M03Rik | 1.2 | 0.00 | ILMN_2680371 |
| D14Ertd449e | -1.2 | 0.01 | ILMN_2693858 |
| D1Ertd471e | 1.3 | 0.00 | ILMN_2440679 |
| D330014H01Rik | 1.4 | 0.00 | ILMN_2743932 |
| D4Bwg1540e | 1.3 | 0.01 | ILMN_2660409 |
| D6Mit97 | 1.9 | 0.00 | ILMN_2467429 |
| D830044I16Rik | 1.3 | 0.00 | ILMN_1248156 |
| D930046M13Rik | -1.2 | 0.03 | ILMN_1224607 |
| D9Ertd280e | 1.3 | 0.03 | ILMN_1231836 |
| Darc | 1.2 | 0.04 | ILMN_2656748 |
| Dbndd2 | -1.2 | 0.04 | ILMN_3026137 |
| Dcbld1 | -1.2 | 0.01 | ILMN_1253155 |
| Dcun1d3 | -1.2 | 0.01 | ILMN_2901402 |
| Ddah1 | 1.4 | 0.00 | ILMN_1256676 |
| Ddc | -1.4 | 0.01 | ILMN_2628647 |
| Ddc | -1.8 | 0.00 | ILMN_1260450 |
| Ddr2 | -1.2 | 0.01 | ILMN_1230373 |
| Ddx26 | 1.2 | 0.04 | ILMN_2576590 |
| Diras2 | 1.3 | 0.00 | ILMN_2926480 |
| Dkkl1 | 1.2 | 0.04 | ILMN_2752580 |
| Dmkn | 1.2 | 0.03 | ILMN_1229763 |
| Dmkn | 1.2 | 0.00 | ILMN_3105563 |
| Dnahc2 | 1.2 | 0.02 | ILMN_3123043 |
| Dnahc9 | 1.3 | 0.00 | ILMN_2630138 |
| Dnaic1 | 1.2 | 0.00 | ILMN_2744879 |
| Dnali1 | 1.3 | 0.01 | ILMN_2872896 |
| Dpysl3 | -1.4 | 0.00 | ILMN_1250075 |
| Dsg2 | 1.2 | 0.00 | ILMN_1221153 |
| Dync2h1 | 1.2 | 0.02 | ILMN_1238984 |
| Dynlrb2 | 1.3 | 0.02 | ILMN_1215019 |
| E030019B06Rik | 1.3 | 0.02 | ILMN_2751796 |
| E030030K01Rik | -1.6 | 0.00 | ILMN_2582952 |
| E230008N13Rik | 1.3 | 0.00 | ILMN_1228909 |
| E230024B12Rik | -1.2 | 0.00 | ILMN_1253191 |
| E430002D04Rik | 1.4 | 0.00 | ILMN_2629663 |
| Ebpl | 1.3 | 0.00 | ILMN_1250555 |
| Edem2 | 1.2 | 0.03 | ILMN_2684279 |
| Edn1 | 1.5 | 0.00 | ILMN_2737713 |
| Ednrb | -1.3 | 0.01 | ILMN_2589640 |
| Efcab1 | 1.3 | 0.00 | ILMN_2917280 |
| Efcab3 | 1.3 | 0.00 | ILMN_2928498 |
| Efhd1 | 1.3 | 0.00 | ILMN_2613306 |
| EG245190 | 1.3 | 0.02 | ILMN_2472741 |
| EG433016 | 1.4 | 0.03 | ILMN_2995688 |
| EG434197 | 1.2 | 0.04 | ILMN_3014674 |
| EG638695 | 1.9 | 0.02 | ILMN_2809443 |
| Emilin2 | 1.3 | 0.02 | ILMN_2981363 |
| Emp1 | -1.2 | 0.00 | ILMN_2642913 |
| Enpp4 | 1.2 | 0.00 | ILMN_2762380 |
| Entpd4 | 1.2 | 0.01 | ILMN_2554110 |
| Entpd5 | 1.2 | 0.02 | ILMN_1215096 |
| Epn2 | 1.2 | 0.02 | ILMN_2659617 |
| Eraf | 1.5 | 0.01 | ILMN_2619200 |
| Esm1 | -1.6 | 0.00 | ILMN_1257574 |
| Espn | 1.3 | 0.00 | ILMN_2731578 |
| Espn | 1.3 | 0.00 | ILMN_3107059 |
| F13a1 | 1.2 | 0.01 | ILMN_2914938 |
| F430201B04Rik | 1.2 | 0.04 | ILMN_2902336 |
| F5 | 1.4 | 0.00 | ILMN_1248713 |
| Fabp3 | 1.4 | 0.02 | ILMN_2887630 |
| Fank1 | 1.2 | 0.04 | ILMN_2977849 |
| Fbln1 | -1.3 | 0.02 | ILMN_2728985 |
| Fbln1 | -1.3 | 0.00 | ILMN_2870672 |
| Fbln2 | -1.3 | 0.01 | ILMN_1245307 |
| Fbn1 | -1.7 | 0.00 | ILMN_1223552 |
| Fcgr4 | 1.5 | 0.00 | ILMN_2631161 |
| Fcrls | -1.4 | 0.02 | ILMN_2770968 |
| Fga | 1.2 | 0.01 | ILMN_2624363 |
| Fgf1 | -1.2 | 0.03 | ILMN_2701233 |
| Fgf10 | -1.3 | 0.04 | ILMN_2982771 |
| Fgfbp3 | -1.4 | 0.00 | ILMN_2841593 |
| Fgg | 1.2 | 0.00 | ILMN_2832105 |
| Fhit | 1.2 | 0.01 | ILMN_2748680 |
| Foxc1 | 1.2 | 0.04 | ILMN_2886260 |
| Frrs1 | -1.3 | 0.00 | ILMN_3098120 |
| Fstl1 | -1.3 | 0.02 | ILMN_2734683 |
| Fstl4 | -1.2 | 0.04 | ILMN_2655058 |
| Fxyd2 | 1.1 | 0.02 | ILMN_2616328 |
| Fxyd3 | -1.6 | 0.00 | ILMN_2595593 |
| Fxyd4 | 1.3 | 0.01 | ILMN_2670751 |
| Fxyd6 | 1.3 | 0.00 | ILMN_2609998 |
| Fyb | 1.2 | 0.01 | ILMN_1236105 |
| Fzd2 | -1.2 | 0.04 | ILMN_2939666 |
| Gal | -1.4 | 0.02 | ILMN_2776034 |
| Gata3 | 1.2 | 0.02 | ILMN_1248843 |
| Ggcx | -1.2 | 0.00 | ILMN_1232184 |
| Gli2 | -1.2 | 0.01 | ILMN_2522863 |
| Gls2 | 1.2 | 0.04 | ILMN_2840975 |
| Gm1574 | 1.3 | 0.00 | ILMN_1260237 |
| Gm1673 | 1.3 | 0.00 | ILMN_2802311 |
| Gm1964 | 1.2 | 0.00 | ILMN_3161168 |
| Gm973 | 1.2 | 0.02 | ILMN_1229313 |
| Gnaz | 1.3 | 0.00 | ILMN_3161289 |
| Got1l1 | 1.4 | 0.00 | ILMN_1246289 |
| Gp1bb | 1.6 | 0.00 | ILMN_2653205 |
| Gp2 | 1.3 | 0.04 | ILMN_2904435 |
| Gp49a | 1.4 | 0.01 | ILMN_1248139 |
| Gp5 | 1.8 | 0.00 | ILMN_1236762 |
| Gp6 | 1.2 | 0.00 | ILMN_2640560 |
| Gp9 | 1.7 | 0.00 | ILMN_2842601 |
| Gpc1 | 1.2 | 0.01 | ILMN_2635784 |
| Gpc3 | -1.2 | 0.01 | ILMN_2719973 |
| Gpr114 | 1.2 | 0.00 | ILMN_2856926 |
| Gpr23 | -1.3 | 0.00 | ILMN_1242617 |
| Gprc6a | -1.3 | 0.01 | ILMN_2588955 |
| Gpx7 | -1.2 | 0.01 | ILMN_2724294 |
| Gria1 | -1.2 | 0.04 | ILMN_1221295 |
| Grp | -1.3 | 0.00 | ILMN_2671608 |
| Gsn | -1.6 | 0.03 | ILMN_2679386 |
| Gucy1a3 | -1.3 | 0.03 | ILMN_1228564 |
| Gucy1a3 | -1.4 | 0.01 | ILMN_1241211 |
| H2-Ab1 | 1.2 | 0.04 | ILMN_1226525 |
| H2-DMb1 | 1.2 | 0.00 | ILMN_1244977 |
| H2-DMb2 | 1.3 | 0.04 | ILMN_2589164 |
| H2-M2 | 2.1 | 0.00 | ILMN_2964185 |
| Hap1 | 1.2 | 0.02 | ILMN_1244829 |
| Hbp1 | -1.2 | 0.00 | ILMN_2773012 |
| Heph | -1.3 | 0.00 | ILMN_2726128 |
| Heph | -1.4 | 0.00 | ILMN_2734252 |
| Hhatl | 1.2 | 0.02 | ILMN_2707207 |
| Hhip | -1.2 | 0.03 | ILMN_2684250 |
| Hist1h1c | 1.3 | 0.04 | ILMN_2855315 |
| Hist1h2ac | 1.2 | 0.03 | ILMN_2666418 |
| Hist1h4i | 1.2 | 0.00 | ILMN_1232524 |
| Hmgcs2 | 1.6 | 0.00 | ILMN_1216322 |
| Homer2 | -1.1 | 0.03 | ILMN_2588474 |
| Hpcal4 | 1.4 | 0.01 | ILMN_2662054 |
| Hspa1l | 1.2 | 0.03 | ILMN_2654682 |
| Hspa2 | 1.2 | 0.04 | ILMN_1223285 |
| Hspa2 | 1.3 | 0.00 | ILMN_3140383 |
| Htr2b | 1.2 | 0.00 | ILMN_2589768 |
| Ide | -1.3 | 0.02 | ILMN_1243499 |
| Ier3 | 1.3 | 0.02 | ILMN_1216764 |
| Ifitm6 | 1.5 | 0.01 | ILMN_1218181 |
| Ift172 | 1.2 | 0.04 | ILMN_2611450 |
| Ift81 | 1.2 | 0.00 | ILMN_2735422 |
| Igf1 | -1.2 | 0.01 | ILMN_2769884 |
| Igf2 | 1.3 | 0.00 | ILMN_2597769 |
| Igf2bp3 | -1.2 | 0.04 | ILMN_1218913 |
| Igfbp2 | 1.7 | 0.00 | ILMN_1236788 |
| Igfbp2 | 1.6 | 0.00 | ILMN_2930897 |
| Igh-4 | 1.5 | 0.01 | ILMN_2515956 |
| Igh-6 | 5.6 | 0.00 | ILMN_2744660 |
| Igh-6 | 5.8 | 0.00 | ILMN_2744657 |
| Ighg | 15.5 | 0.00 | ILMN_1249975 |
| IGHV12S1_M22439_Ig_heavy_variable_12S1_339 | 1.4 | 0.00 | ILMN_2433110 |
| IGHV1S119_L33961_Ig_heavy_variable_1S119_14 | 2.0 | 0.00 | ILMN_2512738 |
| IGHV1S120_AF025443_Ig_heavy_variable_1S120_8 | 3.2 | 0.00 | ILMN_2470564 |
| IGHV1S124_AF025449_Ig_heavy_variable_1S124_11 | 1.6 | 0.00 | ILMN_2422974 |
| IGHV1S28_X02460_Ig_heavy_variable_1S28_13 | 1.4 | 0.00 | ILMN_2517595 |
| IGHV1S31_X02463_Ig_heavy_variable_1S31_40 | 1.2 | 0.02 | ILMN_1224026 |
| IGHV1S35_M12376_Ig_heavy_variable_1S35_13 | 3.8 | 0.00 | ILMN_2432262 |
| IGHV1S36_M13788_Ig_heavy_variable_1S36_40 | 2.7 | 0.00 | ILMN_2437019 |
| IGHV8S7_U23022_Ig_heavy_variable_8S7_163 | 1.5 | 0.00 | ILMN_2457614 |
| Igh-VJ558 | 5.4 | 0.00 | ILMN_2772264 |
| Igh-VJ558 | 6.6 | 0.00 | ILMN_2635272 |
| Igk-C | 2.9 | 0.00 | ILMN_2684370 |
| IGKV2-137_AJ231263_Ig_kappa_variable_2-137_15 | 1.8 | 0.00 | ILMN_2499056 |
| IGKV3-2_X16954_Ig_kappa_variable_3-2_18 | 21.8 | 0.00 | ILMN_1251011 |
| Igk-V33 | 1.4 | 0.02 | ILMN_1248308 |
| Igk-V38 | 2.1 | 0.00 | ILMN_1254159 |
| Igk-V38 | 2.1 | 0.00 | ILMN_1238932 |
| IGKV4-71_AJ231218_Ig_kappa_variable_4-71_20 | 3.1 | 0.00 | ILMN_2513393 |
| IGKV4-73_AJ231216_Ig_kappa_variable_4-73_18 | 3.0 | 0.00 | ILMN_1242258 |
| IGKV4-80_AJ231213_Ig_kappa_variable_4-80_91 | 2.1 | 0.00 | ILMN_2462623 |
| Igk-V5 | 2.1 | 0.00 | ILMN_2631259 |
| Igk-V5 | 3.3 | 0.00 | ILMN_1228607 |
| IGKV8-31_AJ235957_Ig_kappa_variable_8-31_3 | 1.9 | 0.00 | ILMN_2503275 |
| IGKV9-120_V00804$J00566_Ig_kappa_variable_9-120_12 | 1.8 | 0.00 | ILMN_1245042 |
| IGKV9-128_AJ231245_Ig_kappa_variable_9-128_15 | 1.7 | 0.00 | ILMN_1232948 |
| Igl-V1 | 2.3 | 0.00 | ILMN_1230696 |
| Igl-V1 | 2.3 | 0.00 | ILMN_1245146 |
| Igsf10 | -1.5 | 0.00 | ILMN_2674966 |
| Igsf10 | -1.5 | 0.00 | ILMN_1244809 |
| Igsf3 | -1.3 | 0.01 | ILMN_1229553 |
| Igsf3 | -1.3 | 0.00 | ILMN_1219384 |
| Il1b | 1.8 | 0.00 | ILMN_2777498 |
| Il4i1 | 1.2 | 0.03 | ILMN_2733778 |
| Il7 | 1.2 | 0.01 | ILMN_2630852 |
| Il8ra | 1.3 | 0.04 | ILMN_2588216 |
| Il8rb | 1.4 | 0.02 | ILMN_1223834 |
| Impdh1 | -1.1 | 0.01 | ILMN_2680540 |
| Iqca | 1.5 | 0.00 | ILMN_2428714 |
| Itga2b | 1.7 | 0.00 | ILMN_2911123 |
| Itga2b | 1.5 | 0.00 | ILMN_2732623 |
| Itgae | -1.2 | 0.04 | ILMN_2699898 |
| Itgb4 | 1.3 | 0.01 | ILMN_2634689 |
| Itm2a | -1.4 | 0.00 | ILMN_2607377 |
| Itpka | 1.3 | 0.01 | ILMN_2752431 |
| Ivns1abp | -1.2 | 0.02 | ILMN_1230799 |
| Kank4 | 1.3 | 0.04 | ILMN_2947948 |
| Kank4 | 1.4 | 0.00 | ILMN_2655586 |
| Kank4 | 1.2 | 0.00 | ILMN_1222183 |
| Kcne2 | -1.3 | 0.02 | ILMN_2794237 |
| Kcnh3 | 1.3 | 0.01 | ILMN_2726397 |
| Kdelc1 | -1.2 | 0.04 | ILMN_2601147 |
| Kdelr3 | -1.7 | 0.00 | ILMN_2675697 |
| Kif21a | 1.2 | 0.03 | ILMN_2718716 |
| Klk11 | 1.2 | 0.00 | ILMN_2780280 |
| Klra10 | -1.3 | 0.00 | ILMN_2642723 |
| Klra13 | -1.2 | 0.02 | ILMN_1230969 |
| Klra3 | -1.3 | 0.01 | ILMN_1237580 |
| Klra33 | -1.3 | 0.00 | ILMN_2982019 |
| Klra4 | -1.5 | 0.00 | ILMN_3031402 |
| Klre1 | -1.3 | 0.02 | ILMN_1239346 |
| Klri2 | -1.2 | 0.03 | ILMN_2651722 |
| Klrk1 | -1.2 | 0.03 | ILMN_2692703 |
| Kndc1 | 1.4 | 0.00 | ILMN_2757844 |
| Knsl5 | -1.5 | 0.00 | ILMN_1250752 |
| Krtap17-1 | -1.2 | 0.02 | ILMN_2458087 |
| Ky | 1.3 | 0.03 | ILMN_2906855 |
| Ky | 1.3 | 0.00 | ILMN_2506428 |
| L3mbtl3 | -1.2 | 0.01 | ILMN_2815479 |
| Lamc1 | -1.3 | 0.00 | ILMN_2774596 |
| Lamc1 | -1.4 | 0.00 | ILMN_2974798 |
| Lamp3 | -1.4 | 0.01 | ILMN_2738854 |
| Lamp3 | -1.4 | 0.01 | ILMN_2745667 |
| Lcp2 | 1.2 | 0.01 | ILMN_1216191 |
| Leprel2 | -1.2 | 0.02 | ILMN_1239380 |
| Lipf | 1.5 | 0.00 | ILMN_2863532 |
| Lmcd1 | -1.3 | 0.00 | ILMN_2907540 |
| Lmcd1 | -1.4 | 0.00 | ILMN_1247343 |
| LOC100038894 | -1.5 | 0.00 | ILMN_1252295 |
| LOC100039175 | -1.3 | 0.00 | ILMN_1225825 |
| LOC100039742 | 1.5 | 0.00 | ILMN_1213824 |
| LOC100040592 | 1.2 | 0.02 | ILMN_2654952 |
| LOC100042270 | 1.5 | 0.01 | ILMN_1249343 |
| LOC100045019 | 1.3 | 0.01 | ILMN_1249684 |
| LOC100045224 | -1.2 | 0.00 | ILMN_2551201 |
| LOC100046401 | -1.2 | 0.01 | ILMN_2774349 |
| LOC100046496 | 1.8 | 0.00 | ILMN_1229263 |
| LOC100046793 | 4.4 | 0.00 | ILMN_2463573 |
| LOC100046793 | 5.8 | 0.00 | ILMN_2469743 |
| LOC100047132 | 1.3 | 0.04 | ILMN_1231284 |
| LOC100047132 | 2.1 | 0.00 | ILMN_1218006 |
| LOC100047162 | 2.5 | 0.00 | ILMN_1222500 |
| LOC100047167 | -1.2 | 0.00 | ILMN_1222315 |
| LOC100047316 | 1.6 | 0.00 | ILMN_1215825 |
| LOC100047316 | 6.3 | 0.00 | ILMN_1247185 |
| LOC100047628 | 3.3 | 0.00 | ILMN_2450019 |
| LOC100047628 | 3.4 | 0.00 | ILMN_2704562 |
| LOC100047788 | 11.4 | 0.00 | ILMN_2677207 |
| LOC100047788 | 34.6 | 0.00 | ILMN_2633179 |
| LOC100048534 | 1.3 | 0.01 | ILMN_1225781 |
| LOC100048554 | 1.6 | 0.00 | ILMN_1238886 |
| LOC100048770 | 1.3 | 0.03 | ILMN_1219602 |
| LOC100048807 | 1.3 | 0.00 | ILMN_1225422 |
| LOC207685 | 1.7 | 0.00 | ILMN_1246353 |
| LOC213684 | 3.4 | 0.00 | ILMN_2745775 |
| LOC214403 | -1.3 | 0.00 | ILMN_2526875 |
| LOC217328 | 1.2 | 0.00 | ILMN_2667190 |
| LOC223653 | 1.2 | 0.03 | ILMN_2529254 |
| LOC223672 | 1.3 | 0.01 | ILMN_2671435 |
| LOC232060 | 1.9 | 0.00 | ILMN_1251451 |
| LOC232065 | 2.1 | 0.02 | ILMN_2632262 |
| LOC232067 | 1.8 | 0.02 | ILMN_2611798 |
| LOC238447 | 1.3 | 0.01 | ILMN_2774462 |
| LOC243431 | 1.9 | 0.00 | ILMN_2649286 |
| LOC268934 | 1.4 | 0.00 | ILMN_1252806 |
| LOC272683 | 2.2 | 0.00 | ILMN_2534635 |
| LOC331239 | 1.6 | 0.00 | ILMN_1232265 |
| LOC380653 | 1.2 | 0.00 | ILMN_2533911 |
| LOC380799 | 1.4 | 0.00 | ILMN_2609403 |
| LOC381000 | 1.3 | 0.01 | ILMN_1242666 |
| LOC381260 | 1.2 | 0.00 | ILMN_2526227 |
| LOC381283 | 1.9 | 0.00 | ILMN_1233840 |
| LOC381546 | 1.3 | 0.00 | ILMN_1248921 |
| LOC381670 | 1.2 | 0.01 | ILMN_1253279 |
| LOC381738 | 1.2 | 0.00 | ILMN_2538938 |
| LOC381774 | 3.6 | 0.00 | ILMN_1250715 |
| LOC382163 | 1.3 | 0.01 | ILMN_1237356 |
| LOC383196 | 2.2 | 0.00 | ILMN_1250188 |
| LOC383196 | 2.3 | 0.00 | ILMN_2612346 |
| LOC383576 | -1.4 | 0.00 | ILMN_2535430 |
| LOC384413 | 2.4 | 0.00 | ILMN_1258548 |
| LOC384415 | 3.0 | 0.00 | ILMN_2631505 |
| LOC384419 | 1.7 | 0.00 | ILMN_2631763 |
| LOC384422 | 1.7 | 0.00 | ILMN_1226583 |
| LOC385109 | 2.2 | 0.00 | ILMN_1243197 |
| LOC385256 | 1.2 | 0.04 | ILMN_1233406 |
| LOC385274 | 1.3 | 0.02 | ILMN_1251145 |
| LOC385277 | 1.3 | 0.01 | ILMN_2542553 |
| LOC385291 | 2.8 | 0.00 | ILMN_2608768 |
| LOC626152 | 1.2 | 0.03 | ILMN_1243900 |
| LOC626347 | 1.4 | 0.04 | ILMN_2690611 |
| LOC626583 | 14.2 | 0.00 | ILMN_1213431 |
| LOC630302 | 1.2 | 0.04 | ILMN_2724779 |
| LOC630305 | 1.6 | 0.00 | ILMN_1247021 |
| LOC633360 | 1.3 | 0.00 | ILMN_2423609 |
| LOC635601 | 1.4 | 0.02 | ILMN_2451103 |
| LOC635601 | 1.5 | 0.00 | ILMN_1233413 |
| LOC636696 | 1.4 | 0.00 | ILMN_2426436 |
| LOC636752 | 4.8 | 0.00 | ILMN_1220577 |
| LOC636875 | 1.9 | 0.00 | ILMN_2482821 |
| LOC636944 | 2.8 | 0.00 | ILMN_1230194 |
| LOC636944 | 2.7 | 0.00 | ILMN_1226667 |
| LOC637227 | 2.5 | 0.00 | ILMN_2722893 |
| LOC637260 | 1.2 | 0.04 | ILMN_2510884 |
| LOC637785 | 3.0 | 0.00 | ILMN_1259003 |
| LOC640340 | 2.7 | 0.00 | ILMN_2462709 |
| LOC640696 | 1.6 | 0.02 | ILMN_2501340 |
| LOC640739 | -1.2 | 0.01 | ILMN_1229210 |
| LOC664837 | 1.4 | 0.00 | ILMN_2537209 |
| LOC668549 | 1.5 | 0.01 | ILMN_2621519 |
| LOC669053 | 2.6 | 0.00 | ILMN_2699222 |
| LOC672339 | 2.1 | 0.00 | ILMN_1258680 |
| LOC672342 | 3.4 | 0.00 | ILMN_2490758 |
| LOC675572 | 1.3 | 0.00 | ILMN_2425028 |
| LOC676136 | 1.9 | 0.00 | ILMN_2465985 |
| LOC676136 | 1.7 | 0.00 | ILMN_2674666 |
| LOC676222 | 9.3 | 0.00 | ILMN_1244008 |
| LOC677643 | 4.3 | 0.00 | ILMN_1225733 |
| Lox | -1.9 | 0.00 | ILMN_2997494 |
| Loxl1 | -1.5 | 0.02 | ILMN_1255871 |
| Lrrc34 | 1.2 | 0.04 | ILMN_2786469 |
| Lrrc36 | 1.3 | 0.02 | ILMN_1235822 |
| Lrrc46 | 1.2 | 0.01 | ILMN_2737412 |
| Lrrc48 | 1.3 | 0.00 | ILMN_3071876 |
| Lrrc48 | 1.3 | 0.00 | ILMN_2594644 |
| Lrrc50 | 1.3 | 0.00 | ILMN_2696418 |
| Lum | -1.2 | 0.04 | ILMN_3001540 |
| Lypla2 | 1.2 | 0.02 | ILMN_1228132 |
| Lyz1 | -1.3 | 0.03 | ILMN_1247933 |
| Maged2 | -1.2 | 0.00 | ILMN_2600565 |
| Map3k12 | 1.2 | 0.04 | ILMN_2725370 |
| Mapk15 | 1.2 | 0.02 | ILMN_2859377 |
| Mboat2 | -1.5 | 0.00 | ILMN_1229343 |
| Mdh1b | 1.5 | 0.00 | ILMN_1217555 |
| Mdk | -1.5 | 0.00 | ILMN_3158499 |
| Meox1 | -1.5 | 0.00 | ILMN_1213886 |
| Mfap4 | -1.4 | 0.00 | ILMN_2633350 |
| Mfap5 | -1.6 | 0.00 | ILMN_1225835 |
| Mgl2 | -1.3 | 0.03 | ILMN_2707319 |
| Mgp | -1.2 | 0.00 | ILMN_2666018 |
| Mgst2 | 1.3 | 0.00 | ILMN_2613832 |
| Mlycd | 1.2 | 0.01 | ILMN_1249691 |
| Mmp14 | -1.3 | 0.00 | ILMN_1240726 |
| Mmp2 | -1.8 | 0.00 | ILMN_2678218 |
| Mmp9 | 1.6 | 0.00 | ILMN_2711075 |
| Mmrn1 | 1.2 | 0.04 | ILMN_1232435 |
| Mmrn2 | 1.3 | 0.03 | ILMN_2761918 |
| Mocos | 1.2 | 0.04 | ILMN_2906489 |
| Mpl | 1.2 | 0.03 | ILMN_1226499 |
| Mpl | 1.5 | 0.00 | ILMN_2855310 |
| Mrgpra2 | 1.3 | 0.00 | ILMN_2904108 |
| Mustn1 | -1.2 | 0.02 | ILMN_2658461 |
| Mycbpap | 1.2 | 0.01 | ILMN_2820831 |
| Myom2 | 1.3 | 0.00 | ILMN_1238982 |
| Myrip | 1.3 | 0.03 | ILMN_2665441 |
| Ndn | -1.6 | 0.00 | ILMN_2622374 |
| Nek5 | 1.3 | 0.00 | ILMN_1222147 |
| Neo1 | -1.3 | 0.02 | ILMN_2610156 |
| Neurl | 1.3 | 0.00 | ILMN_2625940 |
| Nfe2 | 1.5 | 0.00 | ILMN_2881620 |
| Nfkb1 | -1.2 | 0.01 | ILMN_2592476 |
| Nid1 | -1.4 | 0.00 | ILMN_2814005 |
| Nid2 | -1.3 | 0.02 | ILMN_2961933 |
| Nipsnap3a | -1.2 | 0.01 | ILMN_2657376 |
| Nkd2 | 1.5 | 0.00 | ILMN_1228631 |
| Nme5 | 1.3 | 0.00 | ILMN_2696232 |
| Nnat | -1.3 | 0.00 | ILMN_2755578 |
| Nol5a | -1.2 | 0.01 | ILMN_1245860 |
| Nrgn | 1.4 | 0.02 | ILMN_2645071 |
| Nrn1 | 1.5 | 0.00 | ILMN_2699052 |
| Nrp1 | -1.2 | 0.04 | ILMN_2669912 |
| Nt5e | 1.2 | 0.03 | ILMN_2813830 |
| Ntrk2 | 1.2 | 0.01 | ILMN_3138904 |
| Oas1g | 1.3 | 0.01 | ILMN_1253808 |
| Oas2 | 1.7 | 0.00 | ILMN_2670150 |
| Odc1 | 1.3 | 0.04 | ILMN_3008406 |
| Olfml2b | -1.3 | 0.00 | ILMN_2745551 |
| Oosp1 | 1.2 | 0.02 | ILMN_2707996 |
| OTTMUSG00000000971 | 1.4 | 0.01 | ILMN_2864309 |
| Pacrg | 1.3 | 0.00 | ILMN_2625279 |
| Parl | 1.2 | 0.02 | ILMN_2884646 |
| Pcdha6 | -1.3 | 0.00 | ILMN_3076948 |
| Pcdha7 | -1.3 | 0.01 | ILMN_1250379 |
| Pcdhb3 | -1.3 | 0.00 | ILMN_1253649 |
| Pcdhga4 | 1.2 | 0.04 | ILMN_2736902 |
| Pcolce | -1.2 | 0.00 | ILMN_1253741 |
| Pcolce2 | -1.4 | 0.03 | ILMN_2678421 |
| Pcp4l1 | 1.6 | 0.00 | ILMN_1218127 |
| Pcp4l1 | 1.6 | 0.00 | ILMN_1254622 |
| Pcsk6 | 1.4 | 0.00 | ILMN_2637094 |
| Pdgfrb | -1.3 | 0.00 | ILMN_2903972 |
| Pdgfrl | -1.3 | 0.00 | ILMN_2903926 |
| Pdia2 | 1.2 | 0.02 | ILMN_1215125 |
| Pdia5 | -1.3 | 0.00 | ILMN_2607066 |
| Pdia5 | -1.4 | 0.00 | ILMN_1255177 |
| Peg3 | -1.5 | 0.00 | ILMN_1245246 |
| Phex | -1.5 | 0.00 | ILMN_1243830 |
| Phf10 | -1.2 | 0.04 | ILMN_1227540 |
| Phf11 | 1.2 | 0.04 | ILMN_2696491 |
| Phf11 | 1.3 | 0.01 | ILMN_2696492 |
| Phlda2 | 1.2 | 0.01 | ILMN_2425029 |
| Pitpnm1 | 1.2 | 0.01 | ILMN_2794116 |
| Pla2g1b | -1.4 | 0.00 | ILMN_2845906 |
| Plcg2 | 1.2 | 0.04 | ILMN_2601833 |
| Pld1 | -1.2 | 0.02 | ILMN_2595408 |
| Plvap | -1.2 | 0.02 | ILMN_2984110 |
| Poln | 1.2 | 0.01 | ILMN_2786217 |
| Pomgnt1 | -1.2 | 0.04 | ILMN_2722353 |
| Ppargc1a | 1.2 | 0.03 | ILMN_2710139 |
| Ppbp | 2.0 | 0.00 | ILMN_1228102 |
| Ppbp | 1.9 | 0.00 | ILMN_2908435 |
| Ppic | -1.2 | 0.01 | ILMN_2810882 |
| Ppic | -1.4 | 0.00 | ILMN_2700797 |
| Ppm1b | -1.2 | 0.02 | ILMN_2729953 |
| Ppp1r14d | -1.2 | 0.04 | ILMN_1253015 |
| Prf1 | -1.3 | 0.01 | ILMN_1228333 |
| Prg2 | 1.8 | 0.00 | ILMN_2613878 |
| Prkar1b | 1.2 | 0.03 | ILMN_1235354 |
| Prkar2b | 1.2 | 0.00 | ILMN_1253819 |
| Prodh | 1.3 | 0.04 | ILMN_2636666 |
| Prpf8 | -1.2 | 0.02 | ILMN_2637661 |
| Pscd4 | 1.2 | 0.02 | ILMN_2416628 |
| Psp | -1.3 | 0.00 | ILMN_1245726 |
| Ptn | -1.2 | 0.04 | ILMN_2638114 |
| Ptpla | -1.3 | 0.00 | ILMN_2734712 |
| Ptplad2 | 1.2 | 0.00 | ILMN_1245615 |
| Ptprd | -1.4 | 0.01 | ILMN_1254974 |
| Ptprd | -1.3 | 0.00 | ILMN_3103904 |
| Ptprd | -1.4 | 0.00 | ILMN_2501929 |
| Ptprm | -1.2 | 0.03 | ILMN_1246777 |
| Qpct | -1.3 | 0.00 | ILMN_2814865 |
| Rab11fip4 | 1.2 | 0.02 | ILMN_1236245 |
| Rabep1 | -1.1 | 0.03 | ILMN_2999748 |
| Rabl2a | 1.2 | 0.01 | ILMN_2593578 |
| Rarres1 | 1.3 | 0.00 | ILMN_2664202 |
| Rassf10 | 1.3 | 0.00 | ILMN_2636005 |
| Rbm38 | 1.2 | 0.04 | ILMN_1240979 |
| Rbms1 | -1.2 | 0.00 | ILMN_2883990 |
| Reck | -1.2 | 0.02 | ILMN_2769877 |
| Reln | 1.2 | 0.02 | ILMN_2704257 |
| Retnla | -1.9 | 0.01 | ILMN_1226472 |
| Retnlg | 1.6 | 0.03 | ILMN_2656504 |
| Rfc2 | 1.2 | 0.04 | ILMN_2894497 |
| Rftn2 | -1.2 | 0.00 | ILMN_2795178 |
| Rfx2 | 1.2 | 0.03 | ILMN_1217353 |
| Rhobtb3 | -1.2 | 0.02 | ILMN_1221243 |
| Ric3 | 1.4 | 0.00 | ILMN_3072117 |
| Rims3 | 1.2 | 0.04 | ILMN_2488599 |
| Robo1 | -1.3 | 0.00 | ILMN_1244484 |
| Robo2 | -1.3 | 0.00 | ILMN_1227705 |
| Rpl13a | -1.2 | 0.04 | ILMN_2707834 |
| Rsad2 | 1.5 | 0.00 | ILMN_1225204 |
| Rshl3 | 1.3 | 0.00 | ILMN_1241366 |
| Rsph1 | 1.2 | 0.03 | ILMN_2441534 |
| S100a8 | 2.2 | 0.00 | ILMN_2710905 |
| S100a9 | 2.5 | 0.00 | ILMN_2803674 |
| Scara5 | -1.2 | 0.01 | ILMN_3008068 |
| Scel | 1.4 | 0.03 | ILMN_2682120 |
| Schip1 | -1.2 | 0.00 | ILMN_1244514 |
| scl0001849.1_2273 | 1.4 | 0.01 | ILMN_1250116 |
| scl0002507.1_236 | -1.3 | 0.01 | ILMN_2497957 |
| scl0002540.1_6 | -1.2 | 0.00 | ILMN_1254034 |
| scl0003799.1_2 | -1.2 | 0.00 | ILMN_1236010 |
| Scn3a | -1.4 | 0.03 | ILMN_1218700 |
| Scn3b | -1.4 | 0.00 | ILMN_1215136 |
| Scube2 | -1.4 | 0.00 | ILMN_2636183 |
| Sdk1 | -1.3 | 0.03 | ILMN_2728889 |
| Selp | 1.2 | 0.01 | ILMN_1236889 |
| Serpina1d | 1.2 | 0.02 | ILMN_1225570 |
| Serpina3f | 1.6 | 0.04 | ILMN_2742861 |
| Serpina3g | 1.8 | 0.00 | ILMN_2725927 |
| Serpinh1 | -1.2 | 0.01 | ILMN_2777359 |
| Sestd1 | -1.2 | 0.03 | ILMN_2676770 |
| Sf3b4 | -1.2 | 0.03 | ILMN_3051392 |
| Sfxn1 | 1.3 | 0.00 | ILMN_1233606 |
| Sfxn4 | 1.3 | 0.02 | ILMN_2776377 |
| Shh | -1.2 | 0.04 | ILMN_2802979 |
| Shprh | -1.2 | 0.01 | ILMN_3021465 |
| Six1 | 1.2 | 0.04 | ILMN_2904117 |
| Slc11a1 | 1.3 | 0.01 | ILMN_2983516 |
| Slc22a1 | 1.2 | 0.02 | ILMN_1247071 |
| Slc25a27 | -1.1 | 0.04 | ILMN_2858938 |
| Slc25a37 | 1.3 | 0.03 | ILMN_2696610 |
| Slc2a1 | 1.2 | 0.01 | ILMN_1258159 |
| Slc2a3 | 1.8 | 0.00 | ILMN_2616565 |
| Slc2a6 | 1.4 | 0.00 | ILMN_2618918 |
| Slc4a1 | 1.6 | 0.03 | ILMN_1227675 |
| Slc6a2 | 1.3 | 0.04 | ILMN_2922875 |
| Slfn1 | 1.3 | 0.03 | ILMN_2663930 |
| Slit2 | -1.3 | 0.01 | ILMN_1253797 |
| Slpi | 1.5 | 0.00 | ILMN_1256817 |
| Smarca1 | -1.3 | 0.00 | ILMN_1253650 |
| Smpdl3b | 1.2 | 0.01 | ILMN_2602938 |
| Smyd3 | 1.2 | 0.03 | ILMN_1231094 |
| Snai2 | -1.3 | 0.00 | ILMN_2647563 |
| Snap91 | 1.1 | 0.04 | ILMN_1240692 |
| Snca | 1.6 | 0.03 | ILMN_3136638 |
| Snca | 1.6 | 0.01 | ILMN_3161601 |
| Sncg | 1.2 | 0.01 | ILMN_2939277 |
| Sncg | 1.7 | 0.00 | ILMN_2598478 |
| Sod3 | -1.3 | 0.00 | ILMN_1241892 |
| Son | -1.2 | 0.01 | ILMN_2763871 |
| Sox17 | 1.2 | 0.04 | ILMN_2747986 |
| Sparc | -1.4 | 0.00 | ILMN_3059326 |
| Sparc | -1.3 | 0.00 | ILMN_3136561 |
| Speer4c | 1.2 | 0.04 | ILMN_1245154 |
| Spire2 | 1.2 | 0.01 | ILMN_2640995 |
| Spnb1 | 1.4 | 0.00 | ILMN_2660754 |
| Spnb1 | 1.7 | 0.00 | ILMN_2909238 |
| Srpx | -1.3 | 0.00 | ILMN_1238000 |
| Srpx | -1.5 | 0.00 | ILMN_2629486 |
| Srpx2 | -1.3 | 0.01 | ILMN_2698728 |
| Srpx2 | -1.5 | 0.00 | ILMN_2818294 |
| Srxn1 | 1.2 | 0.01 | ILMN_2677772 |
| St3gal2 | -1.2 | 0.03 | ILMN_1256849 |
| Stc1 | -1.4 | 0.00 | ILMN_2777319 |
| Steap2 | -1.2 | 0.01 | ILMN_2797726 |
| Supt3h | 1.2 | 0.01 | ILMN_1213872 |
| Suv420h1 | -1.2 | 0.02 | ILMN_2996601 |
| Synj2bp | -1.2 | 0.04 | ILMN_2752867 |
| Syp | 1.2 | 0.00 | ILMN_2630182 |
| Syt5 | 1.5 | 0.00 | ILMN_1220815 |
| Tagln | -1.2 | 0.01 | ILMN_1243652 |
| Tbx2 | -1.2 | 0.04 | ILMN_2773918 |
| Tbxa2r | -1.3 | 0.04 | ILMN_1248837 |
| Tbxa2r | -1.3 | 0.01 | ILMN_2737840 |
| Tceal8 | -1.3 | 0.00 | ILMN_2861493 |
| Tcrg-V4 | 1.3 | 0.01 | ILMN_2491839 |
| Tctex1d2 | 1.2 | 0.00 | ILMN_2720925 |
| Tekt1 | 1.3 | 0.01 | ILMN_2700097 |
| Tekt1 | 1.3 | 0.00 | ILMN_1239718 |
| Tex14 | -1.3 | 0.00 | ILMN_2620323 |
| Tgfb3 | -1.2 | 0.01 | ILMN_2748966 |
| Thbs3 | -1.3 | 0.01 | ILMN_2840956 |
| Thbs3 | -1.3 | 0.00 | ILMN_2840958 |
| Thbs3 | -1.4 | 0.00 | ILMN_2654651 |
| Thy1 | 1.3 | 0.00 | ILMN_2644350 |
| Tiam1 | 1.2 | 0.03 | ILMN_2737710 |
| Timp1 | -1.3 | 0.01 | ILMN_3103896 |
| Tlr7 | 1.3 | 0.00 | ILMN_1245354 |
| Tmem132a | -1.2 | 0.02 | ILMN_2750588 |
| Tmem132c | -1.3 | 0.00 | ILMN_2625148 |
| Tmem45a | -1.2 | 0.03 | ILMN_2535227 |
| Tmod2 | 1.2 | 0.01 | ILMN_3114546 |
| Tna | -1.3 | 0.00 | ILMN_2434661 |
| Tnc | -1.3 | 0.03 | ILMN_2463180 |
| Tnfrsf11b | -1.3 | 0.00 | ILMN_2513826 |
| Tnfrsf17 | 1.2 | 0.04 | ILMN_2854497 |
| Tnfsf14 | 1.2 | 0.01 | ILMN_2851576 |
| Tns3 | -1.2 | 0.00 | ILMN_1247133 |
| Trem3 | 1.4 | 0.01 | ILMN_2915303 |
| Treml1 | 1.9 | 0.00 | ILMN_2523169 |
| Trp53inp2 | -1.2 | 0.03 | ILMN_2457585 |
| Trpv4 | 1.2 | 0.03 | ILMN_1231520 |
| Trub2 | 1.2 | 0.01 | ILMN_2983686 |
| Tsnaxip1 | 1.2 | 0.03 | ILMN_2881498 |
| Tsnaxip1 | 1.3 | 0.00 | ILMN_1240576 |
| Tspan17 | 1.2 | 0.03 | ILMN_2817714 |
| Tspan6 | -1.2 | 0.04 | ILMN_2515784 |
| Ttc18 | 1.4 | 0.00 | ILMN_1213134 |
| Ttc29 | 1.3 | 0.00 | ILMN_2976989 |
| Ttc29 | 1.3 | 0.00 | ILMN_2976991 |
| Ttyh3 | -1.2 | 0.03 | ILMN_1221311 |
| Twsg1 | -1.3 | 0.00 | ILMN_2463979 |
| Ubd | 1.3 | 0.00 | ILMN_2426853 |
| Ube2o | 1.3 | 0.00 | ILMN_1245815 |
| Ubtd2 | -1.3 | 0.03 | ILMN_2822552 |
| Ugt8a | -1.2 | 0.02 | ILMN_1239557 |
| Uhrf1bp1 | 1.1 | 0.02 | ILMN_2487847 |
| Veph1 | -1.3 | 0.00 | ILMN_2744514 |
| Vldlr | -1.2 | 0.03 | ILMN_2515601 |
| Vldlr | -1.3 | 0.00 | ILMN_1218264 |
| Vnn3 | 1.2 | 0.04 | ILMN_2491202 |
| Vtn | -1.2 | 0.02 | ILMN_1234111 |
| Wdr66 | 1.2 | 0.03 | ILMN_2423071 |
| Wdr78 | 1.2 | 0.03 | ILMN_2687391 |
| Wfdc3 | 1.3 | 0.00 | ILMN_1229131 |
| Wfdc6a | -1.3 | 0.01 | ILMN_2883709 |
| Wfdc6b | -1.2 | 0.02 | ILMN_3140659 |
| Wfdc6b | -1.3 | 0.00 | ILMN_3062983 |
| Wipi1 | -1.2 | 0.02 | ILMN_2834677 |
| Wisp1 | -1.3 | 0.01 | ILMN_2492264 |
| Xpnpep1 | -1.2 | 0.02 | ILMN_2428961 |
| Xpnpep2 | 1.3 | 0.00 | ILMN_1248998 |
| Zap70 | 1.2 | 0.01 | ILMN_2513870 |
| Zbtb7c | 1.3 | 0.02 | ILMN_2622500 |
| Zcchc18 | 1.3 | 0.01 | ILMN_2649172 |
| Zfp260 | -1.2 | 0.03 | ILMN_2441326 |
| Zfp276 | 1.2 | 0.03 | ILMN_1222031 |
| Zfp533 | -1.2 | 0.04 | ILMN_2472730 |
| Zmynd10 | 1.3 | 0.03 | ILMN_1252284 |
