# Supplementary material for: Age-Dependent Decline in Mouse Lung Regeneration with Loss of Lung Fibroblast Clonogenicity and Increased Myofibroblastic Differentiation
Source: PLoS One. 2011 Aug 30;6(8):e23232. doi: 10.1371/journal.pone.0023232 (PMC3166052; doi:10.1371/journal.pone.0023232)
Supplement: Table S2 — Microarray analysis of genes with significant differences (P<0.05) in differential regulation (fold change, compared to pre-PNX samples) between 9 and 3 month mice 1 day after PNX. (DOC) [file pone.0023232.s002.doc]

**Table S2** – Microarray analysis of genes with significant differences (P<0.05) in differential regulation (fold change, compared to pre-PNX samples) between 9 and 3 month mice 1 day after PNX

| SYMBOL | 3 mo FC | 9 mo FC | P | PROBE_ID |
| --- | --- | --- | --- | --- |
| 0610007C21Rik | -1.2 | -1.7 | 0.01 | ILMN_3033922 |
| 1110001P04Rik | -1.4 | -1.6 | 0.03 | ILMN_1248658 |
| 1110017D15Rik | -1.5 | -1.6 | 0.04 | ILMN_2617920 |
| 1110029I05Rik | -1.2 | -1.5 | 0.04 | ILMN_2427971 |
| 1110029I05Rik | -1.4 | -2.1 | 0.01 | ILMN_2488405 |
| 1110046J11Rik | -1.4 | -2.4 | 0.02 | ILMN_2513451 |
| 1190003M12Rik | -1.6 | -2.6 | 0.04 | ILMN_2638473 |
| 1190003M12Rik | -1.7 | -2.2 | 0.02 | ILMN_2856588 |
| 1200009O22Rik | -1.7 | -3.4 | 0.01 | ILMN_2615431 |
| 1200016G03Rik | 1.5 | 2.2 | 0.01 | ILMN_1222685 |
| 1500005K14Rik | 1.3 | 1.8 | 0.03 | ILMN_1259764 |
| 1700016J18Rik | -1.4 | -1.8 | 0.03 | ILMN_1225662 |
| 1700024G13Rik | -1.3 | -2.1 | 0.04 | ILMN_2711761 |
| 1700065O13Rik | -1.8 | -2.3 | 0.01 | ILMN_2845839 |
| 1810013B01Rik | -1.5 | -1.9 | 0.01 | ILMN_2774244 |
| 2210018M11Rik | -1.3 | -1.5 | 0.04 | ILMN_2447538 |
| 2210414H16Rik | -1.3 | -1.7 | 0.04 | ILMN_1241919 |
| 2210414H16Rik | -1.6 | -2.0 | 0.00 | ILMN_2523958 |
| 2310005E10Rik | -1.3 | -1.5 | 0.02 | ILMN_2733753 |
| 2310014L17Rik | 2.0 | 1.3 | 0.01 | ILMN_2946672 |
| 2310047B19Rik | -1.3 | -1.5 | 0.02 | ILMN_2716693 |
| 2310047D13Rik | 2.5 | 2.1 | 0.04 | ILMN_2909105 |
| 2310047D13Rik | 3.1 | 2.2 | 0.01 | ILMN_2543108 |
| 2410008K03Rik | -1.3 | -1.7 | 0.01 | ILMN_2759563 |
| 2410025L10Rik | -1.5 | -1.3 | 0.03 | ILMN_2971721 |
| 2700029M09Rik | 1.6 | 1.3 | 0.03 | ILMN_1253477 |
| 2810025M15Rik | -1.2 | -1.7 | 0.01 | ILMN_1213985 |
| 2810026P18Rik | -1.2 | 1.5 | 0.00 | ILMN_2513173 |
| 2810417H13Rik | 2.0 | 1.2 | 0.03 | ILMN_1223045 |
| 2810453I06Rik | -1.4 | -2.1 | 0.03 | ILMN_2510771 |
| 2810453I06Rik | -1.4 | -2.1 | 0.01 | ILMN_2695228 |
| 2900006A08Rik | -1.6 | -1.3 | 0.04 | ILMN_2439505 |
| 2900054C01Rik | -1.2 | -1.6 | 0.00 | ILMN_2438962 |
| 3300001G02Rik | -1.2 | -1.7 | 0.02 | ILMN_2990229 |
| 3300002A11Rik | -1.9 | -2.5 | 0.01 | ILMN_2440486 |
| 4632408A20Rik | -1.2 | -1.5 | 0.01 | ILMN_1247032 |
| 4732496O08Rik | -1.6 | -1.3 | 0.02 | ILMN_1212648 |
| 4930403O06Rik | 1.9 | 1.4 | 0.02 | ILMN_2711651 |
| 4930455F23Rik | -1.3 | -1.5 | 0.04 | ILMN_2908953 |
| 4933403G17Rik | 1.2 | 1.5 | 0.03 | ILMN_2735849 |
| 4933404M02Rik | -1.3 | -2.0 | 0.00 | ILMN_1249756 |
| 4933426M11Rik | 1.3 | 1.6 | 0.03 | ILMN_1254551 |
| 5330403J18Rik | -1.1 | 1.6 | 0.00 | ILMN_2566272 |
| 6720467C03Rik | 1.5 | 1.2 | 0.02 | ILMN_2837189 |
| 9130020K20Rik | 1.3 | 1.6 | 0.03 | ILMN_2462678 |
| 9330175B01Rik | -1.3 | -2.1 | 0.02 | ILMN_1232055 |
| 9430080K19Rik | -1.5 | -1.2 | 0.01 | ILMN_2500370 |
| 9530027K23Rik | -1.0 | 1.5 | 0.00 | ILMN_1243939 |
| 9630007E23Rik | -1.2 | -1.7 | 0.01 | ILMN_1253602 |
| 9830169E20Rik | 1.6 | 2.1 | 0.04 | ILMN_2571683 |
| A130026C10Rik | 1.1 | 1.6 | 0.02 | ILMN_1243621 |
| A330102K04Rik | -1.1 | -1.9 | 0.02 | ILMN_1252157 |
| Abca15 | -1.2 | -1.6 | 0.00 | ILMN_2598660 |
| Abcc10 | -1.3 | -1.5 | 0.01 | ILMN_1238608 |
| Ace2 | -1.1 | -1.5 | 0.00 | ILMN_1241584 |
| Acot1 | 1.2 | 1.6 | 0.04 | ILMN_3062267 |
| Actb | -3.4 | -4.9 | 0.03 | ILMN_2588055 |
| Actb | -3.2 | -5.7 | 0.02 | ILMN_2846865 |
| Actc1 | 1.4 | 2.7 | 0.03 | ILMN_2598916 |
| Actg2 | 1.6 | 2.6 | 0.01 | ILMN_2839313 |
| Actn1 | 1.2 | 2.1 | 0.00 | ILMN_2844996 |
| Actn2 | 2.8 | 1.6 | 0.04 | ILMN_2797061 |
| Actn2 | 2.9 | 1.6 | 0.03 | ILMN_2764727 |
| Acy3 | -1.1 | -1.5 | 0.01 | ILMN_1240165 |
| Adam15 | 1.3 | 2.1 | 0.03 | ILMN_3139103 |
| Adamts9 | -1.2 | 2.8 | 0.00 | ILMN_1257724 |
| Adamtsl5 | 1.4 | 1.9 | 0.00 | ILMN_2692399 |
| Adh1 | 1.1 | -1.5 | 0.00 | ILMN_2850077 |
| Adi1 | -1.1 | -1.7 | 0.00 | ILMN_3143557 |
| Adssl1 | 1.6 | 1.1 | 0.00 | ILMN_2958099 |
| Ak1 | 1.7 | 1.3 | 0.01 | ILMN_2667805 |
| Akp2 | 2.0 | 3.3 | 0.03 | ILMN_2661287 |
| Akr1b7 | -1.3 | -1.8 | 0.01 | ILMN_1238042 |
| Akr7a5 | -1.4 | -1.7 | 0.04 | ILMN_1256518 |
| Alas2 | -1.2 | -2.1 | 0.01 | ILMN_2675874 |
| Aldh3b1 | -1.3 | -1.6 | 0.01 | ILMN_2645793 |
| Alkbh7 | -1.2 | -1.5 | 0.02 | ILMN_1251766 |
| Alox5ap | 1.1 | -1.5 | 0.00 | ILMN_1215791 |
| Amigo2 | 1.3 | 1.8 | 0.00 | ILMN_2678355 |
| Angptl4 | 1.8 | 5.3 | 0.02 | ILMN_2759365 |
| Ank | -1.4 | -1.9 | 0.01 | ILMN_1232621 |
| Ankrd1 | 1.8 | 2.9 | 0.04 | ILMN_2950286 |
| Ankrd23 | 2.9 | 1.7 | 0.00 | ILMN_2741117 |
| Anp32a | -1.4 | -1.7 | 0.04 | ILMN_1222637 |
| Anxa9 | 1.5 | 1.2 | 0.00 | ILMN_2715226 |
| Ap2a1 | 1.4 | 1.9 | 0.04 | ILMN_2604688 |
| Aph1a | -1.3 | -1.7 | 0.03 | ILMN_2588759 |
| Arl3 | 1.5 | 1.2 | 0.01 | ILMN_1238801 |
| Arpc2 | 1.2 | 1.5 | 0.04 | ILMN_2698958 |
| Ars2 | 1.1 | 1.7 | 0.04 | ILMN_1225615 |
| Asgr1 | -1.8 | -2.7 | 0.03 | ILMN_2608703 |
| Atmin | -1.2 | -1.7 | 0.01 | ILMN_2701824 |
| AU018778 | -1.3 | -2.0 | 0.01 | ILMN_1238140 |
| AV249152 | -1.4 | -1.7 | 0.04 | ILMN_2970672 |
| AW123240 | -1.6 | -1.2 | 0.02 | ILMN_2513570 |
| Azgp1 | -1.4 | -2.3 | 0.01 | ILMN_2837080 |
| B130015M16Rik | 1.2 | 1.8 | 0.02 | ILMN_2565252 |
| B130066H02Rik | 1.2 | 1.8 | 0.00 | ILMN_1238902 |
| B230380D07Rik | -1.3 | -1.8 | 0.03 | ILMN_1214163 |
| B230396O12Rik | -1.2 | -1.7 | 0.02 | ILMN_2806479 |
| B930041F14Rik | -1.2 | -1.7 | 0.01 | ILMN_1250201 |
| Bahcc1 | -1.4 | -1.7 | 0.02 | ILMN_1242613 |
| Banf1 | 1.2 | 1.5 | 0.03 | ILMN_2698392 |
| Banf1 | 1.2 | 1.6 | 0.00 | ILMN_3158909 |
| Bat1a | -1.7 | -1.1 | 0.00 | ILMN_2670895 |
| BC006779 | -1.0 | 1.9 | 0.00 | ILMN_1226639 |
| BC021614 | -1.8 | -1.4 | 0.03 | ILMN_2591342 |
| BC064033 | 1.6 | 1.2 | 0.02 | ILMN_2771760 |
| Bcat2 | 1.6 | 1.2 | 0.04 | ILMN_2794608 |
| Bcl2l1 | 1.7 | 3.0 | 0.01 | ILMN_2698430 |
| Bcl7a | -1.5 | -2.0 | 0.02 | ILMN_1252601 |
| Bex2 | -1.6 | -2.8 | 0.04 | ILMN_1232107 |
| Bicd2 | 1.4 | 2.0 | 0.01 | ILMN_3119245 |
| Birc5 | 1.6 | -1.1 | 0.00 | ILMN_2632712 |
| Bmx | -1.3 | -1.8 | 0.04 | ILMN_2762528 |
| Bzw2 | 1.6 | 1.1 | 0.04 | ILMN_2644587 |
| C030026E19Rik | -1.1 | -1.6 | 0.00 | ILMN_2492491 |
| C030027H14Rik | 1.3 | 1.8 | 0.03 | ILMN_1239464 |
| C030046I01Rik | 1.3 | 1.5 | 0.03 | ILMN_1244149 |
| C130073O12Rik | 1.6 | 1.2 | 0.03 | ILMN_2585867 |
| C230029D21Rik | 1.1 | 1.5 | 0.01 | ILMN_1243664 |
| C330023M02Rik | 1.2 | 1.5 | 0.00 | ILMN_2833243 |
| C530027B15Rik | -1.3 | 1.7 | 0.00 | ILMN_1233501 |
| C530043K16Rik | 1.2 | 1.7 | 0.04 | ILMN_2646341 |
| C76566 | 1.5 | 1.7 | 0.00 | ILMN_2608622 |
| Capn2 | -1.2 | 1.6 | 0.00 | ILMN_1235077 |
| Car14 | -1.3 | -1.6 | 0.03 | ILMN_2973824 |
| Ccbp2 | 1.1 | 1.5 | 0.01 | ILMN_2650557 |
| Ccdc80 | 2.0 | 3.0 | 0.02 | ILMN_1234824 |
| Ccl27 | -1.2 | -1.7 | 0.01 | ILMN_3146018 |
| Ccl3 | 1.7 | -1.1 | 0.01 | ILMN_1253919 |
| Cd34 | 1.4 | 1.7 | 0.03 | ILMN_2714360 |
| Cd93 | 1.5 | 2.8 | 0.02 | ILMN_1226829 |
| Cd93 | 1.3 | 2.6 | 0.01 | ILMN_1226329 |
| Cdh16 | -2.6 | -1.8 | 0.01 | ILMN_2670038 |
| Cdh5 | -1.5 | 1.2 | 0.00 | ILMN_2866951 |
| Cdkn1a | 0.6 | 4.4 | 0.04 | ILMN_2634083 |
| Cdkn1a | 1.6 | 5.3 | 0.03 | ILMN_2846775 |
| Cdkn1a | 1.3 | 4.9 | 0.02 | ILMN_2846776 |
| Cenpt | 1.2 | 1.8 | 0.04 | ILMN_2653972 |
| Cep63 | -1.2 | -1.5 | 0.02 | ILMN_1220309 |
| Ces3 | -1.3 | -1.5 | 0.03 | ILMN_2625893 |
| Chad | -1.2 | -2.3 | 0.01 | ILMN_2659340 |
| Chrnb1 | 1.3 | 1.6 | 0.04 | ILMN_3001481 |
| Chrnb1 | 1.4 | 1.7 | 0.01 | ILMN_2685921 |
| Chst12 | 1.6 | 1.5 | 0.03 | ILMN_2710205 |
| Ckm | 11.8 | 4.3 | 0.03 | ILMN_2608804 |
| Cldn23 | -1.3 | -1.9 | 0.00 | ILMN_2758545 |
| Clec4d | 4.0 | 1.3 | 0.01 | ILMN_2705628 |
| Clec4g | -2.0 | -2.8 | 0.00 | ILMN_1234165 |
| Clec7a | -1.3 | -1.8 | 0.01 | ILMN_2653132 |
| Col18a1 | 1.2 | 2.2 | 0.03 | ILMN_2937542 |
| Col18a1 | 1.4 | 3.3 | 0.01 | ILMN_2735184 |
| Col3a1 | 2.0 | 3.2 | 0.02 | ILMN_1258629 |
| Col4a2 | 1.5 | 2.6 | 0.04 | ILMN_2822579 |
| Col4a4 | 1.2 | 1.8 | 0.02 | ILMN_2679871 |
| Col5a1 | 1.2 | 2.1 | 0.03 | ILMN_2748402 |
| Col6a1 | 1.5 | 2.2 | 0.03 | ILMN_2768087 |
| Col6a2 | 1.9 | 2.7 | 0.00 | ILMN_1216661 |
| Col6a3 | 1.5 | 2.1 | 0.01 | ILMN_1249220 |
| Cox7a2l | -1.3 | -1.8 | 0.04 | ILMN_2888713 |
| Cox7a2l | -1.3 | -1.6 | 0.03 | ILMN_1242107 |
| Cpeb4 | 1.6 | 2.2 | 0.04 | ILMN_1257184 |
| Cpsf1 | 1.4 | 1.8 | 0.02 | ILMN_3139668 |
| Crebl2 | -1.2 | -1.7 | 0.00 | ILMN_2632162 |
| Csnk2a2 | -1.7 | -1.2 | 0.04 | ILMN_2739039 |
| Csnk2a2 | -1.5 | -1.3 | 0.04 | ILMN_1228485 |
| Ctsa | -1.3 | -2.1 | 0.01 | ILMN_2669714 |
| Ctsf | -1.8 | -2.3 | 0.00 | ILMN_2615879 |
| Cyp20a1 | 1.5 | 1.6 | 0.01 | ILMN_2715585 |
| Cytl1 | 1.1 | -1.6 | 0.00 | ILMN_2877029 |
| D030017L14Rik | 1.2 | 1.5 | 0.04 | ILMN_2451400 |
| D0H4S114 | -1.7 | -2.8 | 0.00 | ILMN_2680054 |
| D230019G01Rik | -1.5 | -1.2 | 0.04 | ILMN_1252675 |
| D330001D04Rik | 1.1 | 1.5 | 0.00 | ILMN_1252642 |
| D330028D13Rik | -1.3 | -1.8 | 0.03 | ILMN_1217670 |
| D830014E11Rik | -1.2 | -1.5 | 0.01 | ILMN_2696171 |
| Ddb1 | 1.4 | 1.8 | 0.04 | ILMN_2620930 |
| Ddef2 | 1.1 | 1.6 | 0.01 | ILMN_3121578 |
| Ddo | -1.0 | -1.6 | 0.00 | ILMN_2808191 |
| Dmkn | 4.2 | 2.8 | 0.00 | ILMN_1229763 |
| Dmkn | 3.9 | 2.3 | 0.00 | ILMN_3105563 |
| Dtna | 2.2 | 1.4 | 0.03 | ILMN_1221805 |
| Dusp2 | -1.6 | -1.2 | 0.04 | ILMN_1242406 |
| E030038G15Rik | 1.2 | 1.8 | 0.01 | ILMN_1229701 |
| E2f4 | 1.3 | 2.3 | 0.00 | ILMN_2794796 |
| E430002G05Rik | -1.4 | -2.5 | 0.02 | ILMN_2870864 |
| Ear10 | -1.4 | -1.9 | 0.01 | ILMN_1235857 |
| Ecm1 | 1.8 | 2.9 | 0.02 | ILMN_2947526 |
| Edg7 | -1.4 | -2.0 | 0.00 | ILMN_3009440 |
| Ednrb | -1.2 | -2.5 | 0.01 | ILMN_2589640 |
| Eef1a2 | 3.7 | 1.6 | 0.03 | ILMN_2666990 |
| Eef1a2 | 5.2 | 2.4 | 0.00 | ILMN_2971559 |
| Efcab1 | -1.7 | -2.2 | 0.01 | ILMN_2917280 |
| Efnb1 | -2.1 | -1.4 | 0.03 | ILMN_2698443 |
| EG243642 | 1.4 | 1.7 | 0.03 | ILMN_2534332 |
| EG382843 | -1.7 | -2.2 | 0.04 | ILMN_1223807 |
| EG432448 | 1.2 | 1.5 | 0.04 | ILMN_2741950 |
| EG638695 | -1.4 | -3.5 | 0.03 | ILMN_2809443 |
| Eif4g2 | 1.4 | 1.5 | 0.02 | ILMN_2458030 |
| Eif5 | 1.4 | 1.8 | 0.01 | ILMN_3072536 |
| Elfn1 | -1.3 | -1.9 | 0.00 | ILMN_2606433 |
| Eln | 1.8 | 3.7 | 0.01 | ILMN_2697304 |
| Emilin1 | 1.6 | 1.9 | 0.04 | ILMN_2668462 |
| Emp2 | -1.9 | -1.7 | 0.02 | ILMN_2598103 |
| Eno3 | 3.2 | 1.3 | 0.00 | ILMN_2757569 |
| Ep300 | -1.1 | -1.6 | 0.01 | ILMN_2800358 |
| Epb4.1l3 | -1.6 | -2.1 | 0.01 | ILMN_1244272 |
| Epb4.1l4b | -1.2 | -1.7 | 0.01 | ILMN_2773215 |
| Epb4.1l4b | -1.2 | -1.6 | 0.01 | ILMN_2775064 |
| Ephx1 | -1.7 | -2.7 | 0.01 | ILMN_2664224 |
| Epn2 | -1.6 | -1.8 | 0.03 | ILMN_1244006 |
| Erdr1 | 1.2 | 1.7 | 0.01 | ILMN_2998658 |
| Ergic1 | 1.4 | 2.1 | 0.02 | ILMN_2745433 |
| Esd | 1.3 | 1.8 | 0.02 | ILMN_1219686 |
| Etl4 | -1.4 | -1.7 | 0.02 | ILMN_1259022 |
| Ext1 | 1.3 | 1.5 | 0.02 | ILMN_2731989 |
| F13a1 | 3.0 | 2.0 | 0.04 | ILMN_2914938 |
| F8a | -1.3 | -1.6 | 0.01 | ILMN_2622041 |
| Faim2 | -1.5 | -1.8 | 0.01 | ILMN_3136149 |
| Fblim1 | 1.3 | 2.1 | 0.00 | ILMN_2837671 |
| Fbn1 | 1.6 | 3.2 | 0.00 | ILMN_1223552 |
| Fbxo2 | 1.2 | 1.5 | 0.03 | ILMN_2822825 |
| Fcgr3 | -1.1 | -1.5 | 0.02 | ILMN_2687403 |
| Fez1 | 1.5 | -1.1 | 0.00 | ILMN_1213056 |
| Fhl1 | -1.3 | -1.5 | 0.02 | ILMN_3117381 |
| Fkbp1a | 1.2 | 1.7 | 0.03 | ILMN_2776025 |
| Fkhl18 | 1.2 | 1.5 | 0.03 | ILMN_2935157 |
| Flna | 1.6 | 2.6 | 0.02 | ILMN_1245994 |
| Gab1 | -1.3 | -1.7 | 0.04 | ILMN_1222009 |
| Gamt | -1.0 | -1.6 | 0.04 | ILMN_2815506 |
| Gch1 | 1.1 | 1.6 | 0.01 | ILMN_2606295 |
| Gdf15 | 1.1 | 2.5 | 0.02 | ILMN_2735350 |
| Gjb2 | 3.5 | 2.0 | 0.01 | ILMN_2999627 |
| Glt25d2 | -1.4 | -2.1 | 0.03 | ILMN_1257051 |
| Gm347 | -1.5 | -1.8 | 0.01 | ILMN_2911307 |
| Gmpr | -1.2 | -1.9 | 0.01 | ILMN_2602581 |
| Golga2 | 1.3 | 1.6 | 0.02 | ILMN_3137432 |
| Gorasp1 | -1.3 | -1.7 | 0.02 | ILMN_2710103 |
| Gpr155 | -1.1 | -1.6 | 0.04 | ILMN_1230183 |
| Gpr155 | -1.1 | -2.1 | 0.01 | ILMN_1249003 |
| Gpt1 | -1.4 | -1.8 | 0.01 | ILMN_2832524 |
| Grit | -1.4 | -1.9 | 0.03 | ILMN_1225037 |
| Gstt3 | -1.5 | -1.2 | 0.03 | ILMN_2665715 |
| H2afv | -1.3 | -1.9 | 0.02 | ILMN_2703385 |
| H2-K1 | -1.8 | -1.3 | 0.02 | ILMN_2588139 |
| Hbb-b1 | -1.1 | -1.5 | 0.03 | ILMN_1235372 |
| Hectd3 | -1.2 | -1.6 | 0.02 | ILMN_1231383 |
| Hist1h2ah | 2.2 | 1.3 | 0.02 | ILMN_1246108 |
| Hist1h2be | -1.3 | -2.2 | 0.01 | ILMN_2676243 |
| Hist1h4j | -1.2 | -1.5 | 0.01 | ILMN_1256989 |
| Hist2h2aa2 | -1.4 | -1.7 | 0.03 | ILMN_1242167 |
| Hnrpdl | 1.1 | 1.8 | 0.01 | ILMN_2903440 |
| Hoxa7 | 1.5 | 1.1 | 0.02 | ILMN_2621038 |
| Hs2st1 | -1.6 | -2.1 | 0.01 | ILMN_2777202 |
| Hsd17b12 | 1.5 | 1.9 | 0.03 | ILMN_2689908 |
| Hsd17b7 | -1.2 | 1.8 | 0.00 | ILMN_1229529 |
| Hspa2 | -1.1 | -1.5 | 0.02 | ILMN_1223285 |
| Hspb6 | 3.3 | 1.8 | 0.02 | ILMN_2805339 |
| Il1b | 3.1 | -1.4 | 0.04 | ILMN_2777498 |
| Impdh2 | 1.6 | 1.4 | 0.01 | ILMN_2588398 |
| Inmt | -1.3 | -2.3 | 0.03 | ILMN_2803249 |
| Iqcb1 | -1.4 | -1.7 | 0.02 | ILMN_2635348 |
| Irx5 | -1.1 | -1.6 | 0.03 | ILMN_2650603 |
| Itgb5 | -1.3 | -1.8 | 0.01 | ILMN_2663613 |
| Kalrn | 1.1 | 1.7 | 0.03 | ILMN_2727847 |
| Kank3 | -1.5 | -1.4 | 0.01 | ILMN_1224692 |
| Kcna6 | -1.2 | -2.1 | 0.02 | ILMN_2760927 |
| Kcna6 | -1.4 | -2.8 | 0.00 | ILMN_2596385 |
| Klc1 | 1.2 | 1.5 | 0.03 | ILMN_2671411 |
| Klk13 | 1.6 | 1.3 | 0.02 | ILMN_3040588 |
| Klk8 | -1.1 | -1.6 | 0.04 | ILMN_2675232 |
| Krt75 | -1.1 | -1.5 | 0.03 | ILMN_2679759 |
| Lad1 | 1.6 | 1.2 | 0.00 | ILMN_1227260 |
| Ldlr | 1.2 | 1.6 | 0.02 | ILMN_1233469 |
| Leo1 | 1.1 | 1.5 | 0.00 | ILMN_2903351 |
| Lgi2 | 1.1 | 1.6 | 0.00 | ILMN_2635700 |
| Litaf | 1.2 | 1.6 | 0.04 | ILMN_2665490 |
| LOC100039496 | -1.6 | -2.2 | 0.04 | ILMN_1238200 |
| LOC100044395 | 1.2 | 1.6 | 0.01 | ILMN_2546905 |
| LOC100045280 | 1.6 | 2.0 | 0.01 | ILMN_1225118 |
| LOC100045359 | 1.2 | 1.6 | 0.02 | ILMN_2694153 |
| LOC100045677 | 1.6 | 1.3 | 0.02 | ILMN_2633377 |
| LOC100046895 | 1.2 | 2.2 | 0.01 | ILMN_2422687 |
| LOC100046963 | -1.1 | -1.8 | 0.04 | ILMN_1220560 |
| LOC100047093 | -1.3 | -1.7 | 0.01 | ILMN_2451036 |
| LOC100047173 | 1.7 | 1.4 | 0.01 | ILMN_2651054 |
| LOC100047214 | -1.4 | -2.0 | 0.00 | ILMN_1255556 |
| LOC100047670 | -1.2 | -1.7 | 0.01 | ILMN_1236937 |
| LOC100048123 | -1.3 | -1.5 | 0.00 | ILMN_1251040 |
| LOC100048169 | 1.7 | 1.3 | 0.04 | ILMN_2430359 |
| LOC277837 | 1.5 | 2.4 | 0.03 | ILMN_1239725 |
| LOC383077 | 1.1 | -1.6 | 0.00 | ILMN_2536687 |
| LOC383576 | -1.1 | -1.6 | 0.04 | ILMN_2535430 |
| LOC384538 | -1.9 | -3.2 | 0.02 | ILMN_2539917 |
| LOC385068 | -1.1 | -1.5 | 0.00 | ILMN_1224842 |
| LOC545396 | -1.2 | -1.5 | 0.03 | ILMN_1234315 |
| LOC674427 | -1.2 | -1.7 | 0.02 | ILMN_1234412 |
| LOC674611 | -1.3 | -1.8 | 0.02 | ILMN_1217442 |
| Lox | 1.9 | 4.5 | 0.02 | ILMN_2997494 |
| Loxl1 | 1.3 | 2.5 | 0.00 | ILMN_1255871 |
| Lrch1 | -1.2 | -1.7 | 0.02 | ILMN_2473718 |
| Lrig3 | -1.1 | -1.5 | 0.00 | ILMN_1213273 |
| Lrrc59 | 1.2 | 1.7 | 0.01 | ILMN_1252817 |
| Lrrc8a | 1.6 | 2.7 | 0.04 | ILMN_2895213 |
| Lrrc8c | 1.3 | 1.6 | 0.02 | ILMN_1248696 |
| Ly6c1 | 1.4 | 2.3 | 0.01 | ILMN_1254927 |
| Ly6g6c | 3.9 | 2.6 | 0.01 | ILMN_1251725 |
| Ly6g6d | -1.1 | -1.6 | 0.01 | ILMN_2599251 |
| Marco | 2.0 | 1.7 | 0.04 | ILMN_1229922 |
| Mcam | 1.2 | 2.5 | 0.03 | ILMN_1229960 |
| Mcam | -1.1 | 1.9 | 0.00 | ILMN_2955919 |
| Mcee | -1.2 | -1.7 | 0.01 | ILMN_2707494 |
| Mcm5 | 1.9 | 1.4 | 0.03 | ILMN_2742849 |
| Mcm6 | 2.6 | 2.0 | 0.01 | ILMN_2803399 |
| Mettl1 | 1.5 | 2.1 | 0.01 | ILMN_2732419 |
| Mettl1 | 1.4 | 2.3 | 0.00 | ILMN_3001946 |
| Mfap1b | -1.3 | -1.6 | 0.03 | ILMN_2802103 |
| Mlkl | 1.4 | 2.0 | 0.02 | ILMN_3006767 |
| Mllt4 | 1.4 | 1.9 | 0.03 | ILMN_2593230 |
| Mlycd | -1.3 | -1.7 | 0.01 | ILMN_1249691 |
| Mmp14 | 1.4 | 2.3 | 0.00 | ILMN_1240726 |
| Mmp3 | 2.1 | 3.3 | 0.03 | ILMN_2753809 |
| Mmp3 | 2.4 | 3.9 | 0.02 | ILMN_2704576 |
| Mpv17 | -1.1 | -1.6 | 0.01 | ILMN_2657857 |
| Mrpl16 | 1.2 | 1.5 | 0.00 | ILMN_2611648 |
| Mrps18b | 1.3 | 1.8 | 0.01 | ILMN_2865239 |
| mtDNA_ND5 | -1.2 | -1.5 | 0.01 | ILMN_2507810 |
| Mybpc2 | 3.2 | 1.6 | 0.00 | ILMN_2676012 |
| Mylpf | 15.5 | 6.3 | 0.01 | ILMN_2745496 |
| Myom3 | 1.5 | -1.3 | 0.00 | ILMN_2673358 |
| Naprt1 | 1.5 | 1.2 | 0.04 | ILMN_2799590 |
| Nav1 | -1.1 | 1.6 | 0.00 | ILMN_2883016 |
| Ncf1 | -1.1 | -1.5 | 0.00 | ILMN_2829457 |
| Nckap1 | 1.4 | 1.9 | 0.04 | ILMN_1220664 |
| Nde1 | -1.5 | -1.1 | 0.00 | ILMN_2690077 |
| Ndn | 1.2 | 1.5 | 0.02 | ILMN_2622374 |
| Nes | -1.2 | 1.7 | 0.00 | ILMN_2703267 |
| Nfia | -1.2 | -1.5 | 0.00 | ILMN_2736379 |
| Ngef | -1.2 | -1.9 | 0.00 | ILMN_1245037 |
| Nid1 | 1.1 | 1.8 | 0.02 | ILMN_2814005 |
| Nme2 | 1.3 | 1.6 | 0.04 | ILMN_2705935 |
| Nol5 | 2.0 | 2.6 | 0.04 | ILMN_1235979 |
| Nol8 | -1.5 | -1.2 | 0.03 | ILMN_2599478 |
| Nphp4 | -1.2 | -1.5 | 0.04 | ILMN_2641152 |
| Npm1 | 1.6 | 1.4 | 0.01 | ILMN_2970473 |
| Nub1 | -1.3 | -1.6 | 0.04 | ILMN_2765080 |
| Oaz2 | -1.3 | -1.6 | 0.02 | ILMN_2683794 |
| Ociad2 | -1.8 | -2.3 | 0.01 | ILMN_2482178 |
| Ociad2 | -1.4 | -2.0 | 0.00 | ILMN_2943722 |
| Ogn | -1.6 | -2.5 | 0.02 | ILMN_2859613 |
| ORF28 | -1.2 | -1.8 | 0.00 | ILMN_1219898 |
| Osbpl6 | -1.5 | -2.0 | 0.00 | ILMN_1221789 |
| OTTMUSG00000000971 | 4.2 | 1.7 | 0.01 | ILMN_2864309 |
| P2ry14 | -1.1 | -1.6 | 0.00 | ILMN_3154419 |
| P4ha2 | -1.6 | -1.2 | 0.03 | ILMN_2777082 |
| Pcdh1 | 1.4 | 2.1 | 0.03 | ILMN_2597660 |
| Pcdh1 | 1.6 | 2.3 | 0.00 | ILMN_1260020 |
| Pcdha7 | -1.7 | -2.5 | 0.00 | ILMN_1215440 |
| Pcdhac2 | -1.1 | -1.6 | 0.00 | ILMN_2999354 |
| Pcmtd2 | -1.2 | -1.6 | 0.03 | ILMN_2647084 |
| Pcp4l1 | -1.4 | -2.1 | 0.00 | ILMN_1254622 |
| Pcp4l1 | -2.3 | -3.8 | 0.00 | ILMN_1218127 |
| Pdcd6ip | 1.2 | 1.6 | 0.01 | ILMN_1216394 |
| Pdlim2 | 1.2 | 1.7 | 0.01 | ILMN_2880906 |
| Pdss1 | 1.4 | 1.6 | 0.03 | ILMN_2986605 |
| Pdzd2 | -1.7 | -2.1 | 0.04 | ILMN_1231978 |
| Pdzk1ip1 | -1.2 | -1.5 | 0.04 | ILMN_2618935 |
| Pex11a | -1.6 | -1.1 | 0.03 | ILMN_2700265 |
| Pfdn5 | -1.4 | -1.7 | 0.02 | ILMN_2671747 |
| Pfdn5 | -1.5 | -1.9 | 0.01 | ILMN_2766262 |
| Pfkm | 1.6 | 1.1 | 0.00 | ILMN_2628892 |
| Pgam2 | 3.7 | 0.8 | 0.04 | ILMN_2588815 |
| Pglyrp1 | 2.3 | 1.6 | 0.02 | ILMN_2592486 |
| Phca | 1.1 | 1.5 | 0.01 | ILMN_2681057 |
| Phldb1 | 1.3 | 1.8 | 0.04 | ILMN_2884126 |
| Pitrm1 | 1.3 | 1.6 | 0.03 | ILMN_2780167 |
| Pkp2 | -1.2 | -1.6 | 0.02 | ILMN_2927565 |
| Pla2g1b | -1.2 | -1.7 | 0.00 | ILMN_2845906 |
| Plat | 1.4 | 5.7 | 0.03 | ILMN_2775937 |
| Plau | 1.2 | 1.7 | 0.03 | ILMN_2629582 |
| Plaur | -1.1 | 1.7 | 0.00 | ILMN_2590884 |
| Plekhm3 | -1.1 | -1.5 | 0.02 | ILMN_2482672 |
| Plscr1 | 1.1 | 1.5 | 0.01 | ILMN_2911344 |
| Plscr4 | -1.6 | -2.6 | 0.03 | ILMN_2758717 |
| Plxna2 | -1.5 | 1.8 | 0.00 | ILMN_2701815 |
| Pno1 | 1.9 | 2.8 | 0.02 | ILMN_1233889 |
| Poli | -1.2 | -1.5 | 0.02 | ILMN_2614540 |
| Pon1 | -1.5 | -2.4 | 0.00 | ILMN_2676379 |
| Ppap2a | 1.1 | 1.9 | 0.04 | ILMN_1222991 |
| Ppap2a | 1.3 | 2.2 | 0.01 | ILMN_2759079 |
| Ppm1b | -1.2 | -1.5 | 0.04 | ILMN_2520446 |
| Ppm1f | -1.6 | -1.1 | 0.03 | ILMN_1221199 |
| Ppp1r14b | 2.2 | 2.7 | 0.03 | ILMN_1242221 |
| Ppp1r14c | -1.3 | -1.7 | 0.04 | ILMN_2632509 |
| Ppp1r1b | -2.2 | -1.8 | 0.02 | ILMN_2954824 |
| Ppp1r9a | -1.3 | -1.6 | 0.03 | ILMN_2632092 |
| Pps | -1.6 | -2.6 | 0.00 | ILMN_1248389 |
| Prdx6 | -1.6 | -1.3 | 0.02 | ILMN_2623947 |
| Prkcbp1 | 1.5 | 1.2 | 0.02 | ILMN_2627205 |
| Prkcdbp | 1.8 | 2.3 | 0.03 | ILMN_2956942 |
| Prkcdbp | 1.4 | 2.0 | 0.00 | ILMN_1243602 |
| Prkcz | -1.8 | -2.2 | 0.03 | ILMN_3158919 |
| Psip1 | -1.2 | -1.5 | 0.01 | ILMN_2617478 |
| Pskh1 | -1.8 | -2.1 | 0.01 | ILMN_2713872 |
| Psma4 | 1.5 | 1.3 | 0.02 | ILMN_2771944 |
| Psmb1 | 1.7 | 2.3 | 0.01 | ILMN_1239724 |
| Psmb5 | 1.6 | 2.1 | 0.03 | ILMN_2613469 |
| Psmd11 | 1.2 | 1.7 | 0.03 | ILMN_2888116 |
| Psmf1 | -1.3 | -1.5 | 0.01 | ILMN_1233929 |
| Ptges | 1.3 | 1.8 | 0.02 | ILMN_2786442 |
| Ptpdc1 | -1.4 | -1.7 | 0.04 | ILMN_2701543 |
| Ptpla | 1.4 | 1.8 | 0.02 | ILMN_2734712 |
| Ptprg | -1.1 | 1.6 | 0.00 | ILMN_2572307 |
| Ptpru | -1.2 | -1.7 | 0.02 | ILMN_2850253 |
| Pvr | -1.2 | 2.2 | 0.00 | ILMN_2972585 |
| Rab19 | -1.3 | -1.5 | 0.03 | ILMN_2942082 |
| Rab6b | -1.9 | -2.4 | 0.02 | ILMN_2761430 |
| Rabep2 | -1.7 | -1.4 | 0.04 | ILMN_2599751 |
| Rac3 | -1.3 | -2.1 | 0.02 | ILMN_2696749 |
| Rac3 | -1.3 | -1.9 | 0.01 | ILMN_1218393 |
| Rage | -1.8 | -1.4 | 0.04 | ILMN_2755243 |
| Ralgps1 | -1.3 | -1.5 | 0.02 | ILMN_1230211 |
| Ran | 1.3 | 2.1 | 0.00 | ILMN_2934457 |
| Rapgefl1 | 1.7 | 1.4 | 0.03 | ILMN_1250569 |
| Rars | 1.5 | 1.7 | 0.01 | ILMN_2907642 |
| Rasd2 | -1.2 | -1.9 | 0.00 | ILMN_1223875 |
| Rassf4 | -1.3 | -1.8 | 0.01 | ILMN_2956092 |
| Rbbp9 | -1.2 | -1.6 | 0.04 | ILMN_1214213 |
| Rbbp9 | -1.3 | -1.5 | 0.03 | ILMN_2890496 |
| Rbm47 | 1.5 | 2.0 | 0.02 | ILMN_2720836 |
| Rbm4b | -1.3 | -1.7 | 0.03 | ILMN_1220793 |
| Rbms1 | 1.2 | 2.0 | 0.03 | ILMN_2883990 |
| Rbpjl | -2.2 | -1.8 | 0.04 | ILMN_2733957 |
| Rcan2 | -1.7 | -2.1 | 0.04 | ILMN_3106592 |
| Rdh12 | 2.1 | 1.5 | 0.03 | ILMN_2836501 |
| Rem2 | -1.4 | -1.6 | 0.01 | ILMN_1222084 |
| Retnla | 4.3 | 7.8 | 0.00 | ILMN_1226472 |
| Rfx1 | -1.4 | -1.7 | 0.00 | ILMN_2832620 |
| Rgs3 | -1.0 | -1.5 | 0.02 | ILMN_1241709 |
| Rgs7bp | -1.1 | -1.5 | 0.04 | ILMN_2733314 |
| Rhebl1 | -1.2 | -1.5 | 0.04 | ILMN_2591469 |
| Rhoc | 1.2 | 2.6 | 0.04 | ILMN_2749437 |
| Rhoc | 1.5 | 2.7 | 0.03 | ILMN_2790097 |
| Rhoj | 1.1 | 1.5 | 0.00 | ILMN_2653567 |
| Rnase4 | -1.2 | -1.7 | 0.01 | ILMN_2644504 |
| Rnf135 | -1.4 | -1.5 | 0.01 | ILMN_2820379 |
| Rnpepl1 | -1.3 | -1.6 | 0.04 | ILMN_2666190 |
| Rpl3l | 1.9 | -1.2 | 0.00 | ILMN_2649810 |
| Rpp25 | 3.8 | 2.7 | 0.01 | ILMN_2754222 |
| Rras2 | -0.6 | 1.8 | 0.04 | ILMN_2909782 |
| Rrbp1 | 1.5 | 2.0 | 0.02 | ILMN_2597686 |
| Rrbp1 | 1.9 | 2.5 | 0.00 | ILMN_2612079 |
| Rsc1a1 | 1.1 | 1.5 | 0.03 | ILMN_1230668 |
| Rtkn2 | -1.4 | -1.9 | 0.01 | ILMN_1224207 |
| Ryr1 | 2.3 | 1.2 | 0.01 | ILMN_2730425 |
| Ryr1 | 2.9 | 1.4 | 0.00 | ILMN_2733073 |
| S100a10 | 1.2 | 1.8 | 0.01 | ILMN_1256702 |
| S100a8 | 4.5 | 0.4 | 0.04 | ILMN_2710905 |
| S100a9 | 5.0 | 0.5 | 0.02 | ILMN_2803674 |
| Scgb1c1 | -2.1 | -2.7 | 0.04 | ILMN_2609421 |
| scl0002069.1_48 | -1.2 | -1.8 | 0.00 | ILMN_2443164 |
| Scly | 1.3 | 1.6 | 0.04 | ILMN_2564539 |
| Sdcbp2 | 1.4 | 2.4 | 0.02 | ILMN_2825446 |
| Sdf4 | -1.2 | -1.8 | 0.03 | ILMN_2726315 |
| Secisbp2 | 1.4 | 1.7 | 0.01 | ILMN_2657888 |
| Sema7a | -0.4 | 3.3 | 0.02 | ILMN_2790357 |
| Sepp1 | -1.9 | -2.6 | 0.03 | ILMN_3141048 |
| Serpina3g | -1.0 | 1.5 | 0.00 | ILMN_2725927 |
| Sertad1 | 1.5 | 2.0 | 0.04 | ILMN_2729162 |
| Sertad2 | -1.2 | -1.7 | 0.04 | ILMN_1231586 |
| Sfxn4 | -1.3 | -1.8 | 0.02 | ILMN_2776377 |
| Sh3gl2 | -1.3 | -2.1 | 0.00 | ILMN_1251416 |
| Sh3tc1 | -1.6 | -1.2 | 0.02 | ILMN_2847906 |
| Sharpin | -1.5 | -1.3 | 0.03 | ILMN_1254001 |
| Shroom2 | -1.5 | -1.2 | 0.04 | ILMN_1248608 |
| Skiv2l2 | -1.3 | -1.9 | 0.02 | ILMN_1218617 |
| Slc16a9 | -1.7 | -2.6 | 0.01 | ILMN_2922560 |
| Slc1a1 | -1.4 | -1.9 | 0.02 | ILMN_1229397 |
| Slc25a3 | 1.3 | 1.8 | 0.03 | ILMN_2751046 |
| Slc35f3 | -1.3 | -1.6 | 0.03 | ILMN_2698880 |
| Slc39a13 | 1.4 | 1.7 | 0.03 | ILMN_2996732 |
| Slpi | 5.0 | 2.7 | 0.02 | ILMN_1256817 |
| Smad1 | -1.6 | -1.2 | 0.02 | ILMN_2953700 |
| Smarcb1 | 1.2 | 1.5 | 0.01 | ILMN_1254840 |
| Smarcd3 | 1.6 | 1.3 | 0.03 | ILMN_1248397 |
| Smg6 | -1.5 | -1.9 | 0.03 | ILMN_3160556 |
| Snca | -1.2 | -2.2 | 0.02 | ILMN_3161601 |
| Sort1 | -1.3 | -1.6 | 0.00 | ILMN_1219717 |
| Spnb1 | 1.2 | -1.8 | 0.00 | ILMN_2909238 |
| Spon2 | 1.6 | 2.9 | 0.04 | ILMN_1229547 |
| Sri | 1.3 | 1.5 | 0.03 | ILMN_3120014 |
| Sspn | -1.1 | -2.0 | 0.00 | ILMN_2741402 |
| Sstr2 | -1.2 | -1.6 | 0.03 | ILMN_1229577 |
| Stk17b | -1.3 | -1.7 | 0.03 | ILMN_1255834 |
| Stk39 | 1.2 | 1.7 | 0.00 | ILMN_2751494 |
| Syf2 | -1.3 | -1.8 | 0.02 | ILMN_2956381 |
| Tagln | 1.3 | 2.4 | 0.01 | ILMN_1243652 |
| Tcap | 1.7 | -1.6 | 0.00 | ILMN_2696682 |
| Tcea3 | -1.3 | -1.9 | 0.01 | ILMN_2776283 |
| Tff2 | -1.3 | -1.9 | 0.04 | ILMN_1228211 |
| Tgfb3 | 1.1 | 1.7 | 0.02 | ILMN_2748966 |
| Thoc6 | 1.5 | 1.9 | 0.00 | ILMN_2778428 |
| Thyn1 | 1.4 | 1.6 | 0.00 | ILMN_2987062 |
| Tinag | -1.6 | -2.4 | 0.00 | ILMN_1248959 |
| Tinagl | -0.4 | 2.8 | 0.04 | ILMN_2760619 |
| Tinagl | 0.7 | 4.6 | 0.03 | ILMN_2976129 |
| Tmem134 | -1.5 | -1.1 | 0.02 | ILMN_3125890 |
| Tmem82 | -0.4 | 1.9 | 0.03 | ILMN_2666312 |
| Tmem87b | 1.1 | 1.5 | 0.03 | ILMN_1235007 |
| Tmod4 | 1.5 | -1.1 | 0.00 | ILMN_2488846 |
| Tnc | 2.5 | 6.7 | 0.03 | ILMN_2463180 |
| Tnfrsf12a | 1.5 | 4.8 | 0.04 | ILMN_2424299 |
| Tnfsf12-tnfsf13 | -1.3 | -1.7 | 0.03 | ILMN_2492170 |
| Tnni2 | 23.2 | 12.7 | 0.03 | ILMN_2481133 |
| Tomm70a | -1.6 | -1.3 | 0.02 | ILMN_1234112 |
| Tppp | 1.2 | -1.5 | 0.00 | ILMN_2696110 |
| Tppp3 | -1.4 | -1.5 | 0.01 | ILMN_2655929 |
| Traf1 | -1.4 | -2.1 | 0.02 | ILMN_1253947 |
| Trappc6b | 1.0 | 1.5 | 0.00 | ILMN_2485266 |
| Trem3 | 1.2 | -1.5 | 0.00 | ILMN_2915303 |
| Trim72 | 4.6 | 2.3 | 0.02 | ILMN_2900484 |
| Trip6 | 1.3 | 1.8 | 0.02 | ILMN_3009463 |
| Trp53 | 1.1 | 1.6 | 0.00 | ILMN_2466845 |
| Tsg101 | 1.3 | 1.9 | 0.00 | ILMN_2452237 |
| Tspan17 | -1.3 | -1.6 | 0.00 | ILMN_2817714 |
| Ttc3 | -1.2 | -1.6 | 0.04 | ILMN_2841721 |
| Ttc3 | -1.3 | -1.8 | 0.01 | ILMN_2498479 |
| Ttn | 5.3 | 2.4 | 0.04 | ILMN_2416670 |
| Ttn | 5.3 | 2.3 | 0.02 | ILMN_3128792 |
| Ttn | 4.0 | 1.6 | 0.00 | ILMN_1223811 |
| Tuba1a | -1.5 | 1.1 | 0.00 | ILMN_2494251 |
| Tuba1b | 1.3 | 1.5 | 0.02 | ILMN_2986393 |
| Tuba6 | 1.5 | 2.2 | 0.02 | ILMN_2476139 |
| Tubb2b | 1.4 | 2.7 | 0.01 | ILMN_2588051 |
| Tubb6 | 1.8 | 3.4 | 0.04 | ILMN_2718217 |
| Tut1 | -1.5 | -1.3 | 0.01 | ILMN_1232593 |
| Txnl4b | -1.3 | -1.5 | 0.03 | ILMN_1257251 |
| Txnrd2 | -1.4 | -1.8 | 0.02 | ILMN_2424605 |
| Ube2f | 1.1 | 1.5 | 0.02 | ILMN_2614522 |
| Ube2f | 1.2 | 1.5 | 0.02 | ILMN_2835443 |
| Ube2h | -1.2 | -1.6 | 0.01 | ILMN_2451035 |
| Upp1 | -0.3 | 2.0 | 0.04 | ILMN_2959291 |
| Upp1 | -0.4 | 1.9 | 0.04 | ILMN_2959292 |
| Vars | 1.1 | 1.7 | 0.03 | ILMN_2928160 |
| Vgll3 | 1.3 | 2.0 | 0.01 | ILMN_1220029 |
| Vill | 1.1 | 1.5 | 0.03 | ILMN_2846432 |
| Vpreb3 | -1.5 | -3.0 | 0.01 | ILMN_2469253 |
| Vsig2 | -1.5 | -1.1 | 0.01 | ILMN_1245518 |
| Vsnl1 | -1.6 | -2.2 | 0.04 | ILMN_2438724 |
| Zdhhc14 | -1.1 | -1.5 | 0.01 | ILMN_1240332 |
| Zer1 | -1.3 | -1.7 | 0.03 | ILMN_1227218 |
| Zfand2a | -1.6 | -1.3 | 0.02 | ILMN_1230489 |
| Zfp750 | 1.8 | 1.4 | 0.03 | ILMN_2654571 |
| Zhx1 | -1.2 | -1.8 | 0.03 | ILMN_2510714 |
| Zhx3 | -1.9 | -1.2 | 0.00 | ILMN_2502623 |
