# Supplementary material for: Age-Dependent Decline in Mouse Lung Regeneration with Loss of Lung Fibroblast Clonogenicity and Increased Myofibroblastic Differentiation
Source: PLoS One. 2011 Aug 30;6(8):e23232. doi: 10.1371/journal.pone.0023232 (PMC3166052; doi:10.1371/journal.pone.0023232)
Supplement: Table S3 — Microarray analysis of genes with significant differences (P<0.05) in differential regulation (fold change, compared to pre-PNX samples) between 9 and 3 month mice 3 days after PNX. (DOC) [file pone.0023232.s003.doc]

**Table S3** – Microarray analysis of genes with significant differences (P<0.05) in differential regulation (fold change, compared to pre-PNX samples) between 9 and 3 month mice 3 days after PNX

| SYMBOL | 3 mo FC | 9 mo FC | P | PROBE_ID |
| --- | --- | --- | --- | --- |
| 1110008F13Rik | 1.2 | 1.5 | 0.00 | ILMN_1252407 |
| 1190002A17Rik | -1.2 | -1.7 | 0.04 | ILMN_2590894 |
| 1190002H23Rik | -2.3 | -1.6 | 0.01 | ILMN_2593774 |
| 1700003E16Rik | -1.4 | -2.0 | 0.01 | ILMN_1231608 |
| 1700109H08Rik | -1.7 | -2.1 | 0.03 | ILMN_1225045 |
| 2210408F11Rik | -1.2 | -1.8 | 0.03 | ILMN_2483811 |
| 2510042H12Rik | -2.2 | -7.7 | 0.04 | ILMN_1241039 |
| 2700084L06Rik | 1.2 | 1.8 | 0.00 | ILMN_2439313 |
| 2810417H13Rik | 2.7 | 3.8 | 0.04 | ILMN_1223045 |
| 2810453I06Rik | -1.9 | -3.1 | 0.03 | ILMN_2510771 |
| 2810453I06Rik | -1.5 | -2.5 | 0.00 | ILMN_2695228 |
| 3526401B18Rik | -1.3 | -1.6 | 0.02 | ILMN_2440634 |
| 4432416J03Rik | -1.3 | -1.5 | 0.03 | ILMN_2931918 |
| 4931408A02Rik | -1.5 | -1.2 | 0.04 | ILMN_2949877 |
| 4933404M02Rik | -1.6 | -2.4 | 0.00 | ILMN_1249756 |
| 4933417E08Rik | -1.7 | -1.3 | 0.02 | ILMN_2584195 |
| 5031436O03Rik | -1.6 | -2.1 | 0.03 | ILMN_1219017 |
| 5430417L22Rik | -1.7 | -1.4 | 0.04 | ILMN_1226555 |
| 5630401D06Rik | -1.5 | -1.7 | 0.02 | ILMN_2468405 |
| 5830467P10Rik | -1.2 | -1.8 | 0.00 | ILMN_1234099 |
| 6330407I18Rik | -1.2 | -1.6 | 0.01 | ILMN_2489147 |
| 6430537H07Rik | -1.6 | -2.2 | 0.02 | ILMN_2720911 |
| A130092J06Rik | -1.4 | -1.8 | 0.02 | ILMN_2601453 |
| A330068P14Rik | -1.2 | -1.5 | 0.03 | ILMN_2482949 |
| A730017D01Rik | -1.3 | -1.7 | 0.02 | ILMN_2448591 |
| A830087P12Rik | -1.7 | -2.1 | 0.02 | ILMN_1236899 |
| A930019L04Rik | -1.5 | -1.1 | 0.01 | ILMN_2458545 |
| Adamtsl4 | -1.6 | -1.2 | 0.01 | ILMN_2459899 |
| Adssl1 | 1.6 | 1.2 | 0.04 | ILMN_2958099 |
| Akap8l | -1.6 | -2.1 | 0.04 | ILMN_1242769 |
| Alas2 | -1.6 | -4.5 | 0.04 | ILMN_2675874 |
| Amotl2 | -1.5 | -1.9 | 0.02 | ILMN_2774049 |
| Anapc5 | -1.2 | -1.5 | 0.04 | ILMN_3158200 |
| Armc10 | 1.6 | 1.2 | 0.01 | ILMN_2757916 |
| Armc9 | -1.3 | -1.5 | 0.00 | ILMN_2695415 |
| Asah3l | -1.5 | -1.3 | 0.03 | ILMN_2629112 |
| Asb13 | -1.4 | -1.6 | 0.03 | ILMN_3104025 |
| AU018778 | -1.5 | -2.8 | 0.03 | ILMN_1238140 |
| B130007O15Rik | -1.5 | -1.2 | 0.04 | ILMN_1214795 |
| B230112P13Rik | -1.5 | -1.2 | 0.04 | ILMN_2433119 |
| B930045J24Rik | -1.2 | -1.6 | 0.03 | ILMN_2567349 |
| BC018465 | -1.2 | -1.6 | 0.03 | ILMN_1227845 |
| BC024561 | -1.5 | 1.2 | 0.00 | ILMN_2783414 |
| BC050811 | -1.2 | -1.5 | 0.04 | ILMN_2782841 |
| BC063749 | -1.5 | -1.6 | 0.01 | ILMN_2995794 |
| Bcl7a | -1.4 | -1.9 | 0.00 | ILMN_1252601 |
| Bcl9 | 1.5 | 1.2 | 0.02 | ILMN_2600255 |
| Bcl9l | 1.8 | 1.3 | 0.01 | ILMN_1250469 |
| Brp17 | -1.1 | -2.0 | 0.03 | ILMN_2731550 |
| Brwd1 | -1.3 | -1.5 | 0.02 | ILMN_2834573 |
| C1qb | 2.4 | 1.8 | 0.00 | ILMN_2619620 |
| C2 | 2.0 | 1.5 | 0.03 | ILMN_2612895 |
| C920027I18Rik | -1.2 | 1.5 | 0.00 | ILMN_2483771 |
| Cadps2 | -1.2 | -1.6 | 0.01 | ILMN_2998313 |
| Ccdc3 | 1.6 | -1.1 | 0.00 | ILMN_2611180 |
| Ccdc65 | -1.4 | -1.7 | 0.04 | ILMN_2595469 |
| Ccl5 | -1.3 | -2.4 | 0.04 | ILMN_1231814 |
| Ccng1 | 1.5 | 1.3 | 0.04 | ILMN_2500276 |
| Ccng1 | 1.3 | 1.8 | 0.01 | ILMN_2710229 |
| Cct8 | 1.2 | 1.5 | 0.02 | ILMN_1259677 |
| Cd27 | -1.5 | -2.0 | 0.00 | ILMN_3128992 |
| Cd274 | -1.6 | -2.4 | 0.02 | ILMN_1226800 |
| Cd79b | 1.3 | -1.5 | 0.00 | ILMN_2610822 |
| Cd8b1 | -1.3 | -1.9 | 0.00 | ILMN_2664929 |
| Cd93 | 1.1 | 1.6 | 0.00 | ILMN_1226829 |
| Cd97 | -1.3 | -1.7 | 0.01 | ILMN_2814168 |
| Ceacam1 | -2.5 | -1.6 | 0.04 | ILMN_3126609 |
| Ceacam2 | 1.2 | 1.5 | 0.03 | ILMN_1239472 |
| Cenpq | 1.2 | 1.8 | 0.02 | ILMN_2761273 |
| Centa2 | -1.8 | -1.5 | 0.02 | ILMN_2589256 |
| Chi3l3 | 2.0 | 1.5 | 0.02 | ILMN_2712986 |
| Chi3l3 | 2.1 | 1.5 | 0.02 | ILMN_3117876 |
| Chia | 2.0 | -0.6 | 0.04 | ILMN_2955940 |
| Chst1 | -1.6 | -2.0 | 0.03 | ILMN_1255385 |
| Chst12 | 1.3 | 1.8 | 0.02 | ILMN_2710205 |
| Cidea | 2.8 | -0.4 | 0.02 | ILMN_1215446 |
| Clic6 | -1.5 | -1.7 | 0.03 | ILMN_2667635 |
| Clip4 | 1.5 | 2.2 | 0.03 | ILMN_2606470 |
| Cnot2 | -1.2 | -1.5 | 0.02 | ILMN_3067780 |
| Col14a1 | 1.7 | 1.4 | 0.00 | ILMN_1229714 |
| Col4a4 | 2.1 | 1.6 | 0.02 | ILMN_2679871 |
| Cox8b | 2.1 | -1.3 | 0.00 | ILMN_2727520 |
| Cp | 1.5 | 2.0 | 0.01 | ILMN_3083163 |
| Csnk1g2 | -1.3 | -1.6 | 0.03 | ILMN_2718251 |
| Csrp3 | 2.1 | -1.0 | 0.01 | ILMN_2789650 |
| Cxcr3 | -1.1 | -1.5 | 0.04 | ILMN_2658786 |
| Cxx1a | 1.3 | 2.9 | 0.01 | ILMN_2952779 |
| Cyp27a1 | -1.3 | -2.0 | 0.03 | ILMN_2960114 |
| Cyp2d22 | -1.3 | -1.6 | 0.01 | ILMN_1214498 |
| D130084M03Rik | -1.2 | -1.6 | 0.00 | ILMN_2680371 |
| Dapk2 | -1.5 | -1.2 | 0.02 | ILMN_2669333 |
| Dapk2 | -1.2 | -1.6 | 0.01 | ILMN_2977558 |
| Dbf4 | 2.0 | 2.8 | 0.03 | ILMN_2952661 |
| Dmkn | 3.6 | 1.7 | 0.03 | ILMN_3105563 |
| Dok2 | 1.9 | 1.3 | 0.01 | ILMN_3160659 |
| Dok3 | -1.4 | -2.0 | 0.01 | ILMN_1258357 |
| E030019B06Rik | -1.3 | -1.7 | 0.02 | ILMN_2751796 |
| Ear2 | -1.2 | -1.5 | 0.04 | ILMN_2731735 |
| EG243642 | 1.3 | 1.7 | 0.03 | ILMN_2534332 |
| EG244911 | -1.5 | -2.3 | 0.04 | ILMN_1235361 |
| EG626367 | 1.2 | 1.6 | 0.01 | ILMN_1237565 |
| Eltd1 | -1.6 | -1.2 | 0.01 | ILMN_1224540 |
| Emilin2 | 3.0 | 2.1 | 0.04 | ILMN_2981363 |
| Emp2 | -1.3 | -1.7 | 0.04 | ILMN_2598103 |
| Eraf | 2.5 | -1.2 | 0.01 | ILMN_2619200 |
| F7 | 1.6 | 1.3 | 0.02 | ILMN_2708871 |
| Fgf10 | -1.3 | -1.5 | 0.04 | ILMN_2982771 |
| Fhl1 | -1.3 | -1.6 | 0.01 | ILMN_3117381 |
| Fkbp5 | -1.6 | 1.8 | 0.00 | ILMN_2718266 |
| Fmo1 | -1.9 | -1.3 | 0.03 | ILMN_2562131 |
| Frag1 | 1.1 | 1.5 | 0.04 | ILMN_2665054 |
| Gab1 | -1.2 | -1.7 | 0.00 | ILMN_1222009 |
| Gab3 | -1.3 | -1.5 | 0.02 | ILMN_1226514 |
| Gabarap | 1.6 | 1.4 | 0.02 | ILMN_1251333 |
| Galntl2 | -1.6 | -1.2 | 0.01 | ILMN_1239386 |
| Gcnt1 | 1.7 | 1.3 | 0.00 | ILMN_1256299 |
| Ggnbp1 | -1.5 | -1.8 | 0.03 | ILMN_2721198 |
| Gja4 | 1.1 | 1.6 | 0.01 | ILMN_2737704 |
| Gle1l | -1.6 | -1.4 | 0.04 | ILMN_2617810 |
| Gpr114 | -1.1 | -1.6 | 0.02 | ILMN_2856926 |
| Gpr116 | -1.7 | -2.0 | 0.03 | ILMN_2597987 |
| Gpr125 | -1.2 | -1.6 | 0.02 | ILMN_1251856 |
| Gpr133 | 2.7 | 2.0 | 0.00 | ILMN_1214189 |
| Gpr182 | -1.6 | -1.1 | 0.03 | ILMN_1213615 |
| Gprasp1 | -1.2 | -2.0 | 0.00 | ILMN_1224770 |
| H2-Oa | -1.3 | -1.7 | 0.03 | ILMN_2773037 |
| H2-Ob | -1.2 | -1.9 | 0.01 | ILMN_2670690 |
| Hexb | 1.6 | 1.3 | 0.02 | ILMN_2829330 |
| Hn1 | 1.5 | 1.9 | 0.04 | ILMN_2758452 |
| Hsf1 | -1.5 | -1.7 | 0.03 | ILMN_1255216 |
| Ide | 1.7 | 1.4 | 0.01 | ILMN_1243499 |
| Igfbp5 | 2.1 | 1.5 | 0.00 | ILMN_2964324 |
| Igfbp5 | 1.9 | 1.4 | 0.00 | ILMN_2682613 |
| Il12a | -1.2 | -1.5 | 0.01 | ILMN_2706462 |
| Ipmk | -1.4 | -1.5 | 0.03 | ILMN_2763825 |
| Itga6 | 1.3 | 2.0 | 0.04 | ILMN_1231492 |
| Itpka | -1.2 | -1.9 | 0.03 | ILMN_2752431 |
| Jak1 | -1.3 | -1.8 | 0.03 | ILMN_1213278 |
| Kank4 | -1.2 | -1.7 | 0.01 | ILMN_2655586 |
| Kcnc4 | -1.4 | -2.2 | 0.02 | ILMN_2771095 |
| Klra4 | -1.3 | -2.2 | 0.03 | ILMN_3031402 |
| Klra7 | -1.2 | -1.9 | 0.00 | ILMN_1227867 |
| Lama3 | -1.2 | -1.6 | 0.00 | ILMN_2752545 |
| Lbh | 1.6 | 1.3 | 0.04 | ILMN_2816180 |
| Lbh | 2.6 | 1.9 | 0.01 | ILMN_1233545 |
| LOC100038894 | -1.5 | -2.3 | 0.03 | ILMN_1252295 |
| LOC100040244 | -1.5 | -1.1 | 0.04 | ILMN_1260579 |
| LOC100044842 | -1.5 | -1.1 | 0.00 | ILMN_2672789 |
| LOC100048295 | 7.0 | 3.9 | 0.02 | ILMN_2470646 |
| LOC100048554 | 3.2 | 2.2 | 0.02 | ILMN_1238886 |
| LOC100048645 | -1.5 | -1.3 | 0.04 | ILMN_2635167 |
| LOC245892 | 1.9 | 1.2 | 0.03 | ILMN_1230065 |
| LOC381215 | 1.1 | 1.5 | 0.03 | ILMN_2531581 |
| LOC381546 | -1.1 | -1.5 | 0.00 | ILMN_1248921 |
| LOC382092 | 1.2 | 1.5 | 0.03 | ILMN_1259528 |
| LOC641366 | 1.4 | 2.0 | 0.00 | ILMN_1224237 |
| LOC675899 | 1.3 | 1.7 | 0.01 | ILMN_1225187 |
| Lsm6 | 1.7 | 1.3 | 0.04 | ILMN_1221336 |
| Ltf | 2.2 | 3.6 | 0.02 | ILMN_2754364 |
| Mapkapk3 | 1.7 | 1.2 | 0.00 | ILMN_2909346 |
| Mbp | -1.2 | -1.5 | 0.01 | ILMN_3011353 |
| Mrgprf | 2.3 | 1.6 | 0.00 | ILMN_2760254 |
| Ms4a7 | 1.6 | 1.3 | 0.00 | ILMN_3091003 |
| Mtmr11 | 1.2 | 1.6 | 0.02 | ILMN_2642922 |
| Muc16 | 3.6 | 2.2 | 0.02 | ILMN_2631869 |
| Mycbpap | -1.5 | -2.0 | 0.04 | ILMN_2820831 |
| Myo1g | -1.6 | -2.2 | 0.04 | ILMN_2810405 |
| Myrip | -2.1 | -1.4 | 0.02 | ILMN_2665441 |
| Nagk | 1.6 | 1.1 | 0.00 | ILMN_2652172 |
| Npc2 | 1.3 | 2.1 | 0.02 | ILMN_2744380 |
| Npr3 | -1.6 | -1.4 | 0.02 | ILMN_2496537 |
| OTTMUSG00000005523 | -1.2 | -1.5 | 0.00 | ILMN_2847773 |
| Pacrg | -1.2 | -1.7 | 0.03 | ILMN_2625279 |
| Pgpep1 | 1.6 | 1.2 | 0.04 | ILMN_2846254 |
| Phex | 1.7 | 1.2 | 0.03 | ILMN_1243830 |
| Pkhd1l1 | 3.0 | 2.1 | 0.04 | ILMN_3000909 |
| Pkp4 | -1.3 | -1.5 | 0.04 | ILMN_1233340 |
| Plekhm3 | -1.3 | -1.5 | 0.04 | ILMN_2482672 |
| Pole3 | 1.4 | 1.6 | 0.03 | ILMN_1254553 |
| Ppl | 1.5 | 1.2 | 0.02 | ILMN_2503188 |
| Prr15 | 3.0 | 2.5 | 0.03 | ILMN_2649456 |
| Pskh1 | -1.3 | -1.7 | 0.04 | ILMN_2713872 |
| Psmc3ip | 1.2 | 1.5 | 0.03 | ILMN_2999654 |
| Psmd11 | 1.2 | 1.5 | 0.02 | ILMN_2888116 |
| Ptpn11 | -1.5 | -1.2 | 0.00 | ILMN_2743723 |
| Rac2 | 1.6 | 1.1 | 0.01 | ILMN_2600678 |
| Rfng | 1.1 | 1.5 | 0.04 | ILMN_1257829 |
| Rgs9 | -1.6 | -2.6 | 0.04 | ILMN_1250689 |
| Rpa2 | 1.3 | 1.5 | 0.03 | ILMN_2602687 |
| Rprml | -1.4 | -2.0 | 0.01 | ILMN_2968147 |
| Scara3 | 2.0 | 1.5 | 0.03 | ILMN_2706268 |
| scl0002507.1_236 | 1.8 | 1.1 | 0.04 | ILMN_2497957 |
| scl0002540.1_6 | -1.2 | -1.5 | 0.02 | ILMN_1254034 |
| Seh1l | 1.3 | 1.5 | 0.02 | ILMN_3096750 |
| Sema3e | -1.3 | -1.6 | 0.03 | ILMN_2720976 |
| Shroom2 | -1.5 | -1.2 | 0.04 | ILMN_1248608 |
| Slc1a1 | -1.2 | -1.6 | 0.00 | ILMN_1225873 |
| Slc25a37 | -1.7 | -2.5 | 0.04 | ILMN_2696610 |
| Snca | -1.6 | -4.3 | 0.04 | ILMN_3161601 |
| Snx10 | 1.5 | 1.3 | 0.00 | ILMN_2609614 |
| Srpx2 | 1.3 | 1.8 | 0.02 | ILMN_2818294 |
| St5 | -1.3 | -1.5 | 0.03 | ILMN_1250665 |
| Stx3 | -1.5 | -1.2 | 0.04 | ILMN_3107114 |
| Tbc1d10c | -1.3 | -2.0 | 0.01 | ILMN_2842338 |
| Tbxas1 | 1.5 | 1.2 | 0.01 | ILMN_1251390 |
| Tekt1 | -1.3 | -1.7 | 0.02 | ILMN_1239718 |
| Tfg | 1.4 | 1.8 | 0.04 | ILMN_2976601 |
| Tlk1 | -1.3 | -1.5 | 0.03 | ILMN_2745450 |
| Tlk2 | -1.1 | -1.5 | 0.01 | ILMN_2940510 |
| Tmem204 | -1.7 | -2.4 | 0.02 | ILMN_3162005 |
| Tmem44 | -1.6 | -1.3 | 0.04 | ILMN_1215524 |
| Tmsb10 | 1.5 | 2.4 | 0.00 | ILMN_1225332 |
| Tpbg | 1.2 | 1.6 | 0.04 | ILMN_1250696 |
| Ube2l6 | -1.5 | -2.6 | 0.03 | ILMN_2486267 |
| Ube2o | -1.2 | -1.8 | 0.00 | ILMN_1245815 |
| Ubr5 | -1.5 | -1.2 | 0.02 | ILMN_1216823 |
| Ubxd1 | -1.2 | -1.5 | 0.03 | ILMN_1234318 |
| Ufsp2 | 1.2 | 1.5 | 0.04 | ILMN_2957898 |
| Upp1 | -1.3 | 1.5 | 0.00 | ILMN_2959291 |
| Usp1 | 1.1 | 1.5 | 0.04 | ILMN_2781170 |
| Vamp4 | -1.9 | -2.3 | 0.03 | ILMN_2516348 |
| Vnn3 | -1.1 | -1.6 | 0.02 | ILMN_1244479 |
| Wbp2 | -1.2 | -1.5 | 0.01 | ILMN_1249698 |
| Xpnpep2 | -1.6 | -2.6 | 0.03 | ILMN_1248998 |
| Xrcc6 | 1.1 | 1.5 | 0.03 | ILMN_1247814 |
| Zbtb16 | -2.9 | -1.5 | 0.01 | ILMN_1229216 |
| Zbtb7c | 1.8 | 1.1 | 0.00 | ILMN_2622500 |
| Zcchc6 | -1.3 | -1.5 | 0.04 | ILMN_1220072 |
| Zfp276 | -1.2 | -1.5 | 0.04 | ILMN_2509962 |
| Zfp326 | 1.0 | 1.5 | 0.04 | ILMN_2481389 |
| Znrf2 | 1.2 | 1.5 | 0.03 | ILMN_1236892 |
