# Supplementary material for: Age-Dependent Decline in Mouse Lung Regeneration with Loss of Lung Fibroblast Clonogenicity and Increased Myofibroblastic Differentiation
Source: PLoS One. 2011 Aug 30;6(8):e23232. doi: 10.1371/journal.pone.0023232 (PMC3166052; doi:10.1371/journal.pone.0023232)
Supplement: Table S4 — Microarray analysis of genes with significant differences (P<0.05) in differential regulation (fold change, compared to pre-PNX samples) between 9 and 3 month mice 7 days after PNX. (DOC) [file pone.0023232.s004.doc]

**Table S4** – Microarray analysis of genes with significant differences (P<0.05) in differential regulation (fold change, compared to pre-PNX samples) between 9 and 3 month mice 7 days after PNX

| SYMBOL | 3 mo FC | 9 mo FC | P | PROBE_ID |
| --- | --- | --- | --- | --- |
| 0610030E20Rik | -1.6 | -1.3 | 0.03 | ILMN_2655105 |
| 1200009O22Rik | -1.4 | -1.6 | 0.04 | ILMN_2615431 |
| 1700023M03Rik | 1.5 | 1.2 | 0.04 | ILMN_2621909 |
| 1810007E14Rik | 1.7 | 2.1 | 0.00 | ILMN_2671528 |
| 1810011H11Rik | 1.3 | 1.8 | 0.01 | ILMN_1222269 |
| 2010001M09Rik | -1.1 | 1.5 | 0.00 | ILMN_2613601 |
| 2210023G05Rik | 1.5 | 1.3 | 0.02 | ILMN_2895284 |
| 2310040A07Rik | -1.5 | -1.2 | 0.04 | ILMN_1237485 |
| 2610307O08Rik | 1.3 | 1.5 | 0.02 | ILMN_2736197 |
| 2810455B08Rik | -1.8 | -1.5 | 0.04 | ILMN_2458130 |
| 2810484G07Rik | 1.4 | -1.5 | 0.00 | ILMN_2467007 |
| 5031439G07Rik | 1.4 | 1.8 | 0.00 | ILMN_1252809 |
| 5330438D12Rik | -1.5 | -1.2 | 0.01 | ILMN_1228920 |
| 5730596K20Rik | 1.7 | 1.5 | 0.02 | ILMN_1241909 |
| 8430426J06Rik | -1.8 | -1.4 | 0.00 | ILMN_2551612 |
| 9630058J23Rik | -1.3 | -1.5 | 0.02 | ILMN_1213026 |
| A230050P20Rik | -1.6 | -1.3 | 0.03 | ILMN_2839745 |
| A730008L03Rik | -1.2 | -1.5 | 0.00 | ILMN_2655803 |
| Abca7 | 2.0 | 1.3 | 0.01 | ILMN_2896639 |
| Acat2 | 1.8 | 1.5 | 0.02 | ILMN_1253008 |
| Adamts2 | 1.8 | 2.3 | 0.02 | ILMN_1226259 |
| Add1 | 1.8 | 1.3 | 0.03 | ILMN_3107228 |
| Adh5 | -1.2 | -1.6 | 0.03 | ILMN_1242178 |
| AK157302 | -1.3 | -1.5 | 0.01 | ILMN_2535680 |
| Ankrd1 | 2.2 | 1.4 | 0.02 | ILMN_2950286 |
| Ankrd37 | 1.2 | 1.7 | 0.01 | ILMN_2595857 |
| Ars2 | 1.6 | 1.5 | 0.02 | ILMN_1225615 |
| Art3 | -1.3 | -1.8 | 0.04 | ILMN_1223147 |
| Atp6v0a2 | -1.5 | -1.2 | 0.02 | ILMN_2747311 |
| Atp6v1c2 | -1.3 | -1.5 | 0.01 | ILMN_2898578 |
| AW112037 | -1.5 | -1.8 | 0.03 | ILMN_1225938 |
| B230112P13Rik | -1.6 | -1.3 | 0.01 | ILMN_2433119 |
| B230386D16Rik | -2.4 | -1.7 | 0.04 | ILMN_2565729 |
| B430109P06Rik | -1.6 | -1.2 | 0.03 | ILMN_1225620 |
| Brwd1 | -1.5 | -1.3 | 0.01 | ILMN_2421220 |
| Bub1b | 2.0 | 2.8 | 0.01 | ILMN_2774007 |
| C1qtnf5 | 1.6 | 1.3 | 0.00 | ILMN_2636764 |
| Cbx7 | -1.4 | -1.8 | 0.03 | ILMN_2947234 |
| Ccdc80 | 3.9 | 5.2 | 0.04 | ILMN_1234824 |
| Ccl7 | 1.5 | 2.1 | 0.03 | ILMN_2771176 |
| Centa1 | 1.6 | 1.4 | 0.03 | ILMN_2538422 |
| Cfp | 1.1 | 1.7 | 0.01 | ILMN_1228320 |
| Cirbp | -1.3 | -1.6 | 0.03 | ILMN_2761594 |
| Cldn15 | 2.6 | 2.4 | 0.01 | ILMN_2674906 |
| Clec4b1 | 1.2 | 1.5 | 0.03 | ILMN_2959372 |
| Cno | 1.6 | 1.3 | 0.01 | ILMN_2591411 |
| Col12a1 | 2.0 | 1.4 | 0.03 | ILMN_2862538 |
| Col1a1 | 4.9 | 7.8 | 0.04 | ILMN_2687872 |
| Col3a1 | 3.4 | 6.4 | 0.01 | ILMN_1258629 |
| Col5a1 | 1.7 | 2.2 | 0.02 | ILMN_2748402 |
| Col6a1 | 1.8 | 2.1 | 0.01 | ILMN_2768087 |
| Copb2 | 1.6 | 1.3 | 0.04 | ILMN_2753196 |
| Creb3l2 | 1.5 | 1.2 | 0.01 | ILMN_2956448 |
| Creg1 | -1.5 | -1.7 | 0.04 | ILMN_1239638 |
| Ctdsp1 | 2.1 | 1.7 | 0.04 | ILMN_1213307 |
| Ctsh | 1.6 | 1.3 | 0.02 | ILMN_2872058 |
| Ctsz | 1.2 | 1.6 | 0.02 | ILMN_2666007 |
| Cyp27a1 | -2.1 | -1.6 | 0.03 | ILMN_2960114 |
| Cyp2b23 | -2.4 | -3.0 | 0.01 | ILMN_2976211 |
| Cyp4b1 | -2.2 | -2.8 | 0.01 | ILMN_2790496 |
| D130063H01Rik | 1.9 | 1.4 | 0.02 | ILMN_1220115 |
| D330012P07Rik | -1.5 | -1.1 | 0.02 | ILMN_2577286 |
| Diap1 | -1.1 | -1.5 | 0.02 | ILMN_2641541 |
| Dmkn | 1.8 | 1.2 | 0.02 | ILMN_3032088 |
| Dusp6 | 1.5 | 1.2 | 0.03 | ILMN_1248537 |
| E130112E08Rik | 1.2 | 1.5 | 0.03 | ILMN_1227100 |
| E130306D19Rik | 2.0 | 2.5 | 0.04 | ILMN_2659317 |
| E130309K17Rik | -1.2 | -1.5 | 0.03 | ILMN_1248622 |
| E330036I19Rik | 1.7 | 1.5 | 0.02 | ILMN_2976448 |
| Ehd3 | -1.3 | -1.6 | 0.03 | ILMN_1232989 |
| Emr1 | 1.3 | 1.8 | 0.01 | ILMN_1216880 |
| Epn2 | -1.9 | -2.5 | 0.01 | ILMN_1239027 |
| Esm1 | -3.1 | -2.2 | 0.01 | ILMN_1257574 |
| Fbn1 | 3.5 | 4.4 | 0.03 | ILMN_1223552 |
| Fgfbp1 | -1.4 | -1.5 | 0.01 | ILMN_2759371 |
| Fmod | 1.6 | 1.2 | 0.01 | ILMN_2999762 |
| Frk | 1.5 | 1.7 | 0.02 | ILMN_2615513 |
| Gal | -1.5 | -1.0 | 0.04 | ILMN_2776034 |
| Galnt2 | -1.6 | -1.2 | 0.03 | ILMN_1242677 |
| Galntl2 | -1.8 | -1.3 | 0.03 | ILMN_1244612 |
| Galntl2 | -2.2 | -1.7 | 0.02 | ILMN_1239386 |
| Gm1574 | -1.2 | -1.5 | 0.04 | ILMN_1260237 |
| Gm1574 | -1.3 | -1.6 | 0.03 | ILMN_3066123 |
| Gmppb | 1.7 | 1.5 | 0.03 | ILMN_2744909 |
| Gpnmb | 1.5 | 2.5 | 0.03 | ILMN_2648669 |
| Gulp1 | -1.6 | -1.4 | 0.03 | ILMN_2607397 |
| Hbp1 | -1.5 | -1.3 | 0.03 | ILMN_1220230 |
| Hn1 | 1.6 | 1.8 | 0.00 | ILMN_2758452 |
| Igf2r | 2.1 | 1.4 | 0.00 | ILMN_2931096 |
| Igfbp5 | 2.1 | 1.9 | 0.01 | ILMN_1254358 |
| Igl-V1 | -1.3 | 1.6 | 0.00 | ILMN_1230696 |
| Ihpk2 | 1.8 | 1.6 | 0.04 | ILMN_3055507 |
| Impdh1 | 1.9 | 2.3 | 0.02 | ILMN_1216301 |
| Kdelc1 | 1.5 | 1.7 | 0.02 | ILMN_2601147 |
| Kndc1 | -1.3 | -1.5 | 0.04 | ILMN_2757844 |
| Lama1 | 1.3 | 1.9 | 0.02 | ILMN_2973288 |
| Las1l | -1.4 | -1.6 | 0.03 | ILMN_1225876 |
| LOC100045551 | -1.5 | -1.2 | 0.02 | ILMN_1214179 |
| LOC100046608 | -1.2 | -1.6 | 0.02 | ILMN_1249366 |
| LOC233529 | 1.7 | 1.3 | 0.03 | ILMN_2533104 |
| LOC380617 | -1.6 | -1.2 | 0.04 | ILMN_1213986 |
| LOC380763 | -1.6 | -1.3 | 0.00 | ILMN_1249217 |
| LOC384422 | -0.4 | 2.1 | 0.03 | ILMN_1226583 |
| LOC385822 | 1.3 | 1.5 | 0.02 | ILMN_1223936 |
| LOC636752 | -1.3 | 1.5 | 0.00 | ILMN_1220577 |
| LOC640195 | 1.6 | 1.2 | 0.01 | ILMN_2602097 |
| LOC675572 | -1.4 | -1.6 | 0.02 | ILMN_2425028 |
| LOC677317 | 1.4 | 1.7 | 0.04 | ILMN_2774690 |
| Lyrm2 | -1.4 | -1.7 | 0.00 | ILMN_2739340 |
| Mal | 1.4 | 1.5 | 0.02 | ILMN_1248947 |
| Mapk12 | -1.2 | -1.6 | 0.00 | ILMN_1248106 |
| Mapk1ip1 | -1.8 | -1.4 | 0.01 | ILMN_3142573 |
| Mapk3 | 1.6 | 1.3 | 0.04 | ILMN_2715744 |
| Mapt | -1.2 | -1.5 | 0.03 | ILMN_3034121 |
| Marcksl1 | 2.0 | 2.7 | 0.04 | ILMN_1250438 |
| Mcam | 2.2 | 1.8 | 0.02 | ILMN_1229960 |
| Mcrs1 | 1.5 | 1.2 | 0.01 | ILMN_1257515 |
| Mfap5 | 2.4 | 2.7 | 0.02 | ILMN_1225835 |
| Mid1ip1 | 1.7 | 1.3 | 0.02 | ILMN_2847332 |
| Mmp2 | 2.2 | 3.0 | 0.01 | ILMN_2678218 |
| Mycbp2 | -1.3 | -1.7 | 0.03 | ILMN_1238676 |
| Myl9 | 1.5 | 1.2 | 0.03 | ILMN_2652458 |
| Mylc2b | -1.8 | -2.2 | 0.04 | ILMN_2710186 |
| Ncapd2 | 2.4 | 2.7 | 0.02 | ILMN_2956102 |
| Ndufb5 | 1.0 | 1.5 | 0.01 | ILMN_2617335 |
| Nkd2 | -1.5 | -1.9 | 0.00 | ILMN_1228631 |
| Nol5a | -1.9 | -1.4 | 0.04 | ILMN_1245860 |
| Nono | 1.4 | 1.8 | 0.04 | ILMN_2626779 |
| Obfc2b | 1.5 | 1.9 | 0.02 | ILMN_2593852 |
| Pabpc4 | 1.5 | 1.2 | 0.00 | ILMN_1260325 |
| Pde3a | -1.1 | -1.5 | 0.01 | ILMN_2460703 |
| Phospho2 | -1.5 | -1.1 | 0.01 | ILMN_2596007 |
| Plekhg3 | 1.6 | 1.3 | 0.02 | ILMN_2783500 |
| Ppargc1a | -1.4 | -1.6 | 0.04 | ILMN_2710139 |
| Ppm1l | -1.2 | -1.5 | 0.00 | ILMN_2419777 |
| Rn18s | 1.6 | 1.1 | 0.01 | ILMN_2638923 |
| Rnf215 | -1.4 | -1.6 | 0.01 | ILMN_2718462 |
| Rtn3 | 1.6 | 1.3 | 0.00 | ILMN_3081944 |
| Sec14l4 | -1.2 | -1.7 | 0.00 | ILMN_2605013 |
| Sfn | 2.6 | 2.1 | 0.01 | ILMN_2648169 |
| Sidt2 | 1.2 | 1.5 | 0.03 | ILMN_1258738 |
| Sirpa | 1.8 | 1.6 | 0.02 | ILMN_2722996 |
| Slc27a6 | 1.9 | 1.5 | 0.00 | ILMN_1230314 |
| Slc29a1 | -1.5 | -1.3 | 0.04 | ILMN_2638299 |
| Slc44a2 | -1.3 | -1.9 | 0.02 | ILMN_1232195 |
| Slc7a10 | -1.3 | -1.6 | 0.01 | ILMN_2705508 |
| Smug1 | -1.5 | -1.2 | 0.03 | ILMN_2620520 |
| Snai1 | 1.5 | 2.1 | 0.04 | ILMN_2904765 |
| Snrk | -1.4 | -1.7 | 0.00 | ILMN_2604894 |
| Snx18 | 1.7 | 1.3 | 0.03 | ILMN_2597886 |
| Socs3 | 1.2 | 1.6 | 0.03 | ILMN_2618176 |
| Sox7 | -1.2 | -1.6 | 0.00 | ILMN_2653251 |
| Sp5 | 1.5 | 1.8 | 0.04 | ILMN_2641201 |
| Sphk1 | -2.5 | -2.2 | 0.02 | ILMN_1232884 |
| Spnb2 | -1.5 | -1.9 | 0.02 | ILMN_1244992 |
| Stbd1 | 1.8 | 1.4 | 0.03 | ILMN_2663211 |
| Stx3 | -1.7 | -1.5 | 0.02 | ILMN_1254237 |
| Sumo1 | -1.2 | -1.6 | 0.04 | ILMN_2632942 |
| Syne2 | -1.8 | -1.9 | 0.04 | ILMN_1232181 |
| Tbxas1 | 1.2 | 1.6 | 0.03 | ILMN_1251390 |
| Tcf21 | -1.2 | -1.8 | 0.00 | ILMN_2930707 |
| Tgfb1 | 1.3 | 1.5 | 0.04 | ILMN_2711461 |
| Tmem50b | 1.5 | 1.4 | 0.03 | ILMN_2671286 |
| Tmem66 | -1.5 | -1.3 | 0.00 | ILMN_1258965 |
| Tmod2 | -1.4 | -1.7 | 0.01 | ILMN_3114546 |
| Tnfrsf11b | 1.0 | 1.5 | 0.01 | ILMN_2513826 |
| Tor1aip1 | -1.5 | -1.2 | 0.03 | ILMN_2751215 |
| Traf1 | -1.6 | -1.1 | 0.03 | ILMN_1230586 |
| Traf1 | -1.8 | -1.5 | 0.00 | ILMN_1253947 |
| Trim39 | -1.7 | -1.3 | 0.01 | ILMN_1251912 |
| Tst | 1.5 | 1.3 | 0.01 | ILMN_2493175 |
| Ttc7 | 1.5 | 1.3 | 0.04 | ILMN_2946689 |
| Ube2g1 | 1.8 | 1.5 | 0.01 | ILMN_2453620 |
| Upf2 | -1.6 | -1.4 | 0.03 | ILMN_2470211 |
| Usp48 | -1.5 | -1.7 | 0.02 | ILMN_2505095 |
| Vtn | -1.5 | -1.7 | 0.03 | ILMN_1234111 |
| Wdr7 | -1.3 | -1.5 | 0.00 | ILMN_2466601 |
| Wdr9 | -1.6 | -1.3 | 0.03 | ILMN_1252496 |
| Wfdc6b | -1.5 | -1.2 | 0.02 | ILMN_3140659 |
| Wscd1 | -2.1 | -1.7 | 0.02 | ILMN_2610442 |
| Xpnpep2 | -1.3 | -1.8 | 0.03 | ILMN_1248998 |
| Zkscan14 | -1.8 | -1.2 | 0.00 | ILMN_2497349 |
| Zmiz1 | -2.1 | -1.7 | 0.04 | ILMN_1224736 |
| Znrf1 | -1.5 | -1.2 | 0.01 | ILMN_1232073 |
